# Wireless electrical–molecular quantum signalling for cancer cell apoptosis

**DOI:** 10.1038/s41565-023-01496-y

**Published:** 2023-09-14

**Authors:** Akhil Jain, Jonathan Gosling, Shaochuang Liu, Haowei Wang, Eloise M. Stone, Sajib Chakraborty, Padma-Sheela Jayaraman, Stuart Smith, David B. Amabilino, Mark Fromhold, Yi-Tao Long, Lluïsa Pérez-García, Lyudmila Turyanska, Ruman Rahman, Frankie J. Rawson

**Affiliations:** 1 Bioelectronics Laboratory, Division of Regenerative Medicine and Cellular Therapies, School of Pharmacy, Biodiscovery Institute, University of Nottingham, Nottingham, UK; 2 Faculty of Engineering, University of Nottingham, Nottingham, UK; 3 State Key Laboratory of Analytical Chemistry for Life Science, School of Chemistry and Chemical Engineering, Nanjing University, Nanjing, China; 4 School of Pharmacy, University of Nottingham, Nottingham, UK; 5 Institute of Medical Bioinformatics and Systems Medicine, Medical Center - University of Freiburg Faculty of Medicine, University of Freiburg, Freiburg, Germany; 6 School of Medicine, Biodiscovery Institute, University of Nottingham, Nottingham, UK; 7 Children’s Brain Tumour Research Centre, School of Medicine, Biodiscovery Institute, University of Nottingham, Nottingham, UK; 8 Department of Neurosurgery, Nottingham University Hospitals, Nottingham, UK; 9 Institut de Ciència de Materials de Barcelona (ICMAB-CSIC), Campus Universitari de Cerdanyola, Barcelona, Spain; 10 School of Chemistry, University of Nottingham, Nottingham, UK; 11 School of Physics and Astronomy, University of Nottingham, Nottingham, UK; 12 Departament de Farmacologia, Toxicologia i Química Terapèutica, Facultat de Farmàcia i Ciències de l’Alimentació, Universitat de Barcelona, Barcelona, Spain; 13 Institut de Nanociència i Nanotecnologia, Universitat de Barcelona (IN2UB), Barcelona, Spain

**Keywords:** Electrochemistry, Bionanoelectronics, Nanoparticles

## Abstract

Quantum biological tunnelling for electron transfer is involved in controlling essential functions for life such as cellular respiration and homoeostasis. Understanding and controlling the quantum effects in biology has the potential to modulate biological functions. Here we merge wireless nano-electrochemical tools with cancer cells for control over electron transfer to trigger cancer cell death. Gold bipolar nanoelectrodes functionalized with redox-active cytochrome *c* and a redox mediator zinc porphyrin are developed as electric-field-stimulating bio-actuators, termed bio-nanoantennae. We show that a remote electrical input regulates electron transport between these redox molecules, which results in quantum biological tunnelling for electron transfer to trigger apoptosis in patient-derived cancer cells in a selective manner. Transcriptomics data show that the electric-field-induced bio-nanoantenna targets the cancer cells in a unique manner, representing electrically induced control of molecular signalling. The work shows the potential of quantum-based medical diagnostics and treatments.

## Abstract (web summary)

Quantum biological electron transfer has potential in diagnostic and therapeutic settings. Here the authors report the triggered apoptosis of cancer cells using electricical input to wirelessly induce redox interactions at bio-nanoantennae in proximity to cancer cells.

## Main

We are entering an era where it has been realized that bioelectricity, defined as the electrical language of cells, programs cell function^1,2^. The cell is increasingly viewed as a mass of bioelectrical interconnected circuits that use an endogenous current generated by electron transfer processes to communicate with each other to maintain homoeostasis^3^. These electron transfer processes in biology, when governed by quantum mechanical effects such as electron tunnelling, are classified as quantum biological tunnelling for electron transfer (QBET). For instance, one of the most well-known electron transfer pathways is photosynthesis, the mechanism of which was one of the first to be linked to quantum mechanical effects^4^. Cytochrome *c* (Cyt *c*) is known for its vital role in the mitochondrial electron transfer chains that are governed by the redox activity (Fe^2+^ to Fe^3+^, and vice versa) of the haem ring present in its structure^5^. These redox processes in Cyt *c* are also known to occur via electron tunnelling^6–8^ and are regulated by the Warburg effect, which is underpinned by bioelectrical faradaic currents^9,10^. Furthermore, these processes are crucial for the translocation of Cyt *c* to the cytosol^11^ and for modulating its binding conformation^12^ for interaction with apoptotic protease activating factor 1 (APAF-1)^13^. Although quantum biology is still in its infancy, we envisage future interdisciplinary developments, which will be reinforced by the understanding and modulation of these quantum mechanical processes^14^. This highlights the need for technology to electrically interact with redox molecules such as Cyt *c* whose functions are supported by quantum mechanical processes.

Remotely induced electrical–molecular communication (electrical input causing a redox change in a targeted molecule, inducing an alteration in cell behaviour) inside cells opens the possibility of creating disruptive technologies, including the development of quantum medicines for cancer treatments^15^. However, on-demand targeted electrical–molecular communication within cells has yet to be realized. This is a result of the lack of technological innovation that is suitable for interfacing with cells in a medium at a spatial/temporal level in which native biological interaction occurs. The aim of this study is to pioneer solutions to these challenges.

**Fig. 1:**
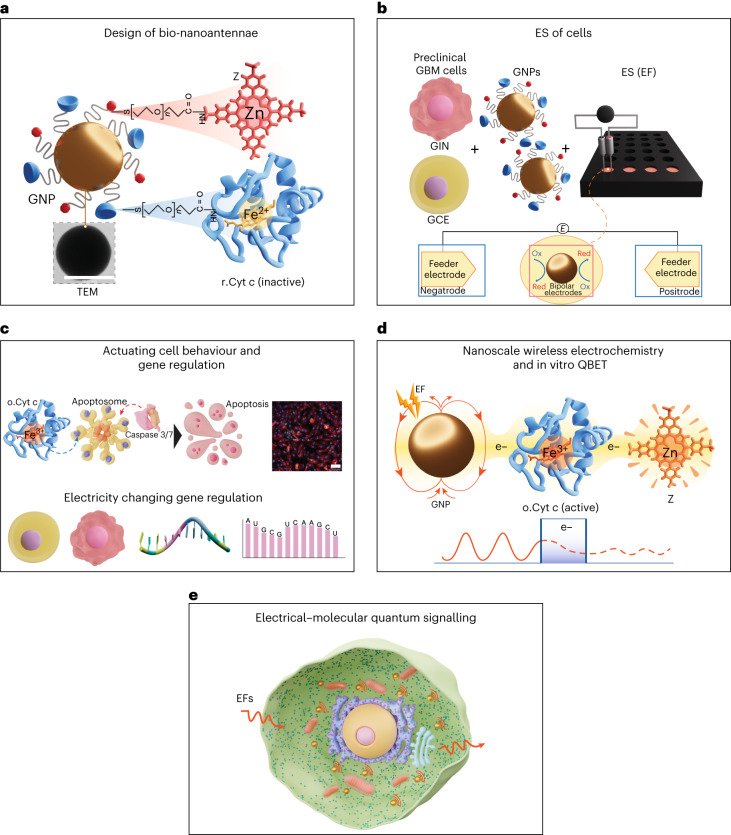
Illustration of wireless electrical–molecular quantum signalling mediated by a.c.-EF-responsive bio-nanoantennae to induce cell death. **a**, Bio-nanoantennae (GNP100@r.Cyt *c*@Z) were synthesized by covalently conjugating r.Cyt *c* and Z to carboxylic GNP100s using *N*-(3-dimethylaminopropyl)-N′-ethylcarbodiimide hydrochloride (EDC)/*N*-hydroxysuccinimide (NHS) chemistry. **b**, Primary patient-derived GBM cells, namely, GIN (derived from the GBM infiltrative margin) and GCE (derived from the GBM proliferative core) cells, were incubated with bio-nanoantennae to enable their uptake. These GBM cells were electrically stimulated with a.c. EFs of 3 MHz at 0.65 V cm^–1^. V, voltage; ES, electrical stimulation; Ox, oxidized; Red, reduced. **c**, These applied a.c. EFs caused the intracellular wireless electrochemistry to occur at the bio-nanoantennae surface to induce caspase-3/7-mediated apoptosis of GBM cells. We examined the electrical–molecular signalling by connecting gene regulation with the a.c.-EF-mediated cell death in GBM cells. **d**, The a.c. EFs were applied to induce wireless electrochemistry at the surface of bio-nanoantennae to wirelessly switch the redox state of Cyt *c* (reduced to oxidized), ex situ, suggesting QBET in the proposed system. e^–^, electron. **e**, Diagram representing the a.c.-EF-responsive bio-nanoantennae for electrical–molecular quantum signalling with GBM cells. Protein Data Bank (PDB) structural entries were used to represent Cyt *c* (PDB 1HRC; refs. ^39,40^), apoptosome (PDB 3J2T; refs. ^41,42^) and caspase 3/7 (PDB 3GJQ; refs. ^43,44^). Chemspider structure no. 19989078 (ref. ^45^) was used to represent Z. Structure illustrations by Leonora Martínez Nuñez. **a**,**d**, Z from ref. ^45^; **a**,**c**,**d**, Cyt *c* from refs. ^39^^,^^40^; **c**, Apoptosome from refs. ^41^^,^^42^; Caspase 3/7 from refs. ^43^^,^^44^.

Gold nanoparticles (GNPs) and carbon nanotubes can behave as bipolar nanoelectrodes^16,17^ within cells when external electric fields (EFs) are applied. The bipolar nanoelectrodes become polarized when an EF is applied, leading to a voltage gradient across the particle^18^. With a sufficiently large potential difference between the poles of the electrode, the thermodynamic driving force can cause electrochemically induced redox reactions to occur by virtue of bipolar electrochemistry (also known as wireless electrochemistry)^19,20^. It was thought that nanoscale wireless electrochemistry was not possible in the presence of cells even at very high applied voltages^21,22^. Importantly, most recently, bipolar electrodes in series lead to a dramatic drop in cell impedance^23^, which may allow electrical inputs to be used without inducing cell damage. Redox reactions at carbon nanotube porins, acting as bipolar nanoelectrodes, can occur at unprecedentedly low applied voltages in cells without inducing direct cell death^24^. We, therefore, designed an approach that uses bifunctionalized bipolar nanoelectrodes with attached redox-active molecules, which we term bio-nanoantennae (they are capable of receiving a remotely applied external EF input and converting this to bio-signalling events). Furthermore, alternating current (a.c.) EFs are reported to notably lower the impedance of the cell membrane at higher frequencies to penetrate the cytoplasm^25,26^, which together with the enhanced voltage gradient observed at the nanoscale would facilitate bipolar electrochemistry^27^. Therefore, we hypothesized that these bio-nanoantennae in combination with applied a.c. EFs could be used to modulate electron transfer, which then could be converted into molecular actuation via targeting a specific metabolic pathway (Fig. 1).

The strategy to fulfil the development of a quantum electrical–molecular communication tool was multipronged and inspired by the observation that electron transfer in Cyt *c* is mediated through QBET^8^. Therefore, we functionalized GNPs with an electron-donor-reduced Cyt *c* (r.Cyt *c*) and a redox mediator, zinc porphyrin (zinc 5-(4-aminophenyl)-10,15,20-(tri-4-sulfonatophenyl)porphyrin triammonium; Z). When these particles are exposed to an electrical input, a voltage gradient is induced at the surface; these are therefore described as electronic bio-nanoantennae (Fig. 1a). We show that the electrical input confers specific signalling in cells via the bio-nanoantennae to induce apoptosis-mediated glioblastoma (GBM) cell death. The transcriptomic analysis enabled elucidation of the biochemical signalling when using the electrical–molecular communication tool (Fig. 1b,c). We show that on the application of a resonant electrical input (a.c. EF, 3 MHz, 0.65 V cm^–1^), wireless electrochemistry is induced at the bio-nanoantennae surface and switches the redox state of the Cyt *c*. We propose that the QBET phenomenon and resonant electron transfer between the Cyt *c* and Z facilitate cellular apoptosis (Fig. 1d). The data suggest a.c.-EF-induced molecular actuation via bio-nanoantennae in the treatment of a disease, which is a quantum functional medicine tool (Fig. 1e). This could lead to exciting opportunities for the development of quantum nanomedicines and cancer treatments and provide a state-of-the-art means of modulating cell metabolism.

## Design of bio-nanoantennae for actuating cell death

To electrically communicate with cells at a level equivalent to that underpinning molecular biochemistry, we initially used carboxylic PEG modified 100 nm spherical GNPs (GNP100s; PEG, polyethylene glycol) as bipolar nanoelectrodes. These GNP100s can sense an EF applied extracellularly^16,23^. This occurs when they are functionalized with redox-active biomolecules to yield a bio-nanoantenna that can enable surface redox reactions (via wireless electrochemistry) to actuate a cell-specific signalling pathway. Apoptosis is mediated (in part) by Cyt *c* bioelectrochemistry in which the oxidized state (Fe^3+^) facilitates apoptosis-mediated cell death^12^. Therefore, we conjugated GNP100 with an electrical donor, that is, r.Cyt *c* (inactive, Fe^2+^), and a redox mediator Z using carbodiimide coupling chemistry to form bio-nanoantennae (GNP100@r.Cyt *c*@Z). On application of a resonant a.c. EF, the bio-nanoantennae would polarize, providing the thermodynamic driving force for nanoscale wireless electrochemistry to occur and switch the redox state of r.Cyt *c* from Fe^2+^ to Fe^3+^ (oxidized Cyt *c* or o.Cyt *c*)^28^.

**Fig. 2:**
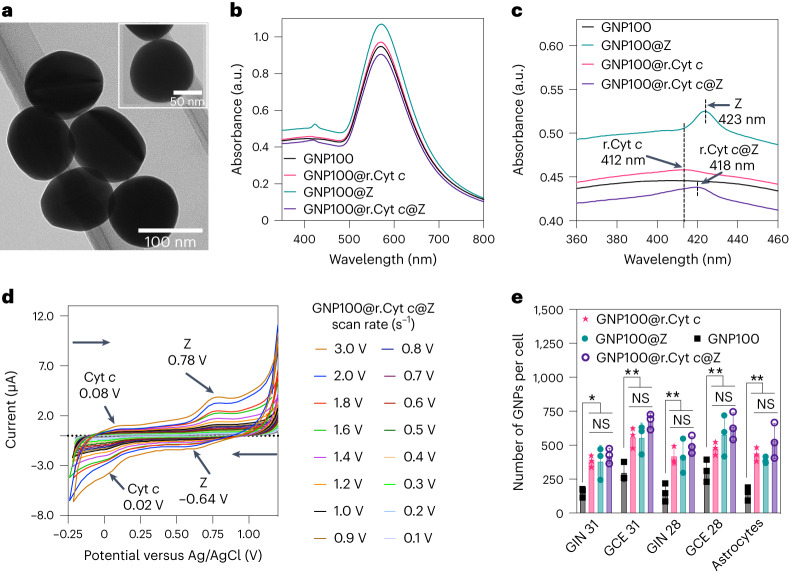
Physico-chemical and electro-analytical characterization of bio-nanoantennae and their interaction with patient-derived GBM cells. **a**, TEM image of bio-nanoantennae (GNP100@r.Cyt *c*@Z) prepared by coupling r.Cyt *c* and Z to GNP100s. The inset is a high-resolution TEM image of 100 nm bio-nanoantennae. The TEM analysis was done on three different samples of bio-nanoantennae that were synthesized over the course of three individual experiments. **b**, UV–visible absorption spectra of bio-nanoantennae dispersed in phosphate buffer saline (PBS) before ES. **c**, Zoomed-in UV–visible absorption spectra from 360 to 460 nm showing surface functionalization with r.Cyt *c* (GNP100@r.Cyt *c*), Z (GNP100@Z) and both r.Cyt *c* and Z (GNP100@r.Cyt *c*@Z). **d**, Cyclic voltammetry scan rate studies to analyze the redox properties of GNP100@r.Cyt *c*@Z. Redox potentials were measured using an ITO working electrode, platinum wire counter electrode and Ag/AgCl reference electrode with samples (25 µg mL^–1^) dispersed in 10 mM PBS, scanned from +1.2 V to –0.25 V. ITO, indium tin oxide. **e**, ICP-MS analysis to determine the association of bio-nanoantennae with different patient-derived GBM cells and cortical astrocytes; the data are expressed as the number of GNPs per cell. Results are expressed as ±s.d. of the mean obtained from a triplicate experiment and repeated three times. The data were considered significant if *P ≤ 0.05, **P ≤ 0.01, ***P ≤ 0.001 and ***P ≤ 0.0001 versus GNP100, obtained using two-way analysis of variance (ANOVA) with a Tukey post-test. NS, not significant. Source data

The successful bifunctionalization of carboxylic PEG modified GNP100s (2 kDa PEG) with r.Cyt *c* and Z is evident from transmission electron microscopy (TEM) images (Fig. 2a), dynamic light scattering (Supplementary Fig. 1) and zeta potential (Supplementary Fig. 2) and UV–visible (Fig. 2b,c and Supplementary Fig. 3a–e) characterizations of GNP100@r.Cyt *c*@Z. A quantification of redox molecules bound to each nanoparticle is presented in Supplementary Tables 1–4. Cyclic voltammetry was carried out to study the redox behaviour of r.Cyt *c* and Z on a bifunctionalized system (Fig. 2d and Supplementary Fig. 4a–c). The heterogeneous electron transfer rate coefficient (*k*^0^) of Cyt *c* for GNP100@r.Cyt *c* was calculated to be 9.6 × 10^−3^ cm s^–1^ (Supplementary Table 5), while that for GNP100@r.Cyt *c*@Z was 3.75 × 10^−3^ cm s^–1^ (Supplementary Fig. 5a–f), suggesting a slight decrease in the electron transfer rate of Cyt *c* in a bifunctionalized system. A detailed discussion is in Supplementary Note 1.

To explore the potential of bio-nanoantennae on the a.c.-EF-mediated redox switching of Cyt *c*, first we investigated the association and uptake of bio-nanoantennae on four types of preclinical GBM cells, isolated from two GBM patients, glioma invasive margin (GIN 28 and GIN 31) and glioma contrast-enhanced core (GCE 28 and GCE 31), which echo similar characteristics to different regions of GBM tumours^29^, and a commercial GBM cancer cell line, U251. Human-derived cortical astrocytes were also included as a control for non-tumorigenic cells. A three-dimensional analysis of *z*-stack confocal microscopy images (Supplementary Fig. 6) confirmed that the bio-nanoantennae were internalized by all types of cells after 8 hours of incubation, and the number per cell was quantified using inductively coupled plasma mass spectrometry (ICP-MS; Fig. 2e). PrestoBlue assay data revealed that the bio-nanoantennae are biocompatible up to a tested range of 100 µg mL^–1^ (Supplementary Fig. 7).

## EF responsive bio-nanoantennae induce apoptosis in GBM

**Fig. 3:**
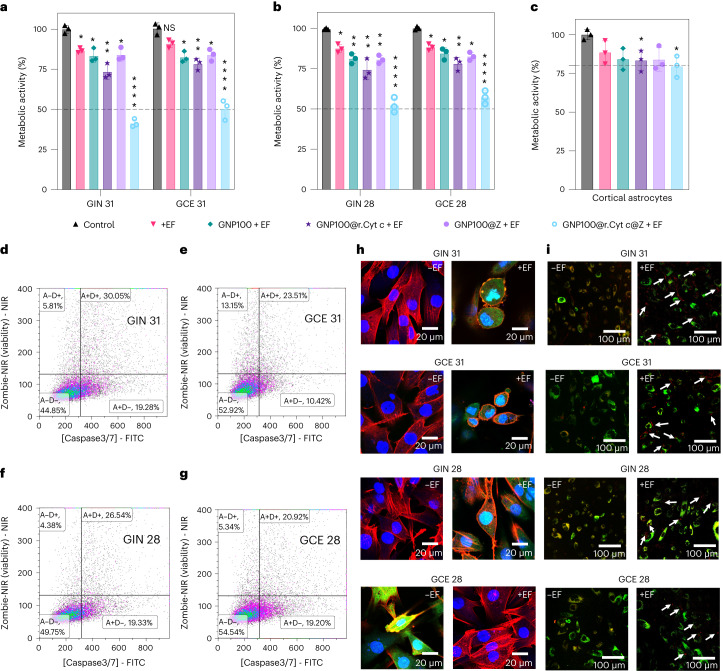
Wireless electrical–molecular communication mediated by a.c.-EF-responsive bio-nanoantennae induces caspase-3/7-mediated apoptosis in preclinical GBM cells. **a**–**c**, Metabolic activity of GIN/GCE 31, GIN/GCE 28 and cortical astrocytes was analyzed using a PrestoBlue HS assay. GIN cells, GCE cells and human cortical astrocytes were treated with GNP100@r.Cyt *c*@Z for 8 h followed by a.c. EF stimulation (3 MHz, 0.65 V cm^–1^) for 12 h. The control is no treatment with either bio-nanoantennae or a.c. EFs. Error bars represent mean ± standard error of mean (s.e.m.) obtained from triplicate experiments repeated three times. Statistical analysis was performed by applying a two-way ANOVA with a Tukey’s post-test. **d**–**g**, Representative samples from flow cytometric analysis of cells stained with CellEvent Caspase-3/7 Green to detect caspase 3/7 apoptotic activity and Zombie NIR fixable dye to detect the dead cell population. The quadrants in the figure represents the following: A-D-, no cell death either due to caspase 3/7 apoptosis or necrosis; A+D-, caspase 3/7 positive apoptotic cells; A+D+, non-viable/dead cells caused by apoptosis; and A−D+, necrotic cells. The gating strategy is shown in Supplementary Fig. 14. FITC, fluorescein isothiocyanate and NIR, near infra-red are filters. **h**, High-magnification confocal microscopy images to demonstrate caspase 3/7 activation immediately after the treatment with a.c. EFs (3 MHz, 0.65 V cm^–1^) for 12 h in the presence of bio-nanoantennae. Cells were fixed with paraformaldehyde followed by counterstaining with a caspase 3/7 detection kit (green), Cytopainter actin phalloidin (Texas Red 591, in red) and Hoechst nuclear stain (blue). Scale bars, 20 µm. To confirm caspase 3/7 activation, at least five confocal images (each with nearly 30 cells) were taken at ×10. High-resolution (×63) images show both morphology and caspase 3/7 activation (in green). The –EF and +EF indicate before and after the EF treatment. **i**, Confocal microscopy images to demonstrate the cytoplasmic localization of GNP100@r.Cyt *c*@Z immediately after the treatment with a.c. EFs (3 MHz, 0.65 V cm^–1^) for 12 h in the presence of bio-nanoantennae. Cells were stained with late endosome dye (green) and imaged using a Leica confocal microscope with green fluorescent protein, GFP (late endosomes) and Alexa 633 (GNP100@r.Cyt *c*@Z) filter settings. Scale bars, 100 µm. White arrows represent the endosomal escape and localization of bio-nanoantennae, at least 60 cells were analysed. Source data

To examine the electrical input that can be sensed by intracellular bio-nanoantennae for inducing wireless electrochemistry, we optimized the voltage and frequency (Supplementary Fig. 8) by assessing the change in metabolic activity of GIN 31 cells as the preliminary read-out of Cyt *c* redox switching. The maximum effect on the metabolic activity was observed at 3 MHz, at an applied potential of 1 V cm^–1^ or 0.65 V cm^–1^ (no significant difference between 0.65 V cm^–1^ and 1 V cm^–1^; *P* value = 0.23), with significant differences between the tested controls (GNP100, GNP100@r.Cyt *c* and GNP100@Z) and the bifunctionalized bio-nanoantennae (GNP100@r.Cyt *c*@Z). Importantly, the observed decrease in metabolic activity of GNP100@r.Cyt *c*@Z-treated cells was significantly higher than that following 24 h application of in vitro tumour-treating fields that have been approved by the US Food and Drug Administration^30,31^. We ascribe this decrease in metabolic activity to the electrical–molecular communication via redox switching of r.Cyt *c* to o.Cyt *c*, thus inducing cell stress. To eliminate any potential effect of the applied 1 V on water electrolysis (1.23 V versus normal hydrogen electrode), we chose 0.65 V cm^–1^ for further studies. The response of the cells to the treatment with bio-nanoantennae (GNP100@r.Cyt *c*@Z) was comparable in different patient-derived GIN and GCE cells, as well as in the U251 cell line (Fig. 3a,b and Supplementary Fig. 9), with a reported, ~50% decrease in metabolic activity achieved after 12 h compared to ~20% decrease after 2 h of a.c. EF stimulation (Supplementary Fig. 10). This decrease in metabolic activity was significantly higher compared to all other experimental controls (*P* values obtained from the statistical analysis are listed in Supplementary Table 6). By contrast, a weaker effect (~20% decrease in metabolic activity) was observed in cortical astrocytes (Fig. 3c; *P* value = 0.011), which was found to be significantly different from the control (no treatment with either bio-nanoantennae or a.c. EFs). A brief discussion on the response of other normal cells (liver and cerebellar astrocyte; Supplementary Fig. 11a,b) to the treatment can be found in Supplementary Note 2. Thus, based on the obtained data, we conclude that the change in metabolic activity depends on the duration of treatment and cell type.

The alteration in metabolic activity in cells treated with GNP100@r.Cyt *c*@Z plus a.c. EFs (3 MHz at 0.65 V cm^–1^ for 12 hours) is correlated with increased cell death, as clearly evidenced by the results of a live–dead assay (Supplementary Figs. 12 and 13). The mechanism of cell death was probed by flow cytometry (Fig. 3d–g and Supplementary Figs. 14–16) and confocal microscopy (Fig. 3h and Supplementary Fig. 17), where the induced caspase 3/7 activity was observed in GIN/GCE cells treated with GNP100@r.Cyt *c*@Z followed by electrical stimulation (ES), indicative of apoptosis. This could be attributed to the increased affinity of activated Cyt *c* (Fe^3+^) on bio-nanoantennae with APAF-1 (ref. ^12^). Confocal microscopy revealed that bio-nanoantennae, upon ES, escape the endo/lysosomal degradation to localize in the cytosol (Fig. 3i and Supplementary Fig. 18). Further studies are required to investigate the precise mechanism. We note that no reactive oxygen species and temperature changes were observed (Supplementary Figs. 19 and 20). Therefore, we successfully conducted the ES of GBM cells and demonstrated the wireless electrochemistry-mediated redox switching and activation of Cyt *c* on the surface of GNP100@r.Cyt *c*@Z, leading to the apoptosis of GBM cells.

**Fig. 4:**
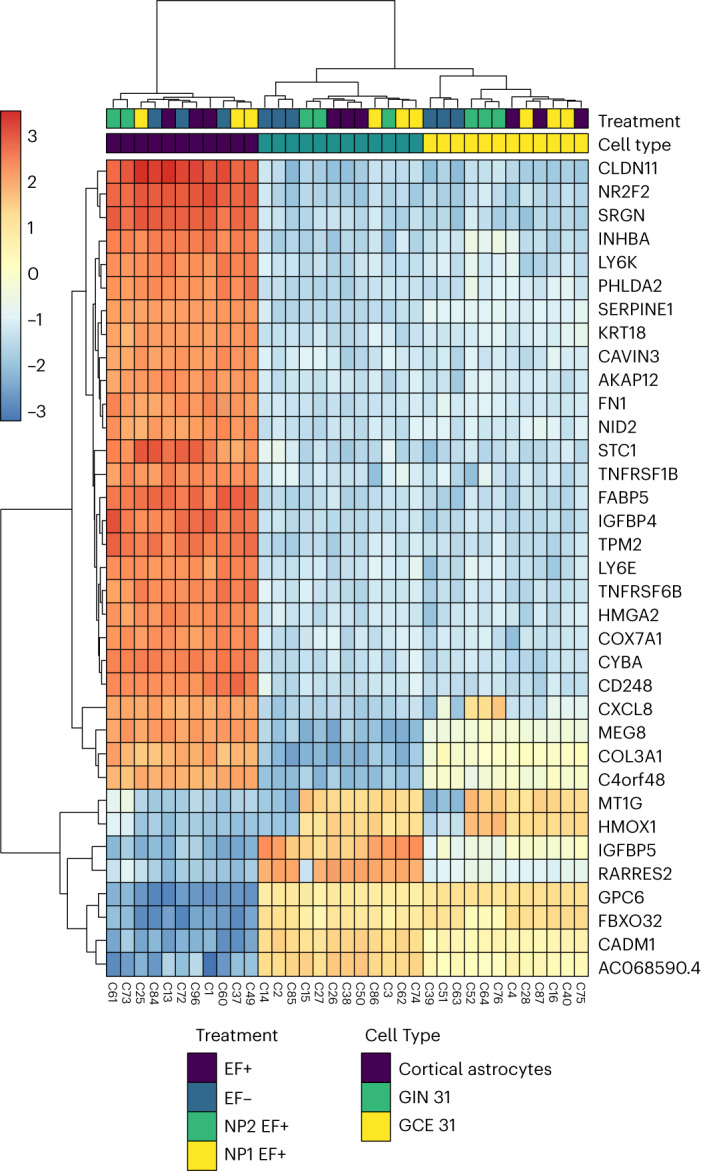
Transcriptomic analysis to connect bio-nanoantennae-mediated wireless electrical -molecular communication with gene expression and regulation. Heat map demonstrating hierarchical clustering of top 35 genes that were regulated after the treatment with GNP100@r.Cyt *c*@Z for 8 h followed by a.c. EF stimulation (3 MHz, 0.65 V cm^–1^) for 2 h. A variance-stabilized transformation was performed on the raw count matrix, and 35 genes with the highest variance across samples were selected for hierarchical clustering. Each row represents one gene, and each column represents one sample. The colour represents the difference of the count value to the row mean. GIN 31 and GCE 31, which showed the maximum response to the treatment with GNP100@r.Cyt *c*@Z and a.c. EFs, were chosen. As a control to cancer cells, healthy cortical astrocytes were used. Immediately after the treatment, cells were washed and centrifuged to obtain a pellet, which was snap-frozen in liquid nitrogen and shipped (in dry ice) to Qiagen in Germany for RNA sequencing. The treatment codes are as follow: EF–, control (no treatment with either bio-nanoantennae or a.c. EFs); EF+, cells treated with a.c. EFs; NP1 EF+, cells treated with GNP100@r.Cyt *c*; and NP2 EF+, cells treated with GNP100@r.Cyt *c*@Z; treatment was for 8 h followed by 2 h with the a.c. EF. Source data

**Extended Data Fig. 1:**
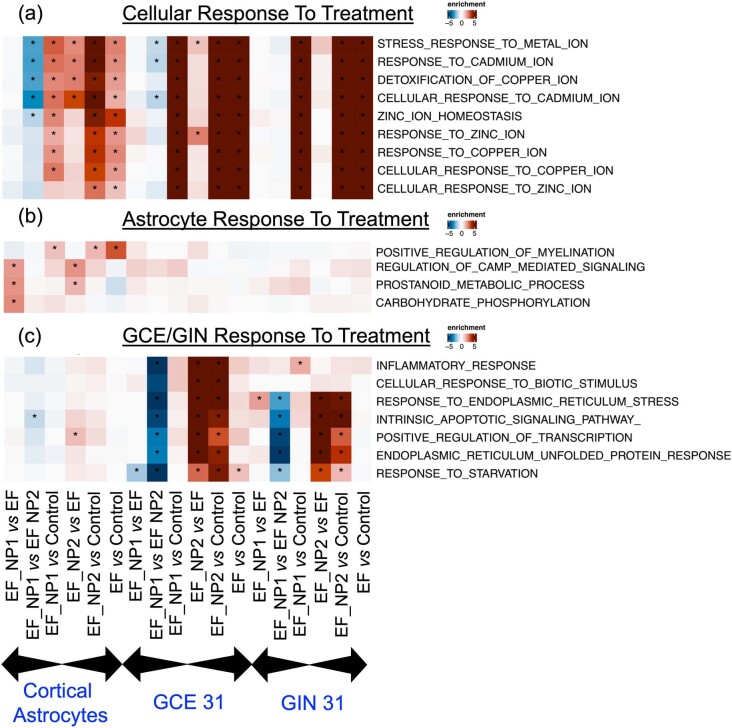
Gene-set Enrichment analysis (GSEA) of Gene Ontology Biological Processes (GOBP). **(a)** GSEA of GOBP across all cell-lines (Cortical-Astrocytes, GCE-31, and GIN-31) is represented as a heatmap. The topmost GOBP terms based on the adjusted p-value are shown. **(b)** Cortical Astrocyte Response to Treatment: GSEA of GOBP across all cell-lines (Cortical-Astrocytes, GCE-31, and GIN-31) is represented as a heatmap. The topmost GOBP terms that are differentially regulated in cortical astrocytes (based on the adjusted p-value) compared to GCE-31 and GIN-31 cell-lines are shown. **(c)** GCE/GIN 31 Cells Response to Treatment: GSEA of GOBP across all cell-lines (Cortical-Astrocytes, GCE-31, and GIN-31) is represented as a heatmap. The topmost GOBP terms that are differentially regulated in GCE-31 and GIN-31 cell-lines (based on the adjusted *p*-value) compared to cortical astrocytes are shown. In Fig. b-d: the colour code is based on the normalized enrichment score (NES). The significantly enriched terms (adj *p*-value < 0.05) are indicated by (*) symbol. For the GSEA, we used one-tailed Fisher’s exact test with 95% of confidence interval. The p-values were *corrected* for *multiple comparisons* with the Benjamini-Hochberg method. The exact *p* values can be found in Supplementary Table 7. The treatment codes are as follow: Control = no treatment with either bio-nanoantennae or AC EFs); EF = cells treated with AC EFs; EF_NP1 = cell treated with GNP100@r.Cyt *c*, and EF_NP2 = cells treated with GNP100@r.Cyt *c*@Z, for 8 h followed by 2-hour a.c. EFs. Source data

To further understand this electrical–molecular signalling, we performed transcriptomic analysis on a sample of GBM cells and healthy cortical astrocytes (Fig. 4 and Supplementary Figs. 21 and 22**)** to explore the effect of the bioelectronic communication tool on gene expression and regulation. Hierarchical clustering analysis showed a differential expression of genes related to apoptosis, cancer proliferation and angiogenesis, and tumour suppression (Fig. 4**)**. This change in differentially expressed genes was highest for GIN/GCE 31 cells treated with GNP100@r.Cyt *c*@Z and followed by ES compared to untreated cells and other experimental controls. Interestingly, we observed minimal changes in the gene regulation for astrocytes, which suggests that the astrocytic transcriptomic landscape remains largely unperturbed upon the treatment. By contrast, an important deviation in the transcriptome has been monitored for GBM cell lines, implying that the treatment modulates signalling pathways that are specific only to GBM. To explain this effect, we conducted gene-set enrichment analysis of gene ontology biological processes (Extended Data Fig. 1 and Supplementary Figs. 23 and 24). A detailed discussion on transcriptomics data and gene ontology biological process analysis is in Supplementary Note 3. Collectively, the data obtained from Figs. 3–4 and Extended Data Fig. 1 imply that GNP100@r.Cyt *c*@Z treatment followed by ES leads to reduced proliferation and apoptosis induction in patient-derived GBM cells. Overall, the in vitro results indicate successful communication with biology at a molecular scale using electricity. Importantly, this approach enables selective actuation of cancer cell behaviour compared to normal healthy cells (astrocytes and liver bile duct cells).

## Nanoscale wireless electrochemistry and in vitro QBET

**Fig. 5:**
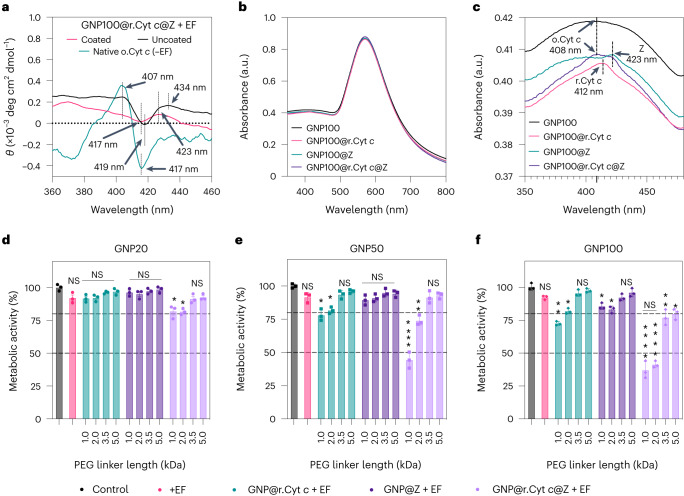
Nanoscale wireless electrochemistry and in vitro electron tunnelling via bio-nanoantennae for inducing cell death in GBM cells. **a**, The effect of a.c. EFs on the conformation of redox moieties**:** circular dichroism spectra in the Soret region to emphasize the redox-mediated change in haem moieties of free native o.Cyt *c*, r.Cyt *c* and bifunctionalized GNP bio-nanoantennae in 10 mM PBS (pH 7.4).θ, molar ellipticity. All samples with identical concentrations (25 µg mL^–1^) were used for spectrum acquisition. Three spectra of each sample were collected and averaged. Insulated, coated steel electrodes were used as a control to demonstrate the occurrence of wireless electrochemistry at the nanoscale. **b**,**c**, UV–visible spectra of bio-nanoantennae after stimulation with a.c. EFs of 3 MHz, 0.65 V cm^–1^ indicating a blueshift in the absorption maxima in GNP100@r.Cyt *c*@Z, indicating the redox switching of Cyt *c* (from r.Cyt *c* to o.Cyt *c*). **d**–**f**, Metabolic activity of GIN 31 cells as a function of different-sized bio-nanoantennae (20 nm, 50 nm and 100 nm; GNP20@r.Cyt *c*@Z, GNP50@r.Cyt *c*@Z and GNP100@r.Cyt *c*@Z, respectively) synthesized using various linker lengths (1, 2, 3.5 and 5 kDa). GIN 31 cells were treated with bifunctionalized bio-nanoantennae for 8 h followed by a.c. EF stimulation (3 MHz, 0.65 V cm^–1^) for 12 h. Error bars represent ±s.e.m. of the mean obtained from triplicate experiments repeated three times. Statistical analysis was performed by applying a two-way ANOVA with a Tukey’s post-test. Exact *P* values are in Supplementary Table 7. Source data

The electrical–molecular communication via wireless electrochemistry induced at bio-nanoantennae was probed by circular dichroism analysis (Fig. 5a and Supplementary Fig. 25) and UV–visible absorption spectroscopy (Fig. 5b,c and Supplementary Figs. 26 and 27). There was no significant change in the hydrodynamic diameter *h*_d_ and zeta potential *ζ* after ES with an a.c. EF (Supplementary Figs. 28 and 29). A detailed discussion on the data demonstrating that the bio-nanoantennae act as bipolar electrodes and can modulate the redox state of the Cyt *c* under application of the EF is in Supplementary Note 4.

To facilitate the translation of the technology more broadly, understanding of the electrically induced mode of electron transfer is needed. The frequency we used for electrical communication was 3 MHz; considering the electron transfer rate constant of 3.75 × 10^−3^ cm s^–1^ calculated from Fig. 2d, the maximum distance the electron could travel at this frequency is 0.01 nm. Thus, theoretically at this given frequency, the redox event should not occur. We propose that the observed activity is enabled by QBET induced from the Cyt *c*, as was indicated previously^8^. However, this process has not been controlled using ES.

To gather experimental evidence for QBET in our system, we consider molecular tunnel junctions, where quantum electron tunnelling can occur^32,33^. We altered the tunnel junction energy by using GNPs with different sizes (20, 50 and 100 nm) and a PEG linker with different lengths (1, 2, 3.5 and 5 kDa; Supplementary Figs. 30 and 31). Toxicity studies of different-sized bio-nanoantennae synthesized using PEG linkers of various lengths without ES at 25 µg mL^–1^ showed no significant changes in the metabolic activity compared to an untreated control (Supplementary Fig. 32**)**. The metabolic assays (Fig. 5d–f, Supplementary Table 7 and Supplementary Fig. 33) and viability studies (Supplementary Figs. 34 and 35) on GIN 31 and GCE 31 cells with ES (3 MHz and 0.65 V cm^–1^) indicated a resonant biological effect with bio-nanoantennae using the 1 and 2 kDa PEG linkers with 50 nm and 100 nm GNPs. Importantly, this effect is seen only for cells treated with bio-nanoantennae (GNP@r.Cyt *c*@Z) following 12 h of ES. ICP-MS analysis revealed no significant difference in the number of Z or r.Cyt *c* per cell when treated with GNP20@r.Cyt *c*@Z, GNP50@r.Cyt *c*@Z or GNP100@r.Cyt *c*@Z (Supplementary Figs. 36 and 37**)**. We propose that a QBET is occurring in our system, as evidenced by the resonance at specific conditions, and is indicative of wave-like behaviour^8,34^. Thus, we suggest that the electron transfer in this system is an example of electrically stimulated QBET in biology and consequently an example of facilitating electrical–molecular communication.

**Fig. 6:**
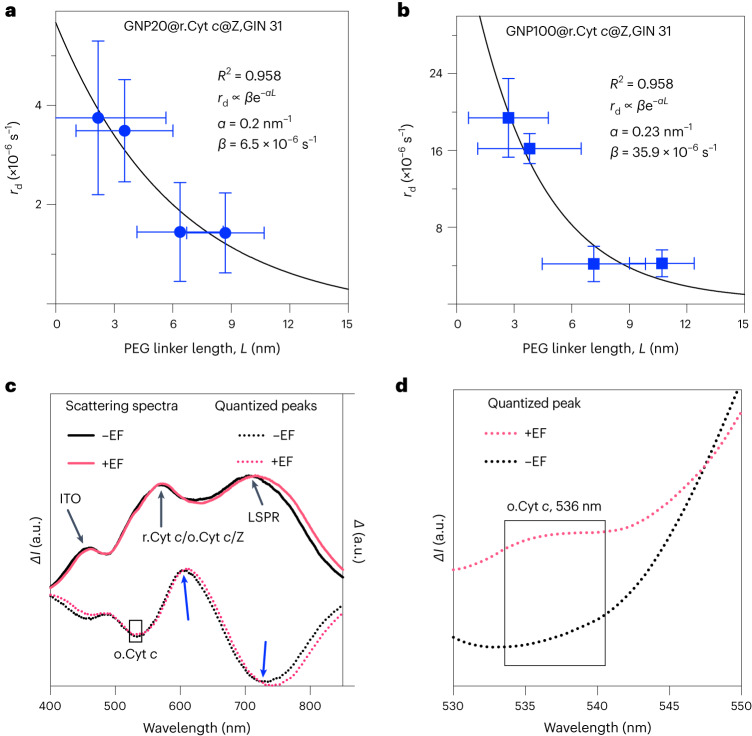
Mathematical modelling and plasmon resonance energy transfer analysis for probing quantum tunnelling in bio-nanoantennae system. **a**,**b**, Mathematic modelling to determine the rate of donor charging, *r*_d_, calculated using the metabolic activity, using equation (1), compared to the PEG linker length, *L*, for GNP20@r.Cyt *c*@Z (**a**) and GNP100@r.Cyt *c*@Z (**b**) in GIN 31 cell samples. The black lines show the exponential behaviour expected for quantum tunnelling with an inverse localization radius *α* and constant of proportionality *β*, shown in the figure legends. Error bars represent ±s.d. of the mean. The *x*-axis error bar represents the s.d. of the mean PEG linker length, and the *y*-axis error bar represents the s.d. of the mean *r*_d_ (rate of Cyt *c* charging), which was obtained from equations (5)–(7) in the Methods. R^2^, coefficient of determination. **c**, Scattering spectra (*I*, intensity) and spectra difference (*Δ*) for QBET obtained for GNP100@r.Cyt *c*@Z bio-nanoantennae. The quantized peaks were obtained from the difference of scattering spectra between the samples functionalized with r.Cyt *c* and Z using a 2 kDa linker and GNP100. Solid curves are captured scattering spectra (linked to left axis) of GNP100@r.Cyt *c*@Z, and dashed curves are quantized peaks, that is, the corresponding spectra difference (linked to right axis). Blue arrows indicate a peak shift. LSPR, localized surface plasmon resonance. **d**, Quantized peaks of GNP100@r.Cyt *c*@Z within a 530–550 nm region (zoomed-in from **c**) confirming the presence of o.Cyt *c* in samples exposed to a.c. EFs. Source data

To provide further evidence of the mechanism of electron transfer, we established a mathematical model. The cell metabolic rate (shown in Fig. 5d–f) was correlated to the rate of charge transfer from Cyt *c*, derived as Equation (1) (full details of the derivation are presented in the Methods). These values were then plotted as a function of the barrier junction, altered using the PEG ligands with different length and different-sized nanoparticles (Fig. 6a,b).

1 $${r}_{{\mathrm{d}}}=-\frac{{\rm{ln}}\left(M\left(t\right)\right)}{t}$$

where *r*_d_ is the rate of donor charging and *M*(*t*) is the metabolic activity at time *t*.

**Extended Data Fig. 2:**
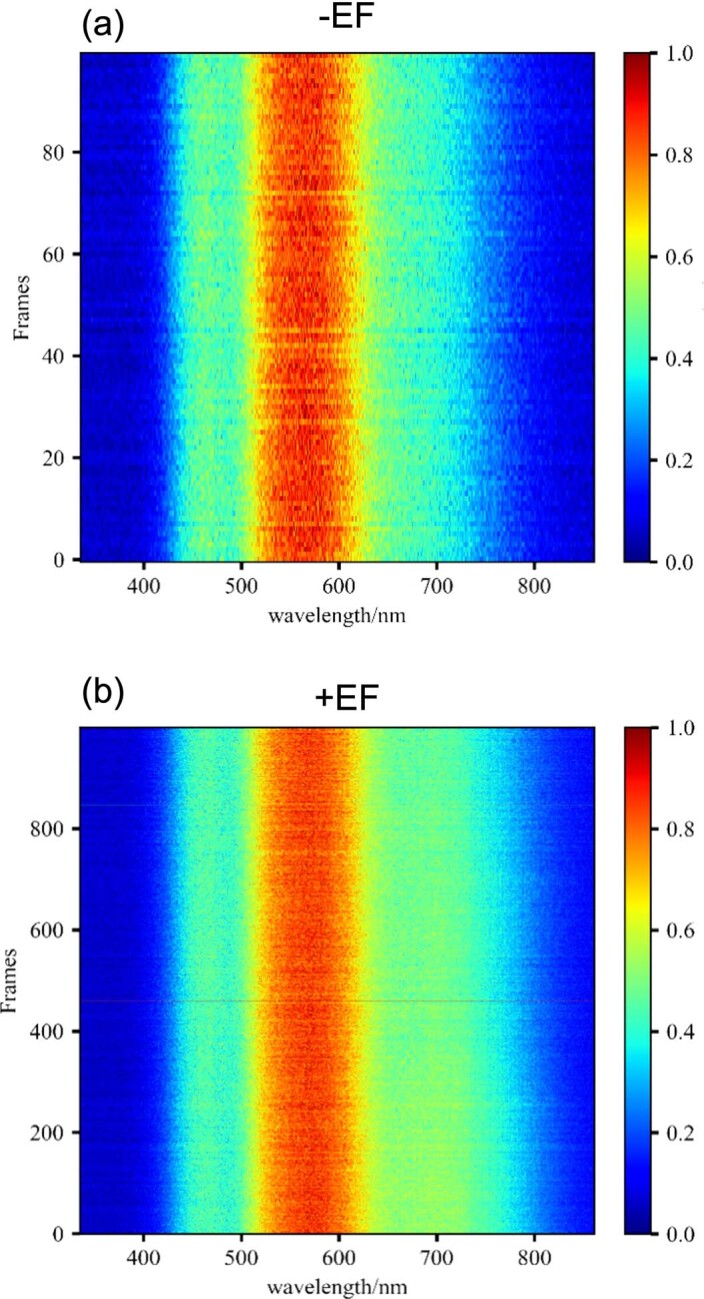
Plasmon resonance scattering spectra and quantized peaks to demonstrate QBET. Two-dimensional heat map of scattering spectral changes obtained from dark field microscopy, **(a)** in the absence of EF and **(b**) presence of EF.

The results fit well with the exponential dependences expected for the tunnelling of an electron through a barrier^35^, supporting the hypothesis that the resonant biological effects observed with the electrical input result in QBET. Further primary experimental evidence for the quantum tunnelling is provided by plasmon resonance scattering probing the plasmon resonance energy transfer^36^. Without ES, quantized dips from scattering spectra were obtained by subtracting the spectra for the unmodified particle and vice versa. For GNP100@r.Cyt *c*@Z samples (no ES), the scattering peaks were observed at ~465 nm (ITO substrate); a broad peak due to r.Cyt *c* and Z, at around 568 nm; and a localized surface plasmon resonance peak (LSPR), shifted to 711 nm (LSPR of GNP100 = 612 nm; Fig. 6c and Supplementary Fig. 38a). This equates to the frequencies with the absorption peak of r.Cyt *c* or Z and subsequently induces the electronic excitation of the r.Cyt *c* at the specific wavelength^8^. Importantly, the QBETs captured are cumulative. Under ES, we see a quantized dip at 536 nm due to the switching of r.Cyt *c* to o.Cyt *c* and thus representing QBET (Fig. 6d). We also see a 20 mV shift in the surface plasmon resonance peak (heat maps in Extended Data Fig. 2a (no EF) and Extended Data Fig. 2b (EF applied)) indicative of charge transfer, which we associate with the redox behavor of an electron relay donor complex^37,38^. The appearance of o.Cyt *c* was not observed in all other control samples with ES (3 MHz, 0.65 V; Supplementary Fig. 38b–i). The combined data indicate that resonant frequency effects resulting in cell death, model exponential decay based on barrier junction energetics and quantized dips from plasmon resonance energy transfer all represent electron tunnelling. Linker length, applied frequency and voltage were found to be critical for QBET-mediated electrical–molecular communication and inducing cancer cell apoptosis. A detailed discussion on the role of these parameters is in Supplementary Note 5. Collectively and to the best of our knowledge, this is the first successful demonstration of electrical–molecular quantum signalling technology in biology, which could be used for the induced apoptosis of cancer cells as a biomedical exemplar.

## Conclusions

Inspired by wireless electrochemistry, we tuned the redox state of molecules functionalized on the surface of nanoantennae by applying a.c. EFs. This approach is shown to selectively facilitate electrical–molecular communication to induce apoptosis in patient-derived GBM cells by switching the redox state of Cyt *c* at a resonant electric field. Based on the obtained data, we infer that the electron transfer in the bio-nanoantennae occurs through EF-induced quantum tunnelling and is thus QBET, with the applied frequency, potential and linker length playing a critical role. Moreover, transcriptomics shows that electrical–molecular communication is specifically targeted in cancer cells. This represents a wireless electrical–molecular communication tool that facilitates the killing of cancer cells.

## Methods

### Reagents

The following reagents were used: horse heart Cyt *c* (C7752, Sigma-Aldrich), EDC (E6383, Sigma-Aldrich), NHS (130672, Sigma-Aldrich), 2-(*N*-morpholino)ethane sulfonic acid hydrate, 4-morpholineethanesulfonic acid (M8250, Sigma-Aldrich), l-ascorbic acid (A92902, Sigma-Aldrich), μ-plate 24-well black ibiTreat surface (IB-82426, Thistle Scientific), Hoechst 33342 (NucBlue Live ReadyProbes Reagent, R37605, Thermo Fisher Scientific), actin using Phalloidin–iFluor 488 conjugate (23115, AAT Bioquest, Stratech), PrestoBlue HS cell viability reagent (P50200, Invitrogen), calcein AM (C1430, Thermo Fisher Scientific), propidium iodide (P4170, Sigma-Aldrich), H2DCFDA (D399, Invitrogen), CellEvent Caspase-3/7 Green Flow Cytometry Assay Kit (C10427, Invitrogen), Zombie NIR Fixable Viability Kit (423105, BioLegend), CellEvent Caspase-3/7 Green ReadyProbes Reagent (R37111, Invitrogen), CellLight Late Endosomes-GFP, BacMam 2.0 (C10588, Thermo Fisher Scientific) and LysoTracker Green DND-26 (L7526, Thermo Fisher Scientific). A catalogue number is not available for the carboxylic-PEG-coated GNPs or Z as they were customized and purchased from Nanopartz and Porphychem, respectively.

### Synthesis of bifunctionalized gold bipolar bio-nanoantennae

Spherical GNPs with a diameter (*d*) of 100 nm and capped with thiol-carboxylic-PEG (SH-PEG-COOH; molecular weight *M* = 1 kDa, 2 kDa, 3.5 kDa and 5 kDa) with a PEG density of 1–1.5 nm^2^ were purchased from Nanopartz. The r.Cyt *c* was obtained by adding 10 mg of the oxidized form of the horse heart Cyt *c* (o.Cyt *c*) into 5 mL l-ascorbic acid solution (1 mg mL^–1^ in PBS) and purifying by dialysis at 4 °C in dark conditions for 36 h to remove excess ascorbic acid. The r.Cyt *c* and Z were covalently conjugated to a carboxylic group on the capping ligands of GNPs using EDC/NHS carbodiimide coupling chemistry. Briefly, 20 μL GNP solution (3.6 mg mL^–1^ in ultrapure water) was mixed with 20 μL EDC/NHS solution in 2-(*N*-morpholino)ethane sulfonic acid buffer (10 mM, pH 5.5) at a concentration of 30 and 36 mg mL^–1^. The solution was mixed for 1 h at room temperature; then 1 mL of washing buffer (×1 PBS with 0.01% (w/v) Tween 20) was added, and the solution was centrifuged at 450*g* for 20 min. The supernatant was discarded and 20 μL r.Cyt *c* (1 mg mL^–1^) and Z (0.5 mg mL^–1^) were added to the pellet and sonicated using a Fisherbrand ultrasonic bath (FB11201, 80 KHz, 50% power, 1 min). Next, the solution was incubated for 4 h at room temperature under mixing; then 1 mL washing buffer was added, and the solution was centrifuged at 450*g* for 20 min. To ensure the complete removal of unbound r.Cyt *c* and Z, the washing step was repeated twice. The obtained pellet consist of GNPs conjugated with r.Cyt *c* and Z (GNP100@r.Cyt *c*@Z) was dispersed in PBS and stored at 4 °C until further use.

To prepare the control samples of GNPs covalently conjugated with only one molecule, either r.Cyt *c* (GNP100@r.Cyt *c*) or Z (GNP100@Z), only one of these compounds was introduced during the EDC/NHS step with concentrations of 0.25 mg mL^–1^ or 0.1 mg mL^–1^, respectively. The same protocol was used to synthesize bio-nanoantennae with different GNP diameters (20 nm, 50 nm and 100 nm) and different PEG lengths (1 kDa, 2 kDa, 3.5 kDa, 5 kDa). The concentration of Cyt *c* and Z during the EDC/NHS step was optimized (0.25, 0.5 and 1 mg mL^–1^) to obtain a similar binding ratio of Cyt *c* and Z per GNP.

### Characterization techniques

#### Dynamic light scattering and zeta potential measurements

The hydrodynamic diameter (*h*_d_) and zeta potential (*ζ*) of bio-nanoantennae (in ultrapure water) was measured using a Malvern Zetasizer Nano-ZS ((Malvern Instruments).

#### Transmission electron microscopy

A TEM instrument (JEOL 2000 FX TEM) operating at 200 kV accelerating voltage was used to record TEM images. The samples were prepared by drop-casting 10 μL of sample onto a carbon-coated copper grid (400 Mesh, Agar Scientific) twice in an interval of 1 h. The sample was dried for at least 30 min before TEM imaging.

#### UV–visible absorption spectroscopy

UV–visible absorption spectra of bio-nanoantennae dispersed in PBS were recorded on a Cary 3500 UV–visible instrument (Agilent Technologies).

#### Circular dichroism

Far- and near-UV circular dichroism spectra were recorded at 20 °C on a Chirascan circular dichroism spectrophotometer (Applied Photophysics) equipped with a temperature control unit TC125 (Quantum Northwest). Samples were dispersed in 10 mM PBS at pH 7.4. At least three spectra were recorded for each sample and averaged. A quartz cuvette with an optical path length of 1 cm was used for the circular dichroism measurements.

#### Cyclic voltammetry

The electrochemical analyses were conducted using a Metrohm Autolab M204 potentiostat equipped with a three-electrode system consisting of a platinum wire counter electrode, an Ag/AgCl reference electrode and an ITO working electrode (Delta Technologies). ITO-coated glass (10 mm × 20 mm) was washed with acetone and water, dried with argon and assembled into an electrochemical cell with an exposed working area of 38.5 mm^2^. Bifunctionalized gold bipolar nanoelectrodes were dispersed in PBS to a final concentration of 25 μg mL^–1^ (determined using UV–visible spectroscopy). Cyclic voltammetry was conducted between 1.2 V and −0.2 V with varying scan rates between 50 mV s^−1^ and 2 V s^−1^. Repetitive consecutive cyclic voltammetry measurements were conducted at a fixed scan rate of 100 mV s^−1^. Control cyclic voltammetry measurements were conducted with carboxylic-PEG-modified GNPs using PBS as the supporting electrolyte.

The heterogeneous rate constant (*k*^0^) was calculated using the Nicholson and Shain method^46^. The rate transfer coefficient, *α*, was calculated from the scan rate study (Supplementary Fig. 5). The slope of the logarithm of the scan rate versus the difference between the peak potential and formal potential of the cell is given by equation (2):

2 $${\rm{Slope}}=-\frac{2.3\,{RT}}{\alpha {nF}}$$

where *R* is the gas constant, *T* is temperature, *n* is the number of electrons transferred in the redox reaction and *F* is the Faraday constant.

However, the Nicholson and Shain method for determining *ψ* (*ψ* is a function of the peak separation) assumes that *α* = 0.5. But in this work, the value of *α* is different (Supplementary Table 5); therefore, the Lavagnini method^47^ is used to calculate *ψ* as the function of the peak separation Δ*E*_p_ using equation (3):

3 $$\psi =2.18{\left(\frac{\alpha }{\uppi }\right)}^{0.5}{{\exp }}\left[\right.-\left(\frac{{\alpha }^{2}F}{{RT}}\right)n\Delta {E}_{\mathrm{{p}}}.$$

Then *k*^0^ for the nanoantennae is calculated using equation (4):

4 $$\psi ={k}^{0}{\left[\frac{{{\uppi }}{{D}}{nv}{{F}}}{{{RT}}}\right]}^{-0.5}$$

where *D* is the diffusion coefficient.

### Cell lines

GIN cells were isolated from the 5-aminolevulinic acid (5-ALA) fluorescing infiltrative tumour margin, and GCE cells were isolated from the core central region of the tumour, from GBM patients who underwent surgery at the Queen’s Medical Centre, University of Nottingham (Nottingham, UK), using a previously described method^29^. Low-passage U251 cell lines (purchased from ATCC) and patient-derived GIN 28, GIN 31, GCE 28 and GCE 31 cells were cultured in DMEM medium (Gibco) supplemented with 10% foetal bovine serum (FBS), 1% penicillin/streptomycin and 1% l-glutamine. Human-derived cortical astrocytes (HCOA; catalogue no. 1800, batch no. 24490, ScienCell) and cerebellar astrocytes (HCEA; catalogue no. 1810, ScienCell) were cultured in astrocyte medium containing 2% FBS, 1% astrocyte growth supplement and 1% penicillin/streptomycin from ScienCell. The human intrahepatic biliary epithelial cells (HIBEpiC) isolated from healthy human liver tissue were acquired from Innoprot (P10654) and cultured in epithelial basal medium containing 2% FBS, 1% epithelial cell growth supplement and 1% penicillin/streptomycin. All cells were maintained at 37 °C in an incubator with a humidified atmosphere, containing 5% CO_2_. Cells were routinely tested for mycoplasma (once a month), and they were grown in an antibiotic-free medium for one week before mycoplasma testing. All cells used were mycoplasma-free.

### PrestoBlue HS assay for biocompatibility and metabolic activity studies

The cells (U251, HCOA, GIN and GCE) were seeded in a 96-well plate at a density of 5 × 10^3^ cells per well and allowed to adhere for 24 h. The medium was replaced with fresh medium containing GNP conjugates at different concentrations (25, 50 and 100 μg mL^–1^), and the cells were incubated for 8 h. The medium was removed, and the cells were washed with PBS and incubated for another 48 h in fresh medium. The medium was replaced with a complete medium containing 10% PrestoBlue HS cell viability reagent and incubated for an hour before reading the fluorescence at 590 nm and 610 nm (excitation and emission) in a Tecan microplate reader (Infinite M Plex and Spark 10M). Cells grown in culture media provided only the negative control. Values are presented relative to the negative control. The data are represented as an average of a triplicate experiment with three independent repeats.

### Cellular association using ICP-MS and uptake using confocal microscopy

GIN and GCE cells were seeded into a 24-well plate at a density of 1 × 10^5^ cells per well and incubated at 37 °C for 24 h. After 24 h, the culture medium was replaced with fresh medium containing 25 μg mL^–1^ of GNP100@r.Cyt *c*@Z and incubated for 8 h. Then the medium was removed, and the cells were washed with PBS (300 μL, repeated two times). The cells were trypsinized, and 50 μL of cell suspension was used for trypan blue cell viability and counting. The remaining cell suspension was centrifuged at 300*g* for 5 min. The obtained cell pellet was digested overnight with 70% nitric acid, diluted with Milli-Q water to bring the acid concentration to 2% and used for ICP-MS analysis (iCAPQ, Thermo Fischer).

To confirm the cellular uptake of the bipolar nanoelectrode, after 8 h exposure, the cells were washed with PBS (300 μL, two times), fixed with 4% paraformaldehyde for 15 min and washed twice with PBS. The cell nuclei were stained with Hoechst 33342 and actin using Phalloidin–iFluor 488 conjugate and incubated for 1 h at 37 °C in the dark. After washing the cells twice and immersing them in PBS, the fluorescence imaging was performed using a Leica TCS SPE confocal microscope. The orthogonal sections of *z* stacks were obtained, and the images were analysed using ImageJ.

### Electrical stimulation studies

U251, HIBEpiC, GIN 28, GIN 31, GCE 28 and GCE 31 cells were seeded in a 24-well plate (μ-plate 24-well black ibiTreat, Thistle Scientific) at a density of 7.5 × 10^4^ cells per well, while HCOA and HCEA were seeded at a density of 5 × 10^4^ cells per well in a poly-l-lysine-coated 24-well plate. The cells were incubated for 24 h at 37 °C and 5% CO_2_, and then the cell culture medium was replaced with fresh medium containing bio-nanoantennae (25 μg mL^–1^) and incubated for 8 h. The cells were washed twice with PBS. Two steel electrodes (0.5 mm × 25 mm) were placed at a fixed distance (at opposite sides of the well and 10 mm from each other) in each well of a 24-well plate and dipped in cell culture medium. The electrodes were connected to an arbitrary function generator (AFG-21225, RS PRO) to deliver the required a.c. sine-wave signals, frequency and amplitude. The cells were stimulated with a.c. EFs with a frequency of 3 MHz and a peak voltage amplitude of 0.65 V cm^–1^ for a period of 2 h or 12 h. The EF between the electrodes was measured using a digital oscilloscope (TDS 210, Tektronix), and the temperature was monitored every 2 h using an infrared laser gun (IR-801, ATP). The intensity of the EF is expressed in peak voltage amplitude per centimetre (V cm^–1^). The metabolic activity of cells after ES was analyzed using a PrestoBlue HS assay.

### Calcein AM and propidium iodide live–dead assay

Immediately after the ES, the medium was removed and replaced with fresh medium containing mixed dyes 1 μM calcein AM and 1 μg mL^−1^ propidium iodide, and incubated for 30 min at 37 °C and 5% CO_2_. The cells were washed twice with PBS, and fresh phenol red free medium was added. The cells were imaged using a Nikon Eclipse Ti fluorescent microscope with GFP and mCherry filter settings. The populations of live and dead cells were quantified using ImageJ software.

### H_2_DCFDA/DCF reactive oxygen species generation assay

The cells were incubated with non-fluorescent cell-permeant 2′,7′-dichlorodihydrofluorescein diacetate (H_2_DCFDA, 5 μM) probe for 30 min prior to ES. Immediately after the ES, the cells were washed with PBS. The generated reactive oxygen species converted H_2_DCFDA into 2′,7′-dichlorofluorescein. Green fluorescence of 2′,7′-dichlorofluorescein was detected using a Nikon Eclipse Ti with FITC filter settings.

### Caspase 3/7 flow cytometry analysis of cell death

Immediately after the ES, cells were trypsinized and centrifuged (300*g* for 5 min) to obtain a cell pellet. After washing with PBS, cells were incubated with a dye master mix containing CellEvent Caspase-3/7 Green Detection Reagent (1:1,000) and Zombie NIR fixable viability stain (1:2,500) for 30 min. Then the cells were centrifuged at 300*g* for 5 min, washed with PBS and fixed with 4% paraformaldehyde. The fluorescence signals of the caspase 3/7 dye (excitation and emission, 511 nm and 523 nm) and Zombie NIR dye (excitation and emission, 719 nm and 746 nm), characteristic for apoptotic and necrotic cell populations, respectively, were detected using a Sony ID7000 spectral flow cytometer. Kaluza software (v.2.1) was used to analyze the data.

### Caspase 3/7 detection using confocal microscope

Immediately after the ES, the medium was removed and the cells were incubated with 8 µM CellEvent Caspase-3/7 Green ReadyProbes Reagent in PBS containing 5% FBS for 30 min at 37 °C. Afterwards, the cells were fixed with 4% paraformaldehyde for 20 min and subsequently washed twice with PBS. Later the cells were treated with actin stain Phalloidin–iFluor 594 conjugate for 90 min at 37 °C in the dark and washed again with PBS, followed by staining with Hoechst 33342 for 10 min. Finally, the cells were washed with PBS (twice) and imaged using a Leica TCS SPE confocal microscope with a ×63 objective using the filter settings of Alexa Fluor 488 and Texas Red 594 dyes.

### Colocalization studies

GIN and GCE cells were seeded at a density of 4 × 10^4^ cells per well in a 24-well plate and incubated at 37 °C for 24 h. After 24 h, the culture medium was replaced with fresh medium containing CellLight Late Endosomes-GFP, BacMam 2.0 and incubated overnight at 37 °C and 5% CO_2_. Later, the medium was replaced with fresh medium containing 25 μg mL^–1^ of GNP100@r.Cyt *c*@Z and incubated for 8 h. Immediately after the ES, the cells were washed with PBS and imaged using a Leica confocal microscope. For lysosomal staining, immediately after ES, the cells were incubated with medium containing 100 nM LysoTracker Green DND-26 for 30 min followed by washing and imaging.

### Gene regulation analysis

#### Differential gene regulation analysis

Immediately after the ES (3 MHz, 0.65 V cm^–1^, 2 hours) cells were washed with PBS, trypsinized and centrifuged to obtain a pellet. The cell pellets were snap-frozen in liquid nitrogen for 5 min and stored at −80 °C until shipment (in dry ice) to the Qiagen genomics facility at Hilden, Germany.

#### Sample preparation

RNA was isolated from 200,000 cells using the RNeasy Micro (Qiagen) according to the manufacturer’s instructions with an elution volume of 14 µL.

#### Library preparation and sequencing

The library preparation was done using the QIAseq UPX 3′ Transcriptome Kit (Qiagen). A total of 10 ng purified RNA was converted into cDNA Next Generation Sequencing (NGS libraries). During reverse transcription, each cell is tagged with a unique ID and each RNA molecule is tagged with a unique molecular index (UMI). Then the RNA is converted to cDNA (complementary Deoxyribonucleic acid). The cDNA was amplified, the PCR (polymerase chain reaction) indices were added and the libraries were purified. Library preparation was quality controlled using capillary electrophoresis (Agilent DNA 7500 Chip). Based on the quality of the inserts and the concentration measurements, the libraries were pooled in equimolar ratios. The library pool(s) were quantified using qPCR (quantitative polymerase chain reaction). Each library pool was then sequenced on a NextSeq (Illumina) sequencing instrument according to the manufacturer instructions with 100 bp read length for read 1 and 27 bp for read2. Raw data were de-multiplexed, and FASTQ files for each sample were generated using the bcl2fastq2 software (Illumina).

#### Read demultiplexing, mapping and quantification of gene expression

The ‘Demultiplex QIAseq UPX 3′ reads’ tool of the CLC Genomics Workbench v.20.0.4 was used to de-multiplex the raw sequencing reads according to the sample indices. The ‘Quantify QIAseq UPX 3′ workflow’ was used to process the de-multiplexed sequencing reads with default settings. In short, the reads are annotated with their UMI and are then trimmed for poly(A) and adapter sequences, minimum reads length (15 nucleotides), read quality and ambiguous nucleotides (maximum of 2). They are then deduplicated using their UMI. Reads are grouped into UMI groups when they (1) start at the same position based on the end of the read to which the UMI is ligated (that is, Read2 for paired data), (2) are from the same strand and (3) have identical UMIs. Groups that contain only one read (singletons) are merged into non-singleton groups if the singleton’s UMI can be converted to a UMI of a non-singleton group by introducing an single nucleotide polymorphism (the biggest group is chosen). The reads were then mapped to the human genome hg38 and annotated using the refseq GRCh38.p13 mRNA (messenger Ribonucelic acid) annotation.

The ‘Empirical analysis of DGE’ algorithm of the CLC Genomics Workbench v.21.0.4 was used for differential expression analysis with default settings. It is an implementation of the ‘Exact Test’ for two-group comparisons developed by Robinson and Smyth^48^ and incorporated in the EdgeR Bioconductor Package (Robinson et al., 2010)^49^.

For all unsupervised analysis, only genes were considered with at least ten counts summed over all samples. A variance stabilizing transformation was performed on the raw count matrix using the function vst of the R package DESeq2 v.1.28.1. Some 500 genes with the highest variance were used for the principal component analysis. The variance was calculated agnostically to the predefined groups (blind = TRUE). Some 35 genes with the highest variance across samples were selected for hierarchical clustering.

#### Differential gene expression and gene-set enrichment analysis

To identify differentially expressed genes, we used the linear modelling-based limma algorithm on the transcriptome dataset^50^. Briefly, we compared differential mRNA expression among the different conditions (treated versus untreated) across the cell lines. The significantly regulated genes were selected with an adjusted *P* value below 0.05 using the Benjamini–Hochberg correction method for multiple testing. Enrichment analysis of the biological processes was carried out by a gene-set enrichment analysis algorithm^51^. Briefly, the gene sets were obtained from MSigDB (ref. ^52^), and enrichment analyses were conducted among the different conditions across cell lines. Normalized enrichment scores, *P* values and adjusted *P* values (calculated with a standard Benjamini–Hochberg procedure) were retrieved for each of the gene sets. The gene sets with higher normalized enrichment score values and an adjusted *P* value < 0.05 were considered as enriched for a specific GBM region.

To identify the cell-line-specific responses to treatment, the top differentially regulated pathways (*P* value < 0.05) with high normalized enrichment scores across the astrocytic and GBM-derived cell lines were selected.

### Mathematical model of metabolic activity, charging rate and quantum tunnelling

We looked to develop a mathematical model to support the characteristic exponential decay connected with quantum mechanics:

5 $$P\propto {\mathrm{{e}}}^{-\alpha r}$$

where *P* is the probability of electron tunnelling; *α* is the inverse localization length, which scales with the energy barrier that an electron must tunnel through; and *r* is the length of the energy barrier. We define an intrinsic rate of cell death (directly proportional to the probability of a given cell dying within a given time frame), *r*_d_, which we assume is proportional to the rate of cytochrome charging. Then, the number of dead cells (*D*) at time *t* is

6 $$D\left(t\right)={\int }_{\!\!0}^{t}{\rm{d}}{t}^{{\prime} }A\left({t}^{{\prime} }\right){r}_{\mathrm{{d}}}$$

where *A* is the number of alive cells. The metabolic activity is given by

7 $$M\left(t\right)=\frac{A(t)}{A(t)+D(t)}=\frac{T-{r}_{\mathrm{{d}}}{\int }_{\!\!0}^{t}{\rm{d}}{t}^{{\prime} }A\left({t}^{{\prime} }\right)}{T}$$

where *T* = *D* + *A* is the total number of cells. Solving the above equation, we find that $M\left(t\right)={{\mathrm{e}}}^{-{r}_{{\mathrm{d}}}t}.$ therefore, the rate of charging at any given time (*t*) is given by *r*_d_ ∝ –ln[*M*(*t*)].

### Dark-field microscopy and plasmon resonance scattering spectroscopy

The plasmon resonance scattering measurements were carried out on an inverted dark-field microscope (eclipse Ti-U, Nikon) using a ×40 objective lens (numerical aperture, 0.6) and a dark-field condenser (0.8 < numerical aperture < 0.95). A halogen lamp (100 W) was used as a source of white light to generate plasmon resonance scattering light. The dark-field images were captured by a true-colour digital camera (Nikon DS-fi). The light scattered from the bifunctionalized nanoantennae was split by a monochromator (grating density, 300 lines mm^–1^; blazed wavelength, 500 nm; Acton SP2300i, Princeton Instruments). An IsoPlane-320 spectrometer was used, and the split light was collected by a charge-coupled device (Pixis 100BX, Princeton Instruments). An a.c. EF of 3 MHz at 0.65 V was applied for 10 min, and scattering spectra were monitored (1,000 frames recorded). The exposure time was 500 ms. The samples for plasmon resonance scattering were prepared by immobilizing nanoantennae on ITO. First, ITO slides were treatment with ethanol, acetone and water under sonication. Next, 50 µl nanoantennae solution was drop-casted on the ITO slides for 10 min, followed by a single-step washing and rinsing with water. Finally, the slides were dried with N_2_ gas.

### Statistics and reproducibility

All the statistical analyses were performed using GraphPad Prism v.9.4.1 software (GraphPad Software). All the data are expressed as mean ± s.e.m., unless specified. For responses that were affected by two variables, a two-way ANOVA with a Tukey post-test was used. The value *P* ≤ 0.05 was considered significant. The number of technical replicates and independent repeats is included in the figure legends.

### Reporting summary

Further information on research design is available in the Nature Portfolio Reporting Summary linked to this article.

## Online content

Any methods, additional references, Nature Portfolio reporting summaries, source data, extended data, supplementary information, acknowledgements, peer review information; details of author contributions and competing interests; and statements of data and code availability are available at 10.1038/s41565-023-01496-y.

### Supplementary information

Supplementary Information Supplementary Figs. 1–38, Notes 1–5 and Tables 1–7.

Reporting Summary

### Source data

Source Data Fig. 2 Source data associated with UV–visible measurements of bio-nanoantennae before ES (Fig. 2b,c), cyclic voltammetry scan rate studies (Fig. 2d) and ICP-MS analysis (Fig. 2e).

Source Data Fig. 3 Source data associated with metabolic activity of GIN 31 and GCE 31 cells (Fig. 3a), GIN 28 and GCE 28 cells (Fig. 3b) and cortical astrocytes (Fig. 3c).

Source Data Fig. 4 Source data associated with the differential gene expression shown in Fig. 4.

Source Data Fig. 5 Source data associated with circular dichroism (Fig. 5a), UV–visible measurements of bio-nanoantennae after ES (Fig. 5b,c) and the metabolic activity of GIN 31 cells as a function of different size bio-nanoantennae (20 nm, 50 nm and 100 nm; GNP20@r.Cyt *c*@Z, GNP50@r.Cyt *c*@Z and GNP100@r.Cyt *c*@Z) synthesized using various linker lengths (1, 2, 3.5 and 5 kDa; Fig. 5d–f).

Source Data Fig. 6 Source data associated with mathematical modelling to determine the rate of donor charging, *r*_d_, calculated using the metabolic activity, using equation (1), compared to the PEG linker length, *L*, for GNP20@r.Cyt *c*@Z (Fig. 6a) and GNP100@r.Cyt *c*@Z (Fig. 6b) in GIN 31 cell samples. Source data for the scattering spectra and spectra difference (Fig. 6c) and quantized peaks (Fig. 6d) for QBET obtained for GNP100@r.Cyt *c*@Z bio-nanoantennae.

Source Data Extended Data Fig. 1 Source data associated with gene-set enrichment analysis of gene ontology biological processes shown in Extended Data Fig. 1a–c.

## Data Availability

Source data are provided with this paper. All other data associated with this manuscript (including the Supplementary Information) can be found at 10.17639/nott.7303. The complete dataset of transcriptomics analysis can be found at the Gene Expression Omnibus (GEO) under accession number GSE233380.
