# Supplementary material for: Wireless electrical–molecular quantum signalling for cancer cell apoptosis
Source: Nat Nanotechnol. 2023 Sep 14;19(1):106–14. doi: 10.1038/s41565-023-01496-y (PMC10796273; doi:10.1038/s41565-023-01496-y)
Supplement: Supplementary file 1 — Supplementary Figs. 1–38, Notes 1–5 and Tables 1–7. [file 41565_2023_1496_MOESM1_ESM.pdf]

nature nanotechnology

Article

https://doi.org/10.1038/s41565-023-01496-y

# Wireless electrical–molecular quantum signalling for cancer cell apoptosis

In the format provided by the authors and unedited

## Table-of-contents


1. Supplementary Note/Figures/Tables — Page

| Entry | Page |
|---|---|
| Supplementary note 1: Design of bio-nanoantennae for actuating cell death | 4 |
| Supplementary Fig. 1: DLS of bio-nanoantennae before electrical stimulation (ES) | 5 |
| Supplementary Fig. 2: Zeta potential of bio-nanoantennae before ES | 5 |
| Supplementary Fig. 3: Zoomed in UV-Vis spectrum before ES and its deconvolution to quantitate number of bound molecules | 6 |
| Supplementary Table 1: Calculation of background correction factor from the UV-Vis spectrum of bio-nanoantennae before ES | 7 |
| Supplementary Table 2: Calculating the concentration of r.Cyt *c* and Z bound to single Gold Nanoparticle before ES using Beer-Lamberts law | 7 |
| Supplementary Table 3: Calculating the number of r.Cyt *c* and Z molecules bound to a single Gold Nanoparticle calculated before ES using Beer - Lambert law | 8 |
| Supplementary Table 4: Determination of % coverage by r.Cyt *c* and Z on GNP100@r.Cyt*c*@Z | 8 |
| Supplementary Fig. 4: Cyclic voltammetry scan rate studies | 9 |
| Supplementary Fig. 5: Determination of heterogenous transfer rate coefficient | 10 |
| Supplementary Table 5: The calculated value of formal electrode potential (E°), rate transfer coefficient (α) and heterogenous transfer rate coefficient (k°). | 10 |
| Supplementary Fig. 6: 3D orthogonal Z-stack for bio-nanoantennae uptake in GIN and GCE cells | 11 |
| Supplementary Fig. 7: *In vitro* dose-dependent toxicity of bio-nanoantennae on different cells | 12 |
| Supplementary Fig. 8: ES of GIN 31 cells to determine optimum AC-EF frequency and applied potential for bipolar electrochemistry | 13 |
| Supplementary Fig. 9: Bifunctionalised AC-EFs responsive bio-nanoantennae mediated wireless electrical molecular communication alters the metabolic activity of U251 cells. | 14 |
| Supplementary Table 6: *p* values obtained from the statistical analysis of graphs shown Fig. 3 a-c (main text). | 14 |
| Supplementary Fig. 10: Bifunctionalised AC-EFs responsive bio-nanoantennae mediated wireless electrical molecular communication alters the metabolic activity of GBM cells - 2-hour study. | 15 |
| Supplementary Fig. 11: Bifunctionalised AC-EFs responsive bio-nanoantennae mediated wireless electrical molecular communication do not alter the metabolic activity of normal liver cells and cerebellar astrocytes significantly. | 16 |

| | |
|---|---|
| Supplementary note 2: Discussion on the response of other normal cells (liver and cerebellar astrocyte) to the treatment. | 16 |
| Supplementary Fig. 12: Live/Dead fluorescence microscopy images | 17 |
| Supplementary Fig. 13: Quantification of live and dead cell population | 18 |
| Supplementary Fig. 14: Flow cytometry gating strategy | 19 |
| Supplementary Fig. 15: Representative flow cytometric analysis of GIN 31 cells (control samples) and cortical astrocytes (control and bio-nanoantennae) | 20 |
| Supplementary Fig. 16: Quantification of flow cytometry. | 21 |
| Supplementary Fig. 17: Confocal microscopy images to demonstrate caspase 3/7 activation in cortical astrocytes | 21 |
| Supplementary Fig. 18: Endo-lysosomal escape – confocal imaging. | 22 |
| Supplementary Fig. 19: Analysis of oxidative damage by ROS generation. | 23 |
| Supplementary Fig. 20: AC-EF (3MHz, 0.65V/cm) mediated change in solution temperature monitored over the course of experiment using NIR laser gun. | 23 |
| Supplementary Fig. 21: Transcriptomics analysis - Principal component analysis (PCA) | 24 |
| Supplementary Fig. 22: Transcriptomics analysis - MA plot representing statistical tests of the differential gene expression analyses | 25 |
| Supplementary Fig. 23: Heatmap of Gene-set Enrichment analysis (GSEA) of Gene Ontology Biological Processes (GOBP) | 26 |
| Supplementary Fig. 24: Heatmap of Gene-set Enrichment analysis (GSEA) of Gene Ontology Cellular Components (GOCC) | 27 |
| Supplementary note 3: Discussion of gene ontology and GSEA analysis of GOBP | 28-29 |
| Supplementary Fig. 25: Circular Dichroism (CD) analysis | 30 |
| Supplementary Fig. 26: Deconvolution of UV-Vis absorption spectrum of bio-nanoantennae after electrical stimulation with AC EFs (3 MHz, 0.65 V/cm) | 31 |
| Supplementary Fig. 27: UV-Vis absorption spectrum of bio-nanoantennae without electrical stimulation after 12-hour incubation | 32 |
| Supplementary Fig. 28: DLS of bio-nanoantennae after ES | 32 |
| Supplementary Fig. 29: Zeta potential of bio-nanoantennae after ES | 32 |
| Supplementary note 4: Nanoscale wireless electrochemistry and *in vitro* QBET: Discussion of the data obtained from CD, UV-Vis absorption spectroscopy, DLS, and Zeta potential measurements | 33 |
| Supplementary Fig. 30: TEM images of different size bio-nanoantennae functionalised using 2000 Da thiol-PEG-carboxylic linker. | 34 |
| Supplementary Fig. 31: Size distribution of different size bio-nanoantennae synthesised using linkers of various lengths, analysed using DLS. | 35 |

Supplementary Fig. 32: *In vitro* toxicity of GNP20, GNP50, and GNP100 bio-nanoantennae functionalised using PEG linker of different lengths in the absence of electric fields. 36
Supplementary Table 7: *p* values obtained from statistical analysis of graph shown in Fig. 5d-f (main text) 37
Supplementary Fig. 33: Wireless electrical-molecular quantum signalling: *in vitro* electron tunnelling via bio-nanoantennae for inducing cell death in GCE 31 cells 38
Supplementary Fig. 34: Live/dead staining to demonstrate the effect of linker length and bio-nanoantennae size on GIN 31 killing effect 39
Supplementary Fig. 35: Quantification of live and dead cell population (shown in supplementary figure 33) 39
Supplementary Fig. 36: Number of bio-nanoantennae (different core and linker size) per GIN 31 cell calculated using ICP-MS 40
Supplementary Fig. 37: Ratio of zinc porphyrin (Z) to Cyt *c* per GIN 31 cells determined from number of bio-nanoantennae per GIN 31 cells 40
Supplementary Fig. 38: PRS scattering spectra and quantised peaks of control samples to demonstrated QBET 41
Supplementary note 5: Discussion on the role of that linker length, applied frequency and voltage for QBET mediated electrical-molecular communication and inducing cancer cell apoptosis. 42
2. References 43-44

## 1. Results and Discussions

**Supplementary note 1:** Design of bio-nanoantennae for actuating cell death.

The diameter of GNP100@r.Cyt *c*@Z as analysed by TEM was found to be 105 ± 2 nm (Fig. **2a**). Dynamic Light Scattering (DLS) analysis indicated an average hydrodynamic diameter ($h_d$) increase from 104.9 nm (PEGylated GNPs) to 118.8 nm for the GNP100@r.Cyt *c*@Z, suggesting successful conjugation of rC and Z (Supplementary Fig. **1**). A change in zeta potential (ζ) of GNP100@r.Cyt *c*@Z (-28.5 mV) compared to GNP100 (-27.7 mV), GNP100@r.Cyt *c* (-14.8 mV), and GNP100@Z (-40 mV) suggested the bi-functionalisation process was successful (Supplementary Fig. **2**). UV-Vis absorption spectrum of GNP100@r.Cyt *c*@Z before ES in phosphate buffer saline (PBS) revealed a broad peak centred at 418 nm attributed to overlapping absorption of r.Cyt *c* and Z (Fig. **2 b & c**). This peak was deconvoluted and Marquardt fitting algorithm was applied,[1] which revealed two components centred at 412 nm and 423 nm attributed to r.Cyt *c* and Z (Supplementary Fig. **3 a-b**), respectively, which were corroborated using UV-Vis spectrum of native r.Cyt *c* and Z in PBS (Supplementary Fig. **3c**). The obtained peaks were used for the quantification of r.Cyt *c*, and Z attached to each GNP100@r.Cyt *c*@Z, which revealed homogenous and monolayer conjugation of r.Cyt *c* and Z on GNP100 (Supplementary Fig. **3 d-e** and Table **1-4**). Cyclic voltammetry was carried out to study the redox behaviour of r.Cyt *c* and Z on a bifunctionalised system (Fig. **2d**). Two redox couples were observed in GNP100@r.Cyt *c*@Z, which are attributed to Cyt *c* and Z, while on the other hand, the control samples only showed a redox couple corresponding to either Cyt *c* or Z, which have been characterised by others previously (Supplementary Fig. **4 a-c**).[2,3] The heterogenous electron transfer rate coefficient ($k^0$) of Cyt *c* for GNP100@r.Cyt *c* was calculated to be $9.6 \times 10^{-3}$ cm/s (Supplementary Table **5**), while that for GNP100@r.Cyt *c*@Z was $3.75 \times 10^{-3}$ cm/s (Supplementary Fig. **5 a-f**), suggesting a slight decrease in electron transfer rate of Cyt *c* in a bifunctionalised system.

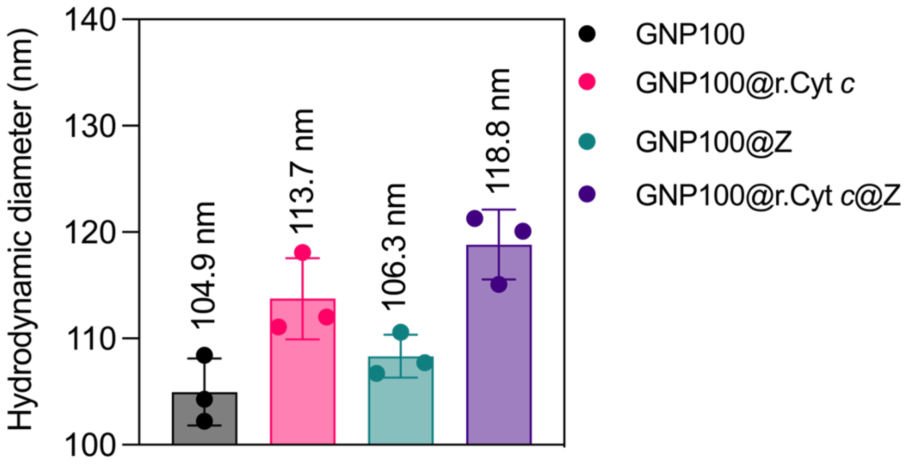


**Figure 1.** Hydrodynamic diameter of bio-nanoantennae (dispersed in ultra-pure water) before ES. Error bars represent mean ± s.d. obtained from 3 individual experiments.

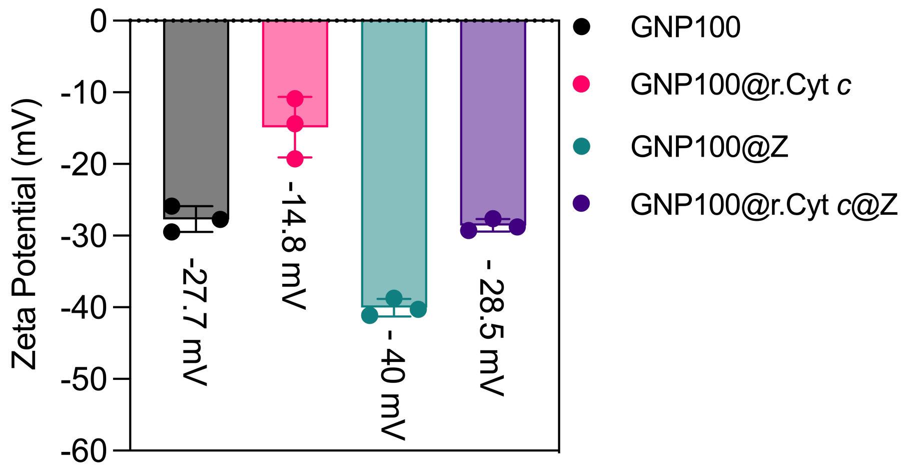


**Figure 2.** Zeta potential of bio-nanoantennae (dispersed in ultra-pure water) before ES. Error bars represent mean ± s.d. obtained from 3 individual experiments.

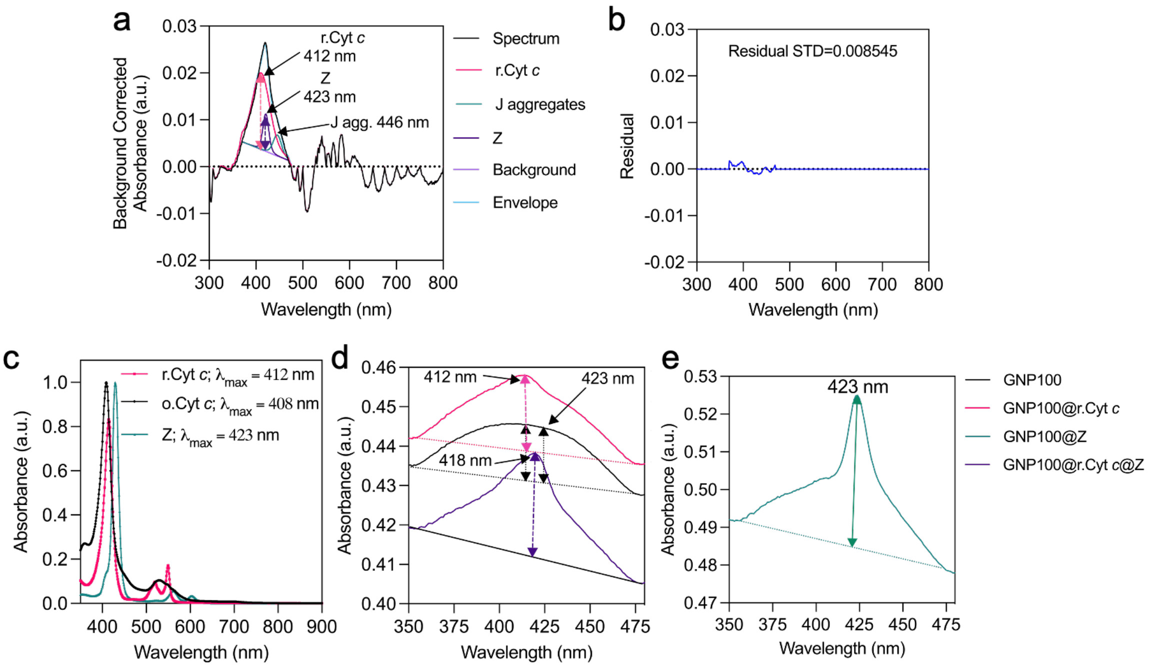


**Figure 3.** UV-vis absorption spectrum of bio-nanoantennae before electrical stimulation. **(a)** Deconvolution and fitting of UV-Vis spectrum of GNP100@r.Cyt *c*@Z to identify and quantitate number of bound r.Cyt *c* and Z molecules. **(b)** Residual standard deviation obtained after curve fitting. **(c)** UV-Vis spectrum of zinc porphyrin (Z), native Cyt *c* in oxidised (o.Cyt *c*) and reduced (r.Cyt *c*) form, in PBS. **(d-e)** Zoomed in UV-Vis spectrum of bio-nanoantennae and baseline correction to quantify number r.Cyt *c* and Z molecules bound to each nanoparticle (Supplementary Table **1-4**).

**Note:** For quantification of r.Cyt *c* and Z on GNPs, firstly a baseline correction factor was calculated by subtracting the absorbance of GNP100 at wavelengths 412 nm (r.Cyt *c*) and 423 nm (Z) to the factor obtained by subtracting actual absorbance with baseline absorbance. Finally, this baseline correction factor was subtracted to obtain the final absorbance of r.Cyt *c* and Z in GNP100@r.Cyt *c*, GNP100@Z, GNP100@r.Cyt *c*@Z samples. Absorbance final ($Abs^f$) was taken to calculate Cyt *c* concentration because of the influence on the spectra of the Cyt *c* due to the SPR peak of GNP.

**Table 1.** Calculating the background correction factor (C.F.) from the UV Vis spectrum of GNP100@r.Cyt *c*@Z before ES.

| | **B.C. r.Cyt *c*** | | **C.F. r.Cyt *c*** | **B.C.Z** | | **C.F.Z** |
|---|---|---|---|---|---|---|
| **Sample** | **Abs λ 412 nm** | **Abs λ = 412 nm baseline** | **r.Cyt *c*** | **Abs λ 423 nm** | **Abs λ = 423 nm baseline** | **Z** |
| GNP100@r.Cyt *c*@Z | 0.445 | 0.431 | 0.014 | 0.445 | 0.42 | 0.025 |

Where,

**B.C.r.Cyt *c*** = Background correction for **r.Cyt *c*; C.F.r.Cyt *c*** = Correction factor for **r.Cyt *c***
**B.C.Z** = Background correction for Z; **C.F.Z** = Correction factor for Z

**Table 2.** Calculating the concentration of r.Cyt *c* and Z bound to single Gold Nanoparticle before ES was calculating using Beer-Lamberts law.

| **Sample** | **r.Cyt *c* .Abs$^a$** | **Z.Abs$^a$** | **r.Cyt *c* .Abs$^b$** | **Z.Abs$^b$** | **r.Cyt *c* Abs$^f$** | **Z Abs$^f$** | **Concentration (M)** |
|---|---|---|---|---|---|---|---|
| GNP100@ r.Cyt *c* | 0.457 | N/A | 0.438 | N/A | 0.005 | N/A | 3 x $10^{-8}$ |
| GNP100@ Z | N/A | 0.523 | N/A | 0.487 | N/A | 0.011 | 1.9 x $10^{-7}$ |
| GNP100@r .Cyt *c*@Z | 0.019 | 0.01 | 0.003 | 0.003 | 0.002 | 0.007 | r.Cyt *c* = 1.3 x $10^{-8}$ Z = 1.2 x $10^{-7}$ |

Where,

**r.Cyt *c*.Abs$^a$** and **Z.Abs$^a$** = Actual absorbance of **r.Cyt *c*** and Z at 412 and 423 nm in Supplementary Fig. **3d**, respectively.

**r.Cyt *c*.Abs$^b$** and **Z.Abs$^b$** = Absorbance at the baseline of r.Cyt *c* and Z.

**r.Cyt *c*.Abs$^f$** = (r.Cyt *c*.Abs$^a$ – r.Cyt *c*.Abs$^b$) – C.F. r.Cyt *c*

**Z.Abs$^f$** = (Z.Abs$^a$ – Z.Abs$^b$) – C.F.Z

**ε ($M^{-1}$ $cm^{-}$)** Z = 57940 and r.Cyt *c* = 1.5 x $10^5$

**Table 3.** Calculating the number of r.Cyt *c* and Z molecules bound to a single Gold Nanoparticle calculated using Beer -Lambert law. Molar extinction coefficient (ε) of PEGylated GNPs (GNP100) was used as given by the supplier (Nanopartz. Inc).

| Sample | Abs[a] SPR band λ = 572 nm | ε of GNP.100 ($M^{-1}$ $cm^{-}$) | Conc. of GNPs (M) | r.Cyt *c* /GNP | Z/GNP |
|---|---|---|---|---|---|
| GNP100@ r.Cyt *c* | 0.972 | 1.21 x $10^{11}$ | 0.8 x $10^{-11}$ | 3750 | N/A |
| GNP100@Z | 1.05 | 1.21 x $10^{11}$ | 0.86 x $10^{-11}$ | N/A | 22906 |
| GNP100@ r.Cyt *c* @Z | 0.92 | 1.21 x $10^{11}$ | 7.6 x $10^{-11}$ | 1710 | 15789 |

**Table 4.** Determination of % coverage by r.Cyt *c* and Z on GNP100@r.Cyt*c*@Z

| Sample | r (nm) | S.A. r.Cyt *c* or S.A.Z ($nm^2$) | S.A. GNP ($nm^2$) | r.Cyt *c*.M.$C^t$ | Z.M. $C^t$ | A. r.Cyt *c*/ GNP ($nm^2$) | A.Z/ GNP ($nm^2$) | Total % coverage |
|---|---|---|---|---|---|---|---|---|
| GNP100@ r.Cyt *c* | 52 | 7.07 | 33979 | 4806 | N/A | 26512 | N/A | 78 |
| GNP100@Z | 52 | 1.53 | 33979 | N/A | 22089 | N/A | 34359 | 103 |
| GNP100@r. Cyt *c*@Z | 52 | 8.6 | 33979 | - | - | 12089 | 23683 | 104 |

**Where,**

**r** = Radius of PEGylated GNP (nm) calculated from DLS analysis.

**S.A.r.Cyt *c* or S.A.Z** = Surface area of a r.Cyt *c* or Z molecule ($nm^2$)

**S.A.GNP** = Surface area of a spherical GNP ($nm^2$)

**r.Cyt *c*.M.$C^t$** = Theoretical maximum coverage of r.Cyt *c* per GNP

**Z.M.$C^t$** = Theoretical maximum coverage of Z per GNP

**A.r.Cyt *c*/GNP** = Area occupied by r.Cyt *c* on a single GNP ($nm^2$)

**A.Z/GNP** = Area occupied by Z on a single GNP ($nm^2$)

**Note:** For calculating the surface area of spherical nanoparticle $h_d$ of PEGylated GNPs (GNP100) = 104 nm, was taken to calculated to radius.

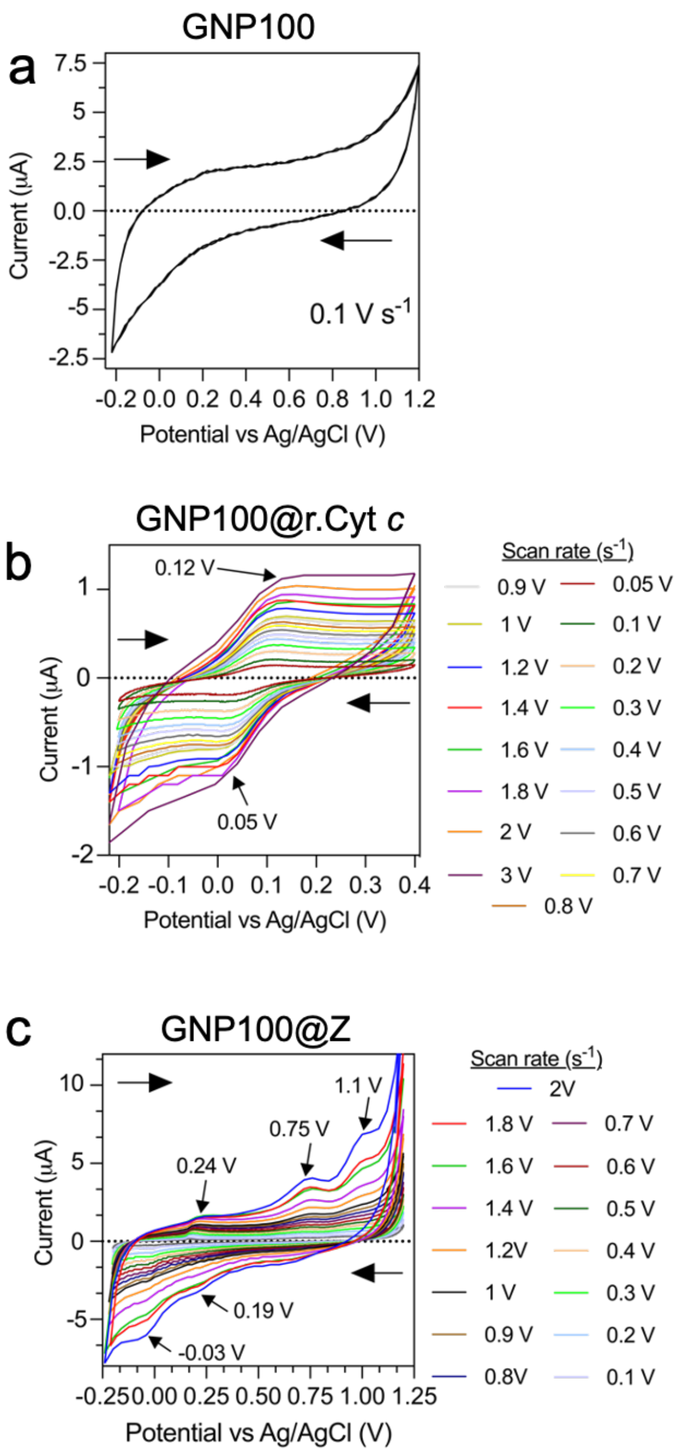


**Figure 4.** Cyclic voltammetry scan rate studies of **(a)** 100 nm GNPs capped with carboxylic-terminated PEG (GNP100), **(b)** GNP100 functionalised with reduced form of cyt *c* (r.Cyt *c*) – GNP100@r.Cyt *c*, and **(c)** Z functionalised GNP100 – GNP100@Z. Indium tin oxide (ITO) was used as a working electrode, counter electrode: platinum wire, and reference electrode: Ag/AgCl, electrolyte: 10 mM PBS. Sample concentration: 25 µg/mL.

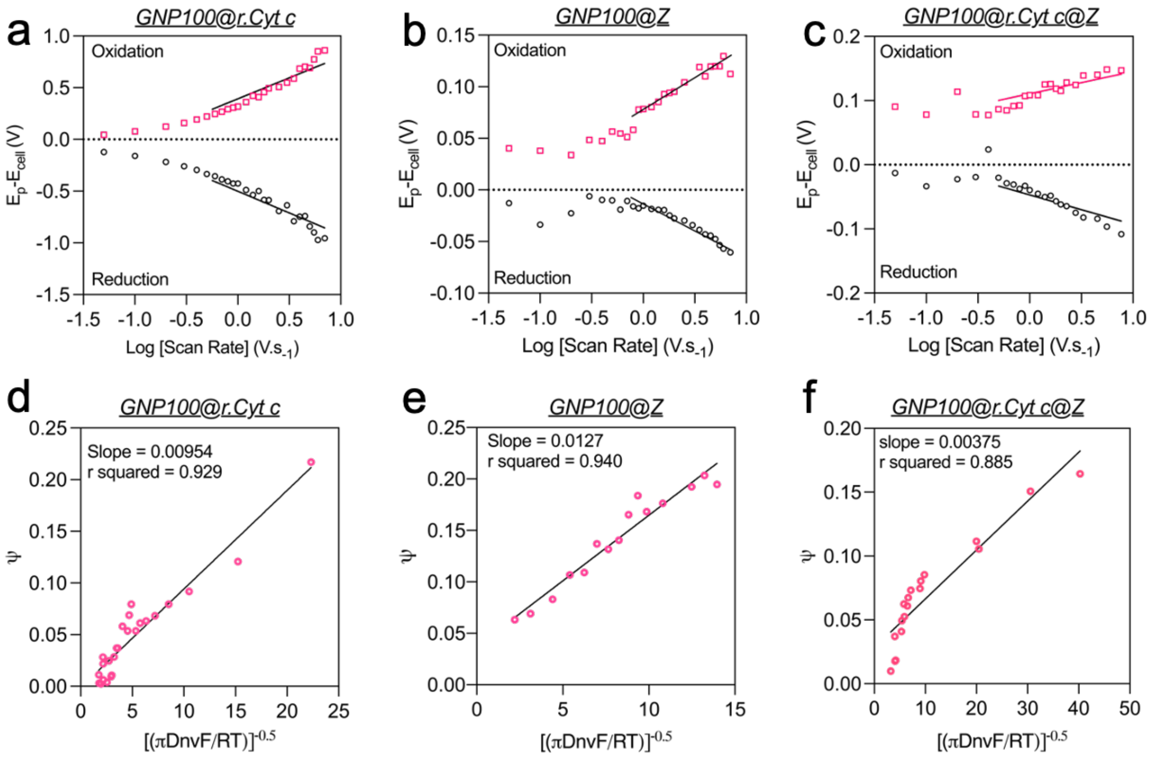


**Figure 5. Determination of heterogenous rate constant (*k*°). (a-c)** Graph of peak potential – Standard Potential *vs* log of scan rate of different nanoantennae to determine rate transfer coefficient. **(d-f)** Nicholson plot to determine *k*° of nanoantennae. N = 3. Scan rates plotted are between 0.1 V/s and 3 V/s.

**Table 5**. The calculated value of formal electrode potential (E°), rate transfer coefficient (α) and heterogenous transfer rate coefficient (k°) using Nicholson and Shain method adapted by Lavagnini *et al.*[4]

| Sample | E° (mV) | α | k° ($cm.s^{-1}$) |
|---|---|---|---|
| GNP100@r.Cyt *c* | 96 ± 4.3 | 0.85 | $9.5 \times 10^{-3}$ |
| GNP100@Z | 73 ± 10.3 | 0.58 | $1.2 \times 10^{-2}$ |
| GNP100@r.Cyt *c*@Z | 87 ± 7.9 | 0.69 | $3.7 \times 10^{-3}$ |

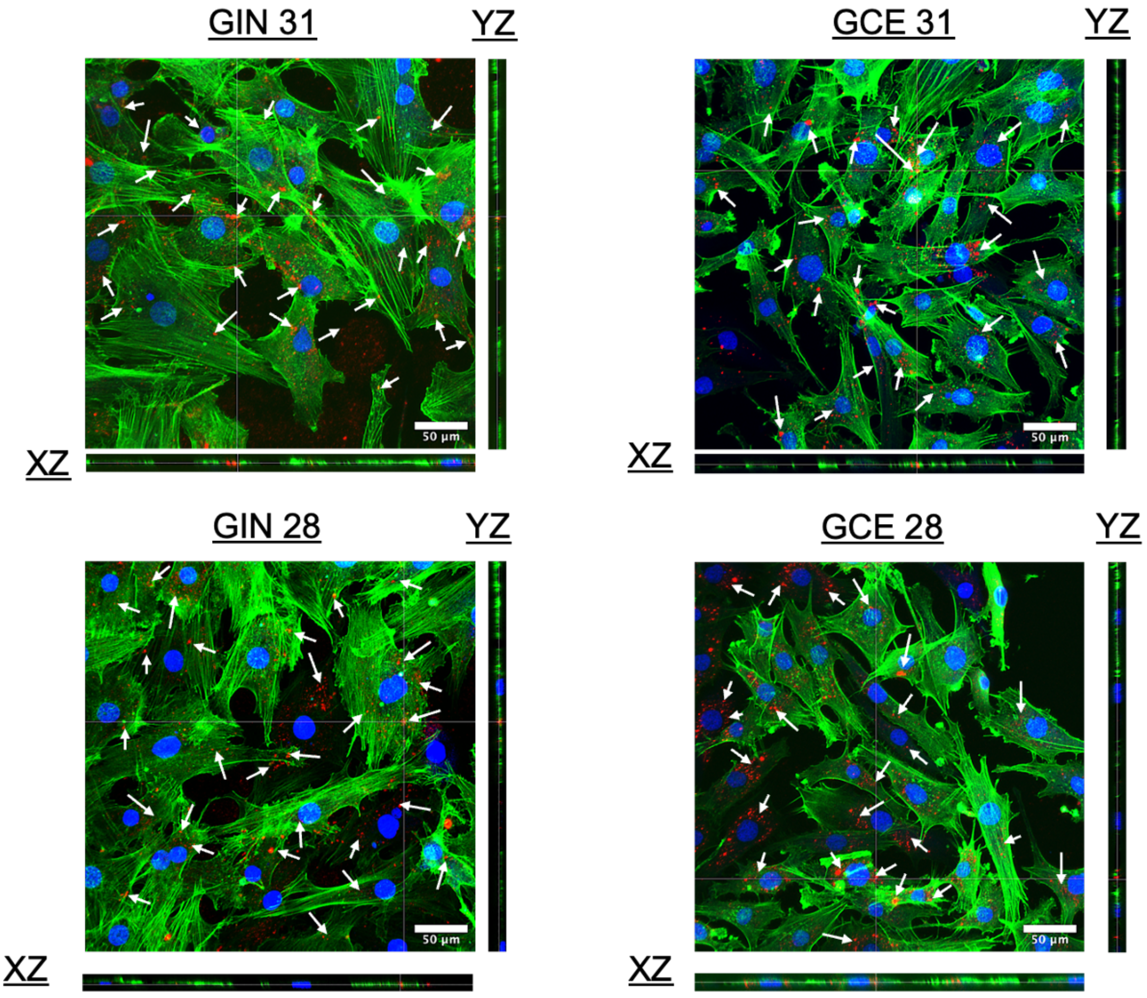


**Figure 6.** 3D orthogonal Z-stack confirming the cytoplasmic localisation of bio-nanoantennae in GCE/GIN 31 and GCE/GIN 28 cells analysed using confocal microscopy. Cells were treated with bio-nanoantennae (GNP100@r.Cyt *c*@Z) for 8 h and then fixed with paraformaldehyde followed by counterstaining with Cytopainter Actin Phalloidin (Alexa 488 green) stain and Hoechst nuclear stain (blue). White arrows indicate bio-nanoantennae (red) in the cytoplasm. To confirm the uptake of bio-nanoantennae at least 50 cells were analysed using Z-stacking.

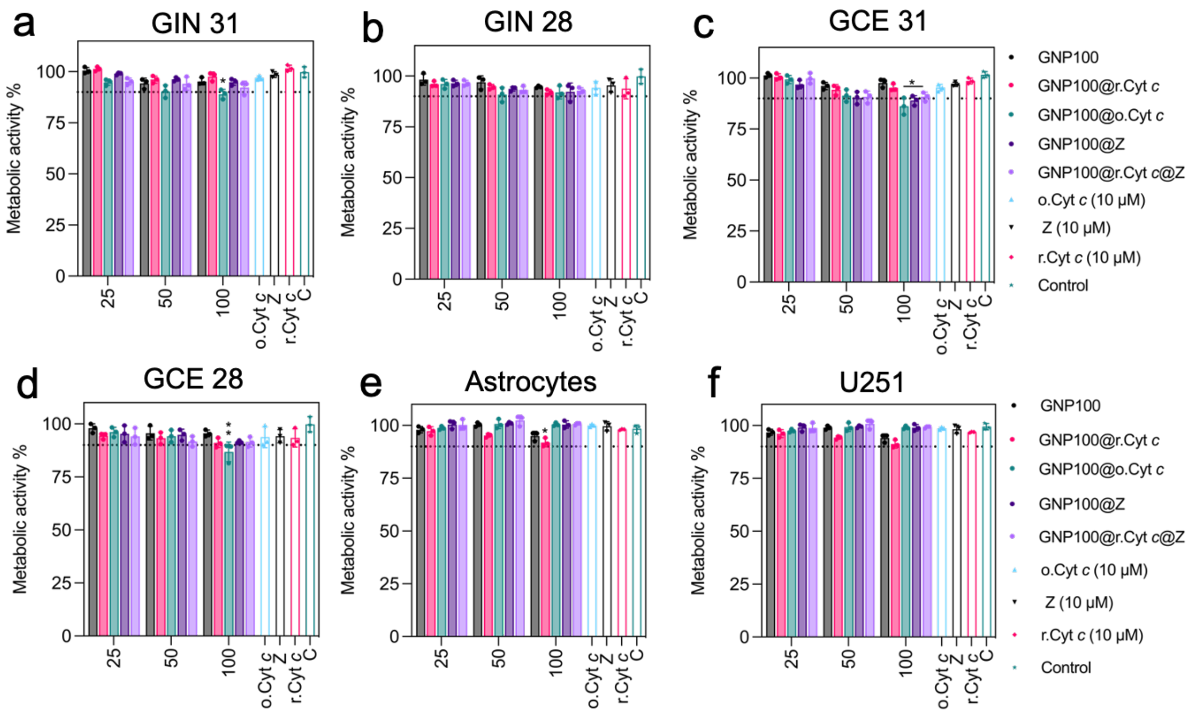


**Figure 7.** ***In vitro*** **dose-dependent toxicity of bio-nanoantennae synthesised using 2KDa PEG Linker. The cells were incubated with different nanocomposites at various concentration for 36 h before analysing their toxicity using PrestoBlue HS assay. (a-f)** Metabolic activity of GIN 31, GIN 28, GCE 31, GCE 28, human derived cortical astrocytes, and U251 glioblastoma cells. GNP100 are 100 nm spherical gold nanoparticles functionalized with 2000 Da (2k) thiol-PEG-carboxylic, GNP100@r.Cyt *c* was obtained by functionalising GNP100 with r.Cyt *c*. Similarly, GNP100@o.Cyt c, GNP100@Z, and GNP100@r.Cyt *c*@Z are GNP100 functionalised with o.Cyt *c*, Z, and r.Cyt *c* & Z, respectively. Error bars represent mean ± standard error of mean (S.E.M.) obtained from triplicate experiments repeated thrice. Statistical analysis was performed by applying 1-way ANOVA with a Dunnett's post-test. *p* value (*vs* control) are as follows: GIN 28 * = 0.047; GCE 31 * = 0.028 (GNP100@o.Cyt *c*); 0.03 (GNP100@Z); 0.035 (GNP100@r.Cyt *c*); GCE 28 ** = 0.0091 and astrocytes * = 0.036.

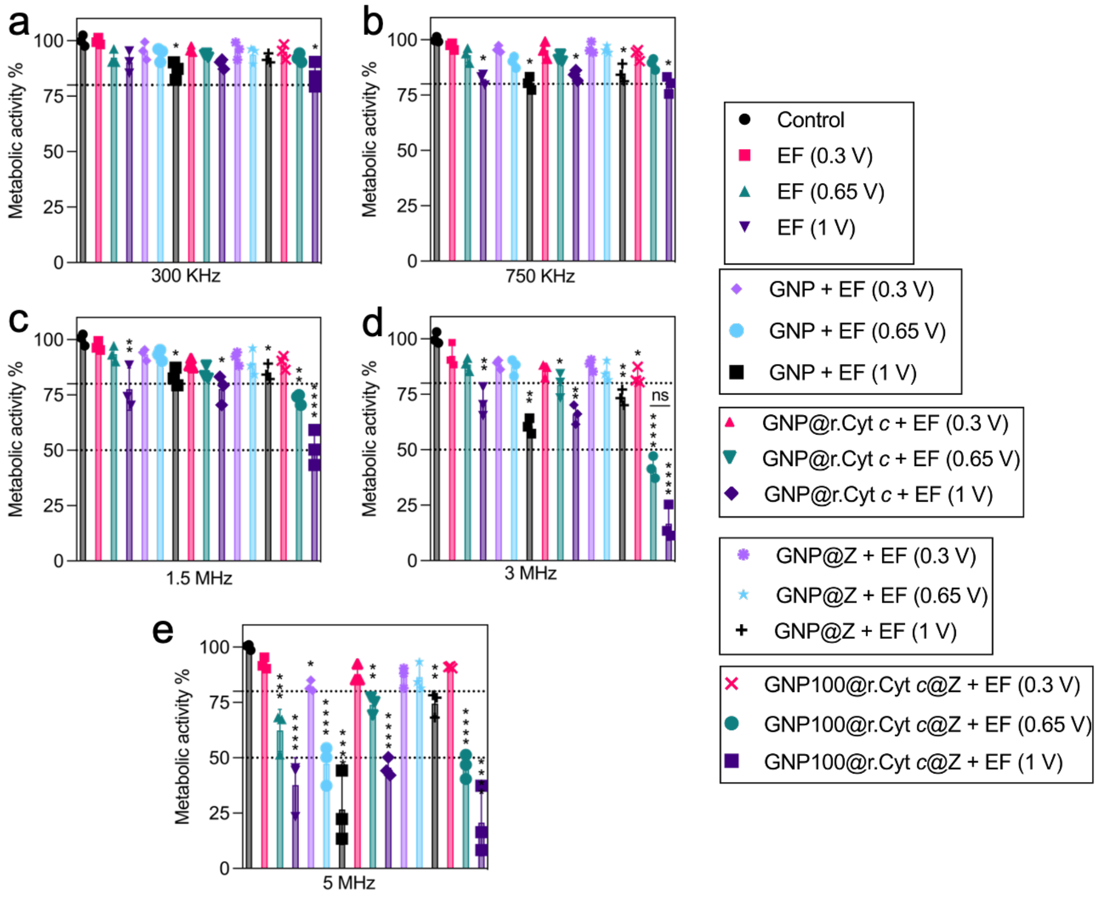


**Figure 8. Electrical stimulation (ES) of GIN 31 cells to determine optimum AC-EF frequency and applied potential for bipolar electrochemistry mediated redox switching and activation of Cyt *c* on the surface of bifunctionalised bio-nanoantennae**. Metabolic activity of GIN 31 cells as a function of AC-EFs calculated using PrestoBlue HS assay. Cells were treated with nanoparticles for 8 h followed by AC-EFs stimulation of different frequency and voltages for 12 h. **(a)** 300 KHz, **(b)** 750 KHz, **(c)** 1.5 MHz, **(d)** 3 MHz, and **(e)** 5 MHz. Error bars represent mean ± standard error of mean (S.E.M.) obtained from triplicate experiments repeated thrice. Statistical analysis was performed by applying 1-way ANOVA with a Dunnett's post-test. The data was considered significant if *$p \leq 0.05$, **$p \leq 0.01$, ***$p \leq 0.001$, and ***$p \leq 0.0001$.

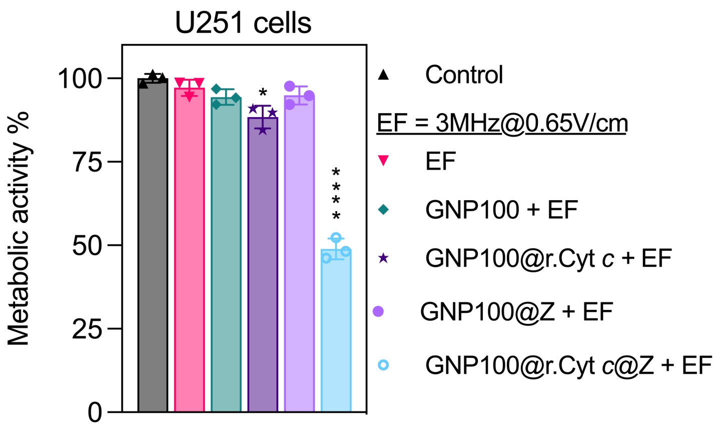


**Figure 9. Bifunctionalised AC-EFs responsive bio-nanoantennae mediated wireless electrical molecular communication alters the metabolic activity of U251 cells.** The cells were treated with GNP100@r.Cyt *c*@Z for 8 h followed by 12 h treatment with AC-EF (3MHz, 0.65V/cm). Error bars represent mean ± standard error of mean (S.E.M.) obtained from triplicate experiments repeated thrice. Statistical analysis was performed by applying 1-way ANOVA. *p* value of * = 0.04 and **** = <0.0001

**Table 6.** *P* values obtained from the statistical analysis of graphs shown Fig. 3 a-c (main text).

| **Cell type** | ***p* value *vs* control (Treatment with GNP100@r.Cyt *c*@Z + EF 12 h)** |
|---|---|
| GIN 31 | 0.00014 |
| GCE 31 | 0.00025 |
| GIN 28 | 0.00019 |
| GCE 28 | 0.0003 |
| Cortical Astrocytes | 0.011 |
| U251 | 0.00016 |

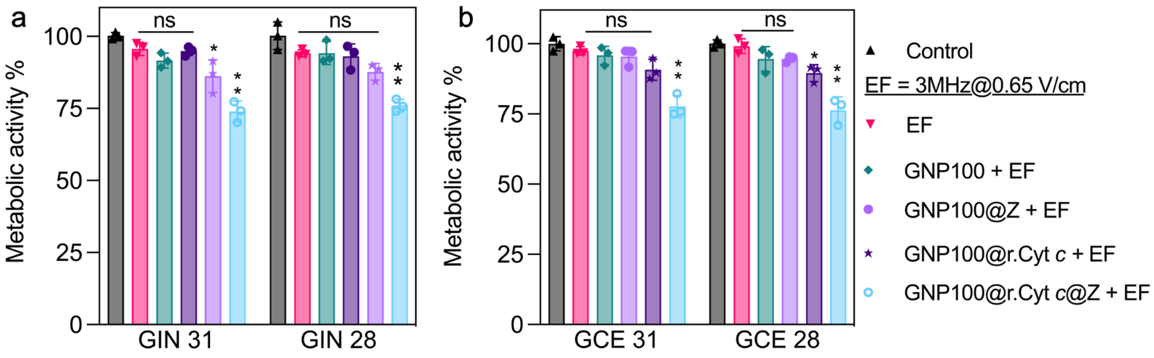


**Figure 10. Bifunctionalised AC-EFs responsive bio-nanoantennae mediated wireless electrical molecular communication alters the metabolic activity of GBM cells.** The cells were treated with GNP100@r.Cyt *c*@Z for 8 h followed by 2 h treatment with AC-EF (3MHz, 0.65V/cm). Metabolic activity of **(a)** GIN (*p* values GIN 31 * = 0.0334; ** = 0.0029 and GIN 28 ** = 0.0054), **(b)** GCE cells (*p* values GCE 31 ** = 0.0022 and GCE 28 * = 0.04; ** = 0.0061). Error bars represent mean ± standard error of mean (S.E.M.) obtained from triplicate experiments repeated thrice. Statistical analysis was performed by applying 2-way ANOVA with Tukey's post-test.

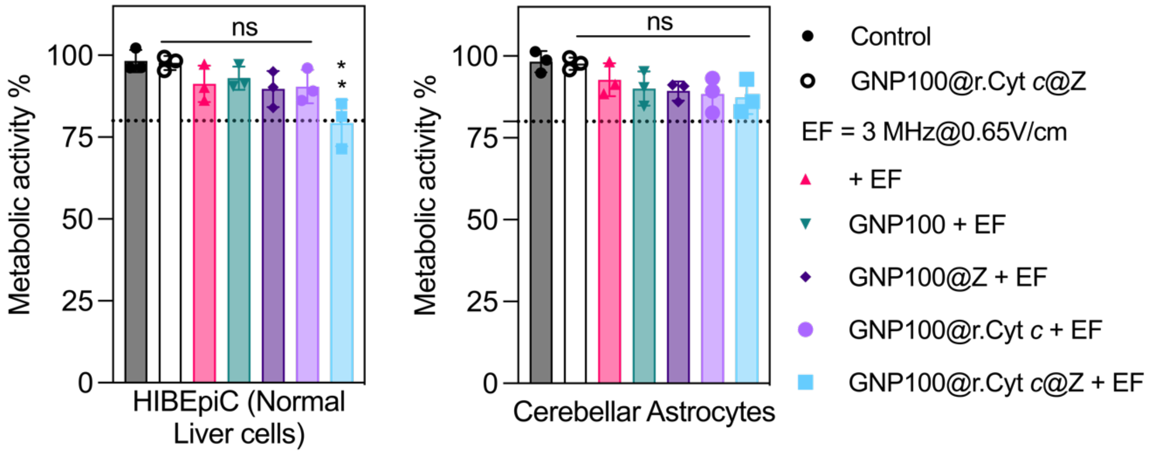


**Figure 11. Bifunctionalised AC-EFs responsive bio-nanoantennae mediated wireless electrical molecular communication do not alter the metabolic activity of normal cells significantly.** The cells were treated with GNP100@r.Cyt *c*@Z for 8 h followed by 12 h treatment with AC-EF (3MHz, 0.65V/cm). Metabolic activity of **(a)** HIBEpiC (isolated from health human liver) where *p* value ** = 0.0028, **(b)** Cerebellar astrocytes (isolated from human cerebellum). Error bars represent mean ± standard error of mean (S.E.M.) obtained from triplicate experiments repeated thrice. Statistical analysis was performed by applying 2-way ANOVA.

**Supplementary note 2:** To further confirm the response of this treatment to GBM cells and not in healthy cortical astrocyte cells, we repeated the experiments using **other normal cells: intrahepatic biliary epithelial cells** (HIBEpiC; isolated from human healthy liver tissue) and cerebellar astrocytes (isolated from human cerebellum). The obtained data suggests a ~22% decrease in HIBEpiC metabolic activity (Supplementary Figs. **11a**, significant *vs* control, *p* value = 0.0016) and a ~15% decrease in cerebellar astrocyte metabolic activity (Supplementary fig. **11b**, non-significant *vs* control, *p* value = 0.33).

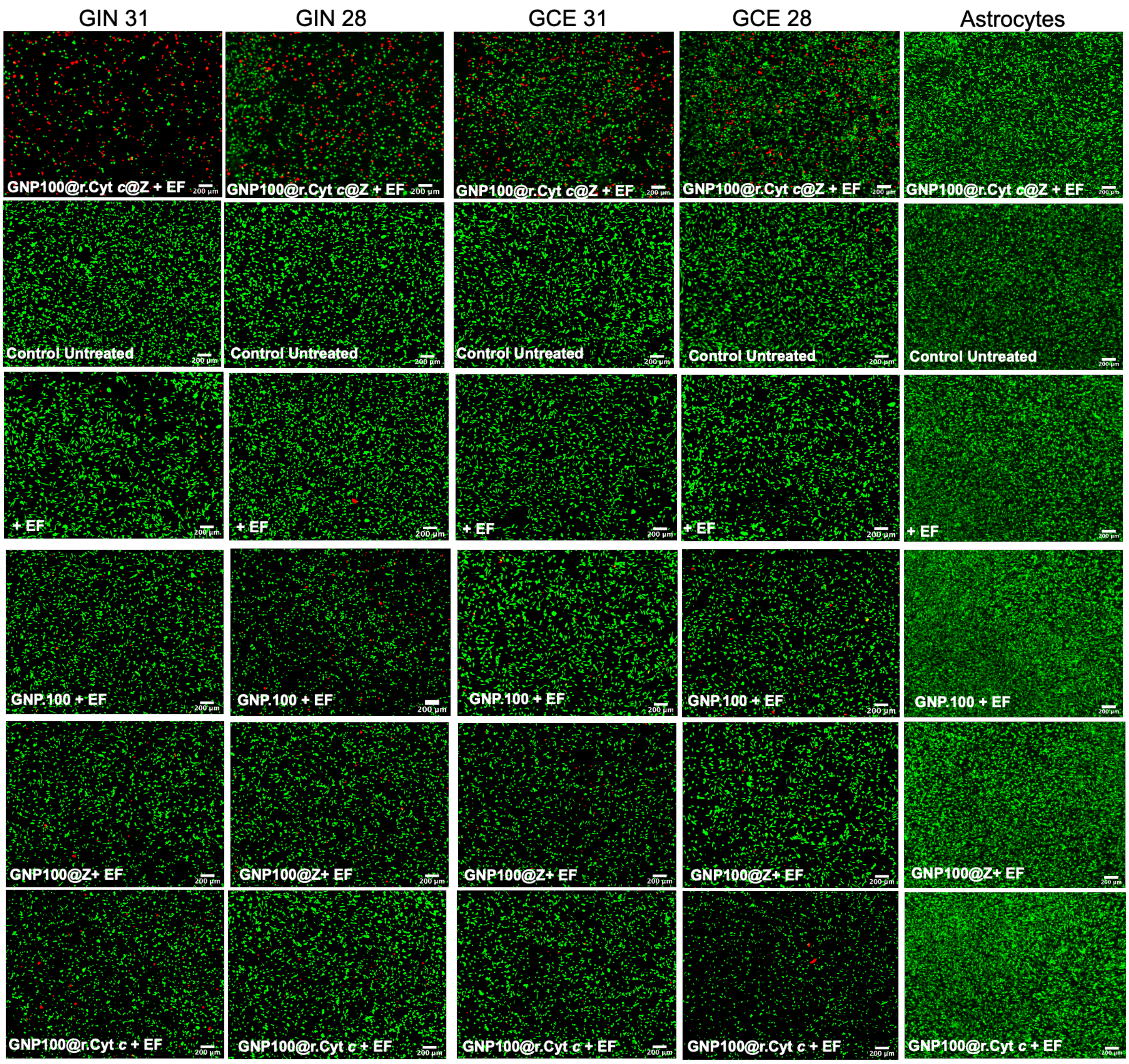


**Figure 12. Live/dead imaging of GIN, GCE, and cortical astrocytes treated with bio-nanoantennae, and other control samples followed by AC EFs treatment for 12-hours.** Post AC-EF treatment the cells were stained with calcein AM (green, live cells) and propidium iodide (red, dead cells). Live and dead cells were imaged using GFP and mTomato channel using a Nikon fluorescent microscope. Scale bar = 200 µm.

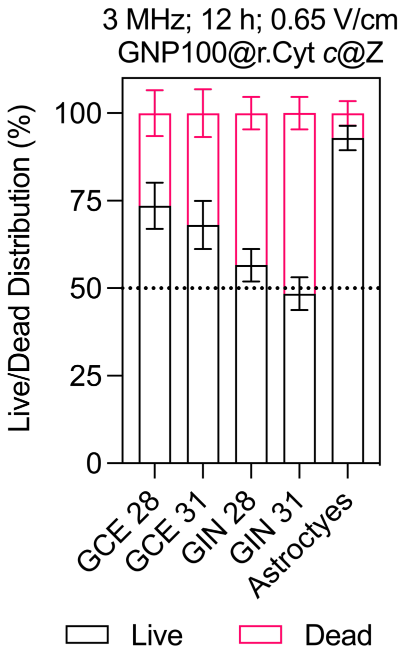


**Figure 13.** Quantification of live and dead cell population of images shown in supplementary fig. 12, calculated using ImageJ. Error bars represent the S.E.M. of mean obtained from the number of dead or live cells from 3 different images (1 image from each repeat) each containing 100 cells.

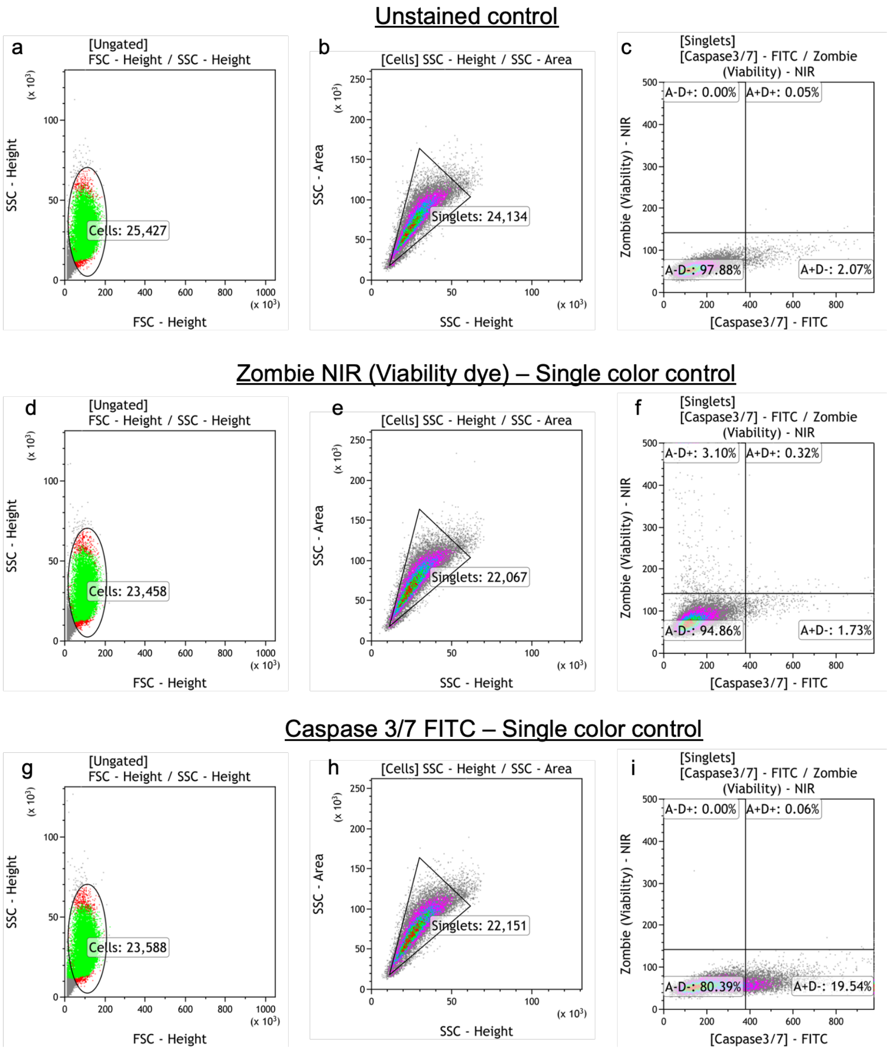


**Figure 14. Flow cytometry gating strategy.** The data from the flow cytometer was analysed using kaluza v2.1 software. **(a-c)** In the unstained control - the population was cells were chosen by plotting side scatter height (SSC) *vs* forward scatter height (FSC). To account for aggregated cell population, singlet population was obtained by plotting SSC area *vs* SSC height. The singlet cells were chosen and gated to cell population. This setting was applied to all other single (stained with either caspase FITC stain or Zombie NIR stain), double color controls (stained with both caspase FITC stain and Zombie NIR stain) and test samples. **(d-f)** The position of gates for Zombie (viability dye) NIR single color control were setup by moving vertical line across x-axis till the point it reaches the edge of cell population (excluding the overlapping events, in grey). These parameters were then updated across all the samples to ensure the identical position of the gates. **(g-i)** The position of gates for Caspase 3/7 FITC single color control were setup by moving horizontal line across y-axis till the point it reaches the edge of cell population (excluding the overlapping events, in grey). These parameters were then updated across all the samples to ensure the identical position of the gates.

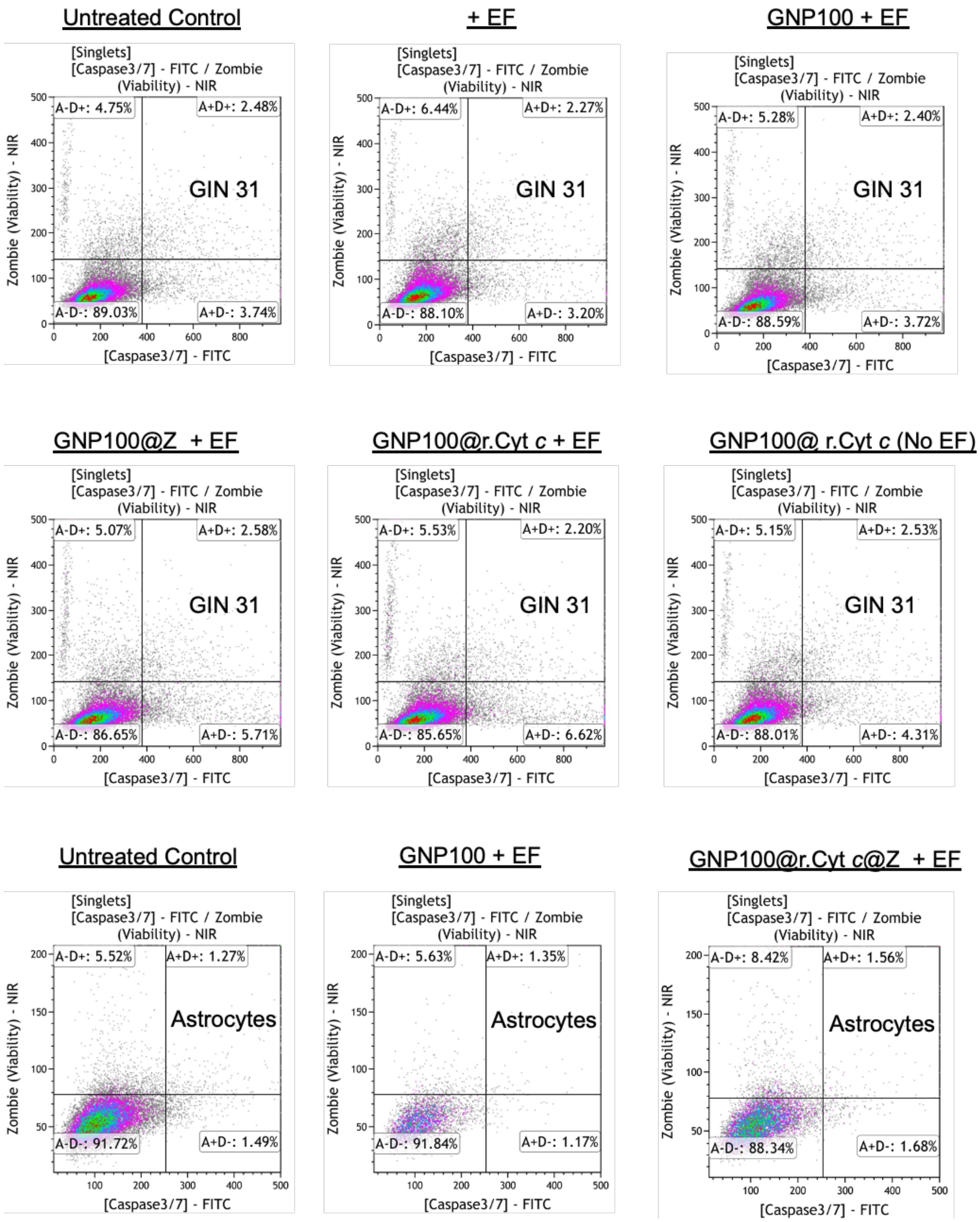


**Figure 15.** Representative flow cytometric analysis of GIN 31 cells (control samples) and cortical astrocytes (control and bio-nanoantennae) treated with CellEvent Caspase-3/7 Green to detect caspase activity and Zombie NIR dye to detect dead cell population after treatment with GNP100@r.Cyt *c*@Z for 8 h followed by AC-EFs stimulation (3MHz, 0.65V/cm) for 12 h.

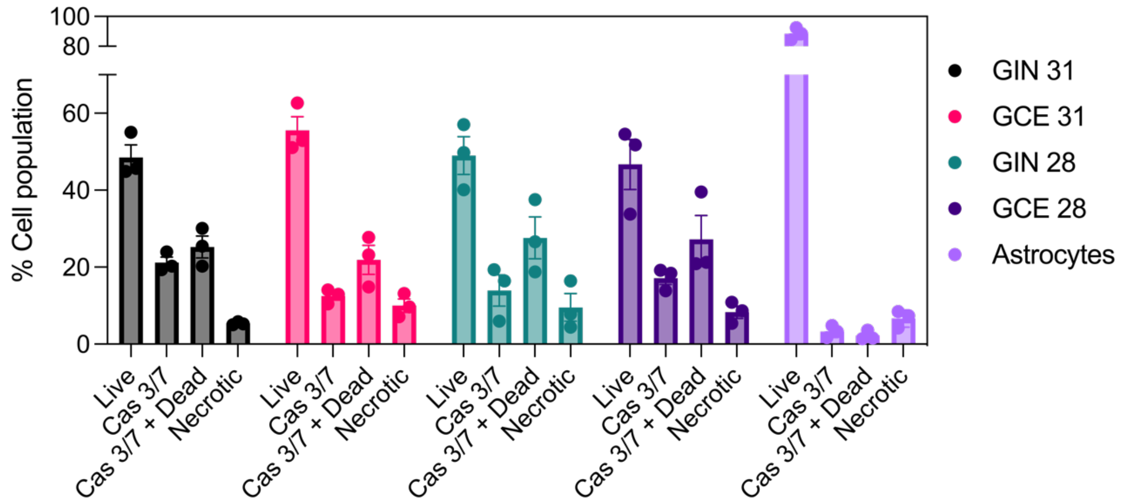


**Figure 16.** Quantification of flow cytometry data represented in Fig. 3d-g and supplementary Fig. 14. Error bars represent S.E.M. from triplicate experiments repeated thrice.

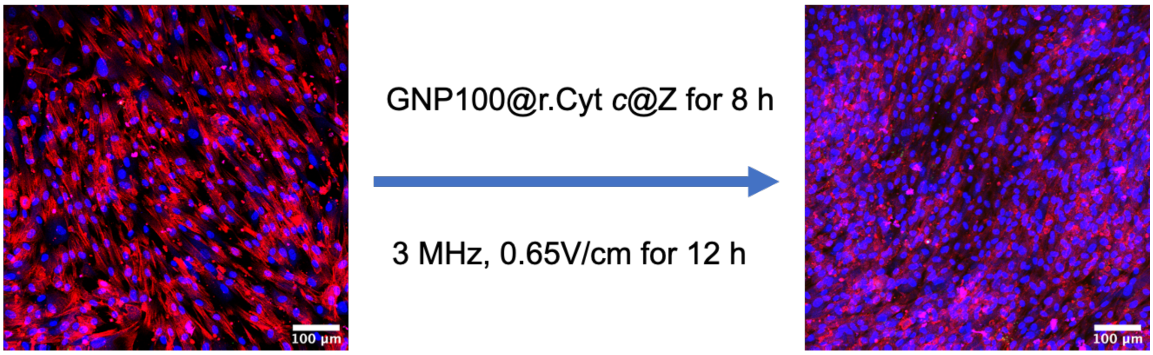


**Figure 17.** Confocal microscopy images to demonstrate caspase 3/7 activation in cortical astrocytes upon treatment bio-nanoantennae for 8 hours followed by stimulation with AC-EFs (3MHz, 0.65V/cm) for 12 h. Cells were fixed with paraformaldehyde followed by counterstaining with caspase 3/7 green detection kit, Cytopainter actin phalloidin (Texas Red 591 red), and Hoechst  nuclear stain (blue). Scale bar = 100 µm. To confirm caspase 3/7 activation, at least 3 confocal images (each with >100 astrocytes) from each repeated experiment were analysed.

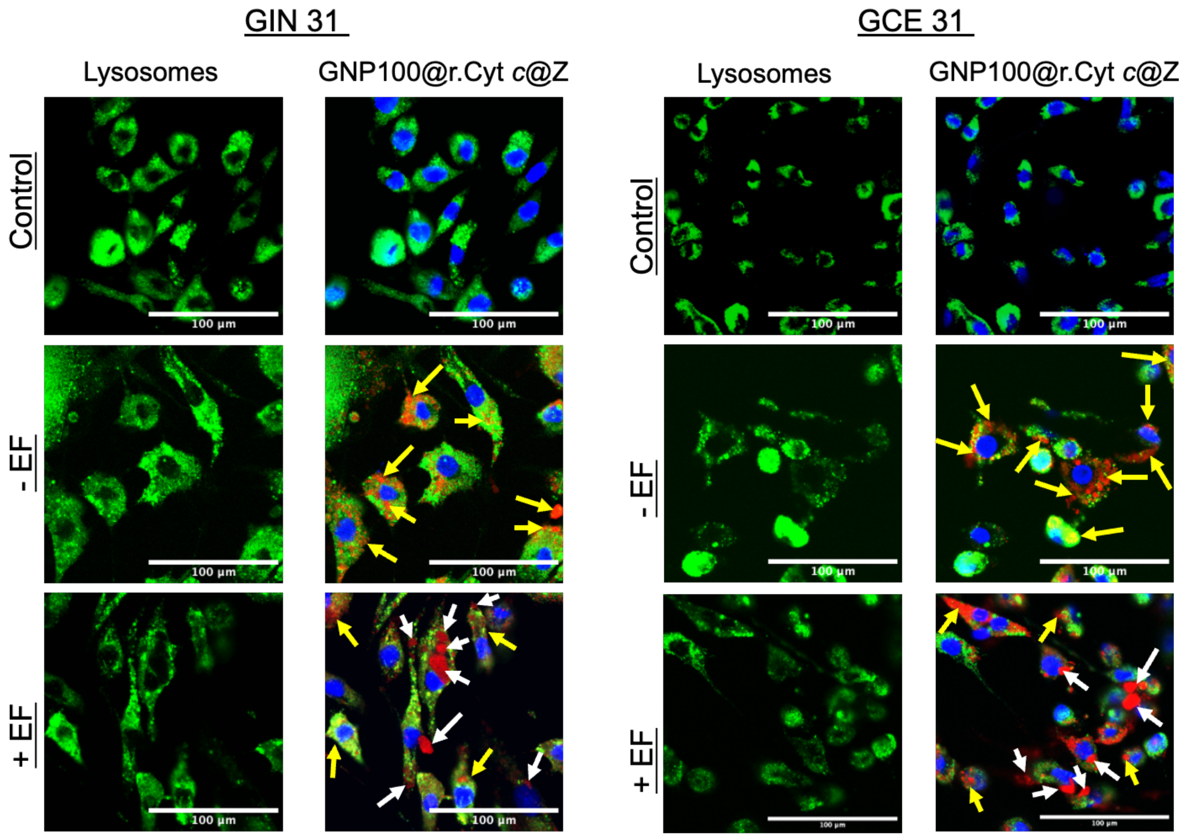


**Figure 18.** Confocal microscopy image to demonstrate cytoplasmic localisation of GNP100@r.Cyt *c*@Z immediately after the treatment with AC-EFs (3MHz, 0.65V/cm) for 12 h in presence of bio-nanoantennae. Cells were stained with lysotracker (green) and imaged using a Leica confocal microscope with DAPI (nuclei), GFP (endosomes), and Alexa 633 (GNP100@r.Cyt *c*@Z) filter settings. Scale bar = 100 µm. Yellow and white arrows indicate bio-nanoantennae are located within lysosomes and lysosomal escape, respectively. To confirm the localisation of bio-nanoantennae at least 60 cells were analysed.

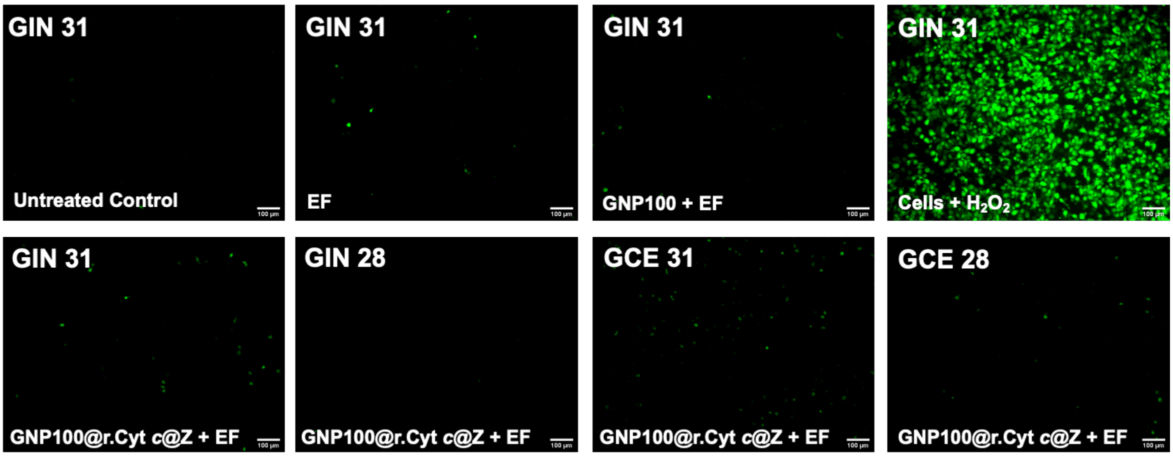


**Figure 19. Representative fluorescent microscopy images of oxidative damage caused by bifunctionalised AC-EFs responsive bio-nanoantennae in GBM cells.** GIN and GCE cells were treated with GNP100@r.Cyt *c*@Z for 8 h followed by AC-EFs stimulation (3MHz, 0.65V/cm) for 12 h. Finally, the reactive oxygen generation was analysed using DCFDA/$H_2$DCFDA - Cellular ROS Assay green detection Kit (ThermoFisher). Cells treated with 100 µM hydrogen peroxide for 30 minutes was used positive control. Scale bar = 100 µm. To evaluate oxidative damage by ROS, at least 3 fluorescence microscopy images (each with >100 cells) from each repeated experiment were analysed.

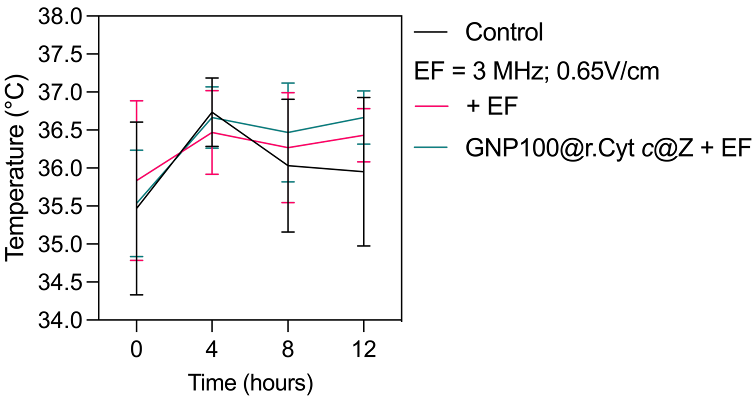


**Figure 20.** AC-EF (3MHz, 0.65V/cm) mediated change in solution temperature monitored over the course of experiment using NIR laser gun. Error bars represent mean ± S.D. obtained from triplicate experiments repeated thrice.

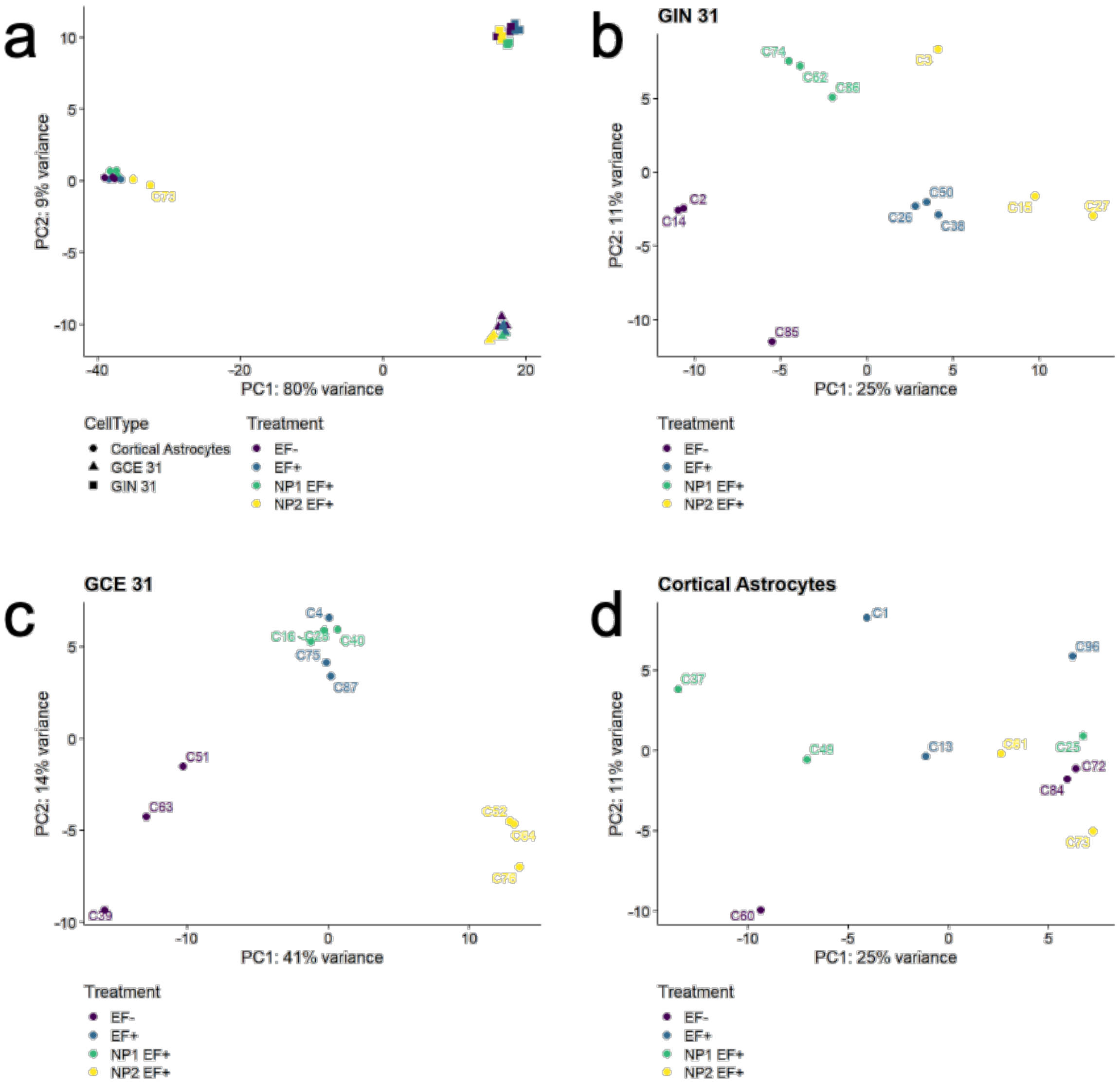


**Figure 21. Principal component analysis (PCA).** A variance stabilizing transformation was performed on the raw count matrix and 500 genes with the highest variance were used to plot the PCA. The variance was calculated agnostically to the pre-defined groups. PCA analysis **(a)** between different cell types and treatment groups, between different treatment within a cell type **(b)** GIN 31, **(c)** GCE 31, and **(d)** cortical astrocytes. In the figure treatment codes are as follow: - EF = Control (no treatment with either bio-nanoantennae or AC EFs); + EF = cells treated with AC EFs; NP1 EF+ = cell treated with GNP100@r.Cyt *c*, and NP2 EF+ = cells treated with GNP100@r.Cyt *c*@Z, for 8 h followed by 2-hour AC EFs.

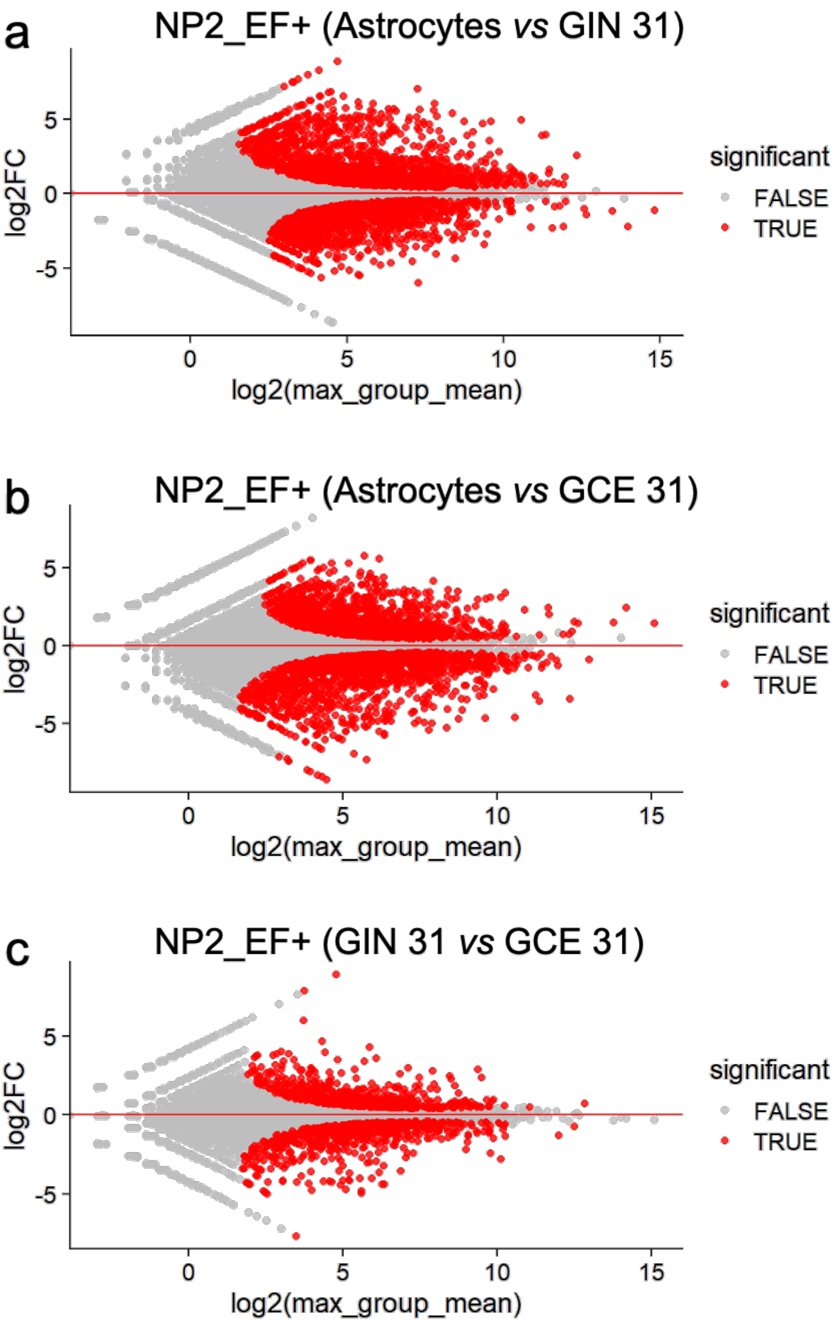


**Figure 22. MA plot representing statistical tests (log fold-change *vs* mean expression between two cell types when treated with NP2_EF+) of the differential gene expression analyses.** All significant differentially expressed genes are marked in red. (a) Cortical Astrocytes *vs* GIN 31, (b) Cortical Astrocytes *vs* GCE 31, and (c) GIN 31 *vs* GCE 31. Significant changes were defined as FDR<0.01. Note: NP2_EF+ = GNP100@r.Cyt *c*@Z followed by ES with AC EFs 3MHz, 0.65V/cm for 2 hours.

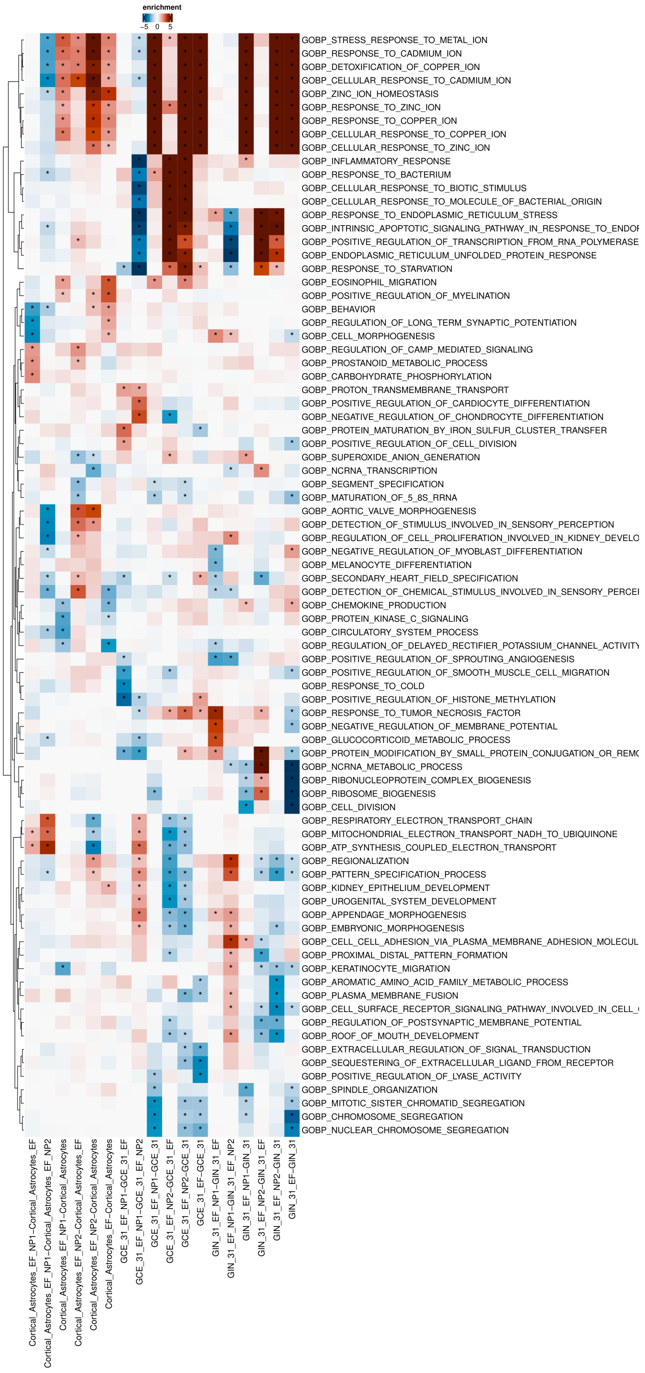


**Figure 23.** Gene-set Enrichment analysis (GSEA) of Gene Ontology Biological Processes (GOBP) is represented as a heatmap. The colour code is based on the normalized enrichment score (NES). The significantly enriched terms (adj p-value <0.05) are indicated by (*) symbol. For the Gene set enrichment analysis, we used one-tailed Fisher's exact test with 95% of confidence interval. The p-values were corrected for multiple comparisons with the Benjamini-Hochberg method.

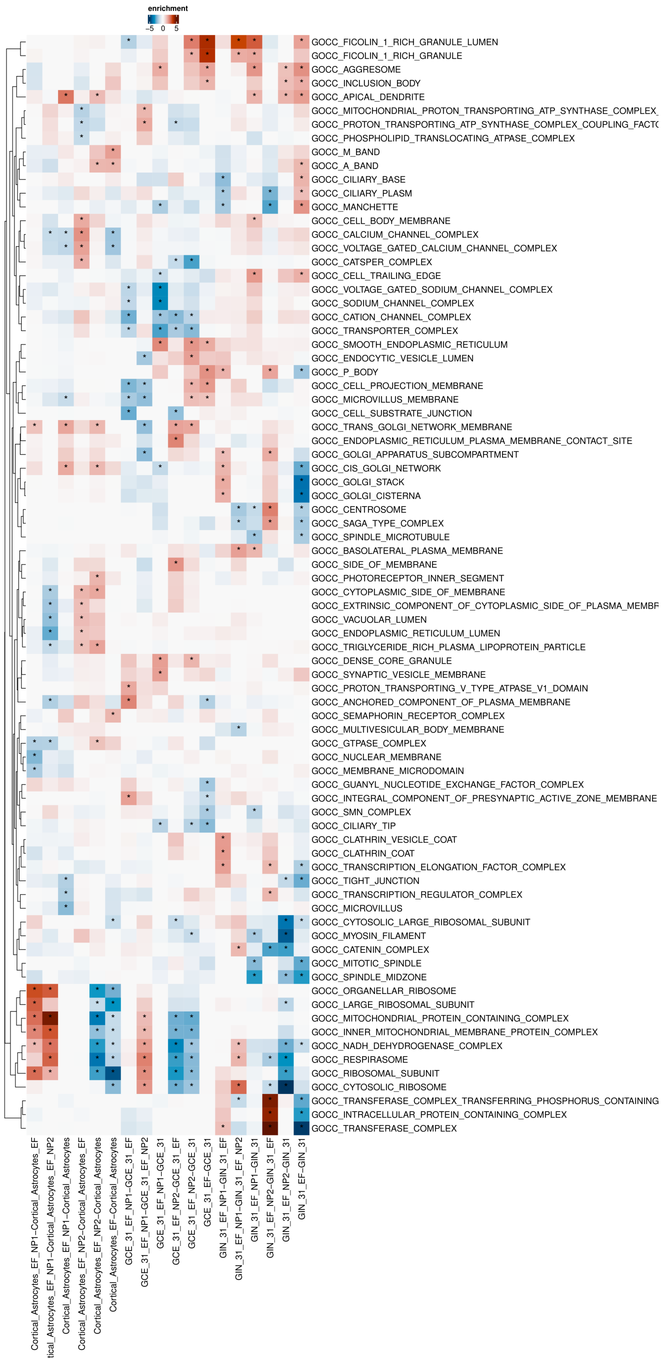

**Figure 24** Gene-set Enrichment analysis (GSEA) of Gene Ontology Cellular Components (GOCC) is represented as a heatmap. The colour code is based on the normalized enrichment score (NES). The significantly enriched terms (adj p-value <0.05) are indicated by (*) symbol. For the Gene set enrichment analysis, we used one-tailed Fisher's exact test with 95% of confidence interval. The p-values were corrected for multiple comparisons with the Benjamini-Hochberg method.

**Supplementary note 3:** Discussion of gene ontology and GSEA analysis of GOBP.

Gene ontology analysis revealed that most of the differentially upregulated genes such as *CXCL8* & *INHBA* in GCE 31, and *MT1G* & *HMOX1* in GIN/GCE 31 encodes proteins that are related with apoptosis, implying that apoptosis was upregulated after the treatment.[5-8] Furthermore, the upregulation of *HMOX1* (encodes a haem oxygenase) could be attributed to the presence of haem containing o.Cyt *c* and Z of bio-nanoantennae. On the other hand, most of downregulated genes such as *STC1* (GIN 31), *IGFBP5* (GIN/GCE 31), and *FBXO32* (GIN/GCE 31) are characteristic of angiogenesis in cancer proliferation, and tumour growth and metastasis, respectively.[9-11]

GBM cells derived from the tumour core and infiltrative margin, and cortical astrocytes, all exhibit significant upregulation of genes associated with cellular response to metal ion stimulus (Extended Data Figure **1a -** Cellular Response to Treatment). This is consistent with AC-EF responsive bio-nanoantennae specifically effecting metal ion response pathways, likely upstream of phenotypic changes induced by electrical-molecular signalling. However, to gain insight into differential sensitivities to AC-EF responsive bio-nanoantennae, we focused on cell-type specific pathways which are altered. Positive regulation of myelination and regulation of cyclic adenosine 3',5'-monophosphate (cAMP) mediated signalling pathways were the most significantly upregulated in astrocytes upon treatment (Extended Data Figure **1b** - Astrocyte Response to Treatment) based on normalized enrichment scores (adjusted p-value $<0.05$). Significantly upregulated genes within positive regulation of myelination include teneurin transmembrane protein (encoded by *Tenm4*), associated with myelin sheath formation,[12] and myelin regulator factor (encoded by *Myrf*), associated with central nervous system transcriptional activation of myelin production promoting genes.[13] It is unclear why regulation of myelination pathways is significantly upregulated in astrocytic monoculture (i.e., in the absence of co-cultured neurons) upon electrical stimulation; however, we cannot exclude non-

canonical, pleiotropic roles within this experimental context. Significant upregulation of genes within cAMP mediated signalling upon electrical stimulation may putatively be associated with β-adrenergic/cAMP-mediated morphological changes. Whilst β-adrenergic/cAMP-mediated is known to modify the geometry of extracellular space, affecting astrocyte function,[14] it is unclear how such modification may putatively be functionally associated with resistance to AC-EF responsive bio-nanoantennae.

For GBM cells derived from the tumour core (GCE31) and infiltrative margin (GIN31), inflammatory response, cellular response to biotic stimulus, response to endoplasmic reticulum stress, and intrinsic apoptotic signalling pathway, were amongst the most significantly upregulated pathways upon treatment (Extended Data Figure **1c** - GCE/GIN Response to Treatment) based on normalized enrichment scores (adjusted p-value <0.05). Collectively, this indicates an upstream acute stress response of GBM cells to an altered state or activity induced by electrical stimulation, with downstream triggering of apoptosis. That these biological processes are not significantly altered in cortical astrocytes warrants further interrogation experimentally, to determine why astrocytes are relatively resistant to electrical stimulation as a specific extracellular stress source.

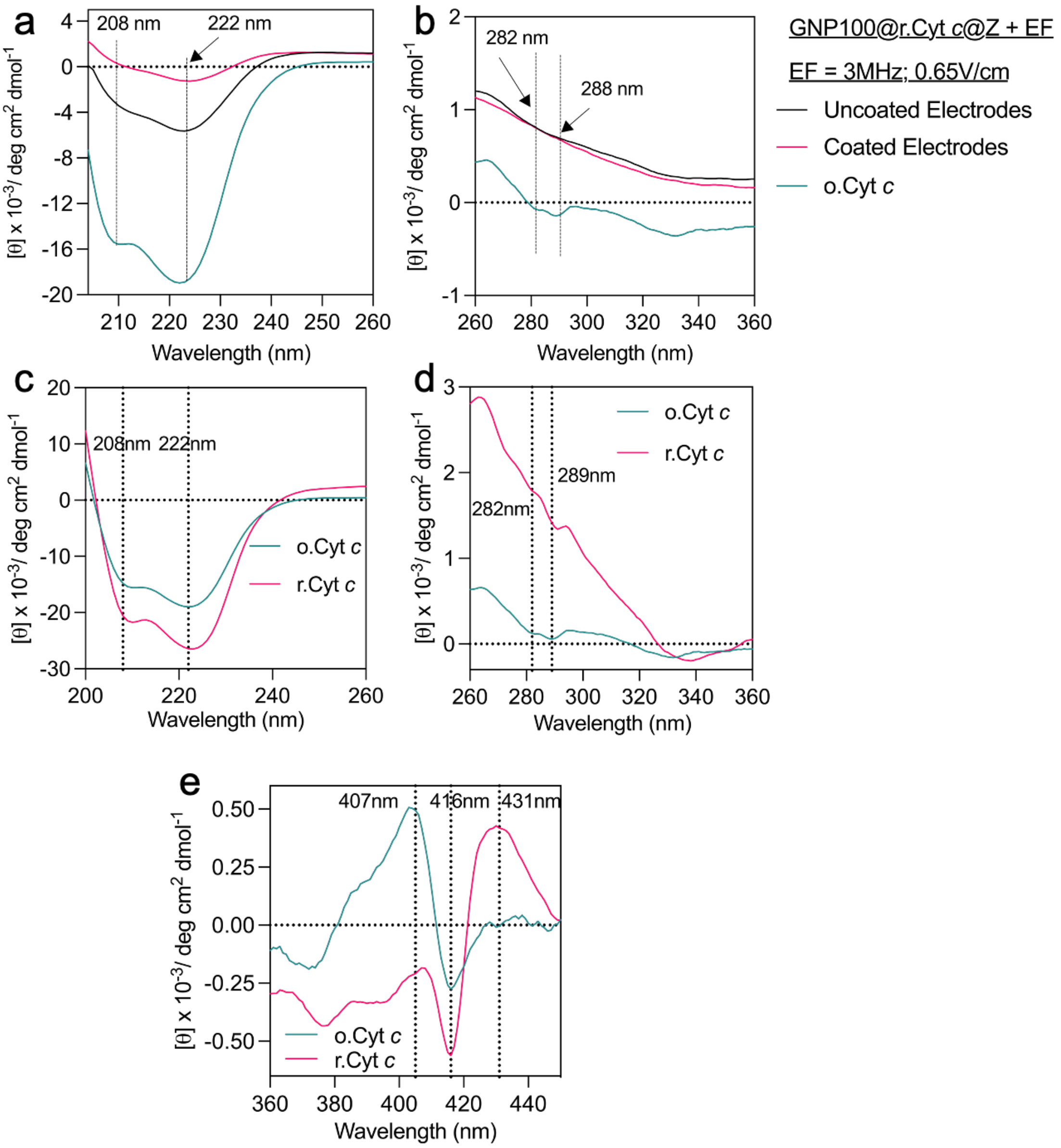


**Figure 25.** Circular Dichroism (CD) analysis. **(a-b)** Far-UV and near-UV CD of GNP100@r.Cyt *c*@Z (bio-nanoantennae) upon exposure with AC EFs of 3 MHz and 0.65 V/cm for 12 hours using coated and uncoated steel electrodes in PBS. The CD spectrum of bio-nanoantennae is compared with native oxidised cyt c (o.Cyt *c*). **(c-e)** CD spectrum of native r.Cyt *c* and o.Cyt *c* in PBS with no ES.

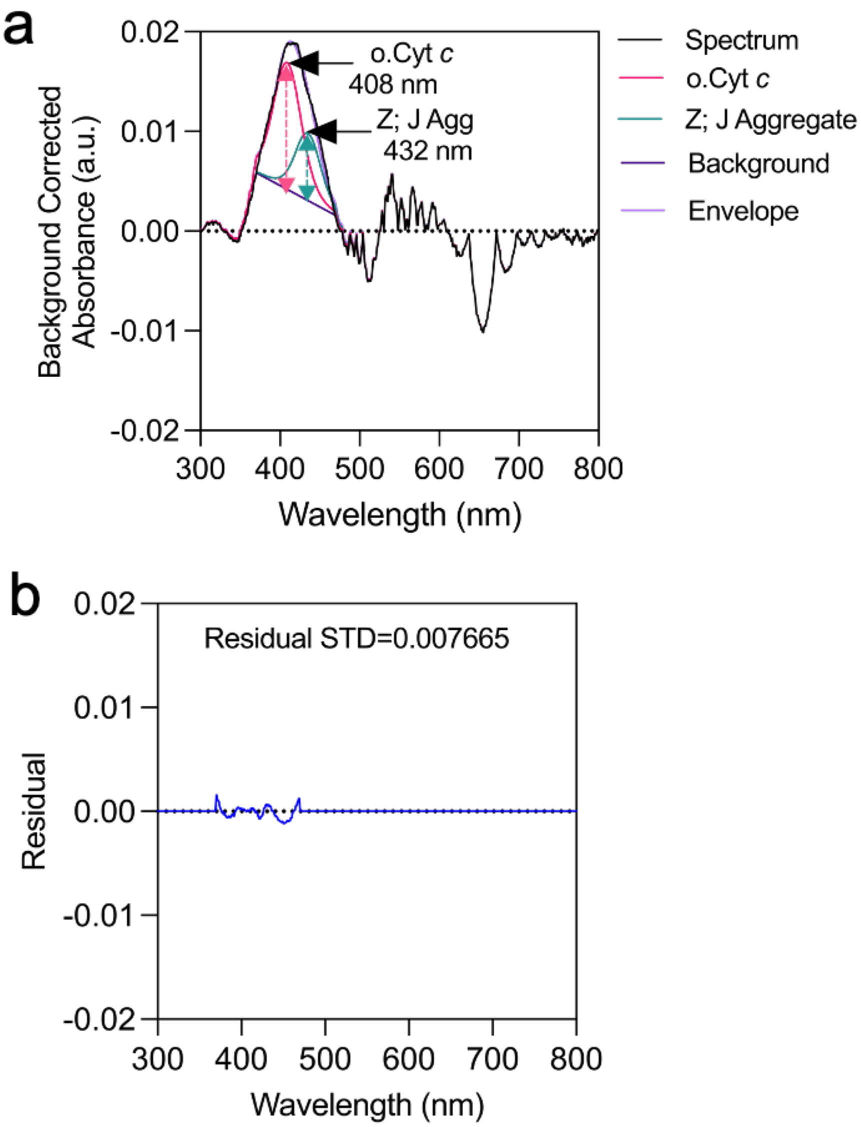


**Figure 26.** UV-Vis absorption spectrum of bio-nanoantennae after electrical stimulation with AC EFs (3 MHz, 0.65 V/cm). **(a)** Deconvolution of UV-Vis spectrum (shown in Fig. **5b & c** of main text) and curve fitting of GNP100@r.Cyt *c*@Z samples confirming that electrical stimulation induces change in the redox state of r.Cyt *c* to o.Cyt *c*, and Z. **(b)** Residual standard deviation obtained after curve fitting

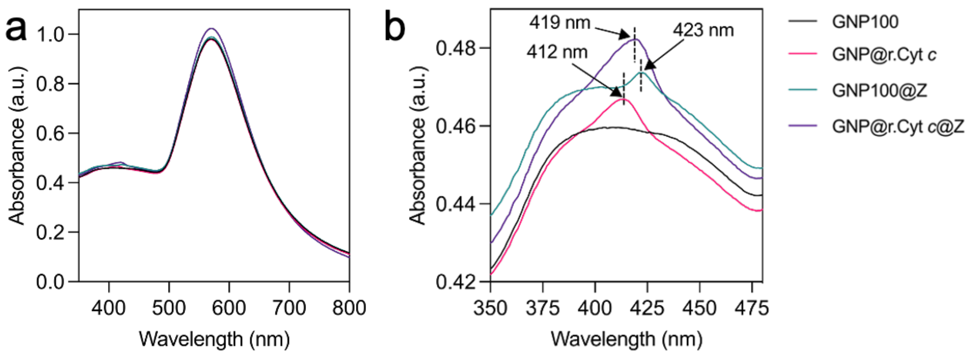


**Figure 27.** UV-Vis absorption spectrum of bio-nanoantennae without electrical stimulation after 12-hour incubation suggesting no change in redox state of r.Cyt *c* and Z.

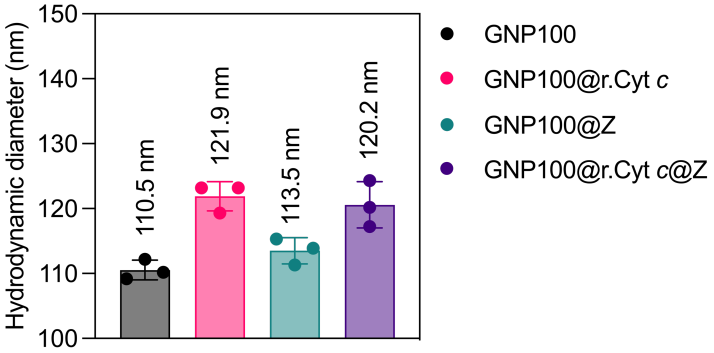


**Figure 28.** Hydrodynamic diameter of bio-nanoantennae (dispersed in ultra-pure water) after ES with AC-EFs (3 MHz, 0.65V). Error bars represent mean ± s.d. obtained from 3 individual experiments.

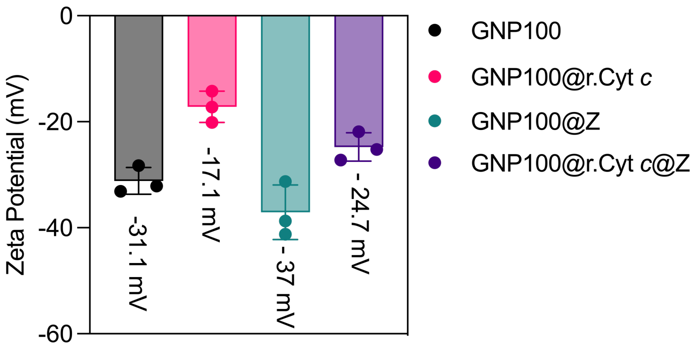


**Figure 29.** Zeta Potential of bio-nanoantennae (dispersed in ultra-pure water) after ES with AC-EFs (3 MHz, 0.65V). Error bars represent mean ± s.d. obtained from 3 individual experiments.

**Supplementary note 4:** Nanoscale wireless electrochemistry and *in vitro* QBET.

Discussion of the data obtained from CD, UV-Vis absorption spectroscopy, DLS, and Zeta potential measurements.

The soret CD band of GNP100@r.Cyt *c*@Z revealed two positive maxima (407 nm and 434 nm) suggesting the oxidation of the C haem centre after ES with AC EFs of 3 MHz at 0.65 V/cm. This effect was corroborated by comparison with CD of native o.Cyt *c*. Furthermore, one negative minimum (417 nm) was observed for native o.Cyt *c*, which was red shifted to 419 nm indicating slight perturbation around the haem moiety. On the other hand, when a control experiment was carried out by performing ES of GNP100@r.Cyt *c*@Z using insulated electrodes (electrodes were coated with an insulated polymer to stop electrochemical reaction), we did not observe any signals from r.Cyt *c* or o.Cyt *c*. To further ascertain nanoscale BPE, the UV-Vis spectrum of GNP100@r.Cyt *c*@Z was monitored after the ES (Fig. **5 b-c** and Supplementary Fig. **25 a-b**), which revealed an apparent blue shift in the absorbance peak of r.Cyt *c* from 412 nm to 408 nm and red shift in the absorbance peak of Z from 423 nm to 432 nm, which could be attributed to the oxidation of the haem moieties in Cyt *c* and a change in the local environment of Z leading to the formation of J aggregates,[15] respectively. We note that no shift of absorption peaks was observed without EF stimulation (Supplementary Fig. **26).** The obtained spectroscopic data confirms that we were successfully able to induce nanoscale wireless electrochemistry on the surface of GNP100@r.Cyt *c*@Z to modulate the redox state of Cyt *c* ($Fe^{2+}$ to $Fe^{3+}$) using remotely controlled AC EFs. In addition, there was no significant change in $h_d$ and $\zeta$ after electrical stimulation (ES) with AC electric fields (AC EF) of 3 MHz at 0.65V/cm suggesting the surface chemistry of the synthesised nanoantennae is stable (Supplementary Figs. **27-28**) even after ES. Thus, we demonstrate that the bio-nanoantennae act as bipolar electrodes and can modulate the redox state of the Cyt *c* under application of the EF. We envisage that these processes can be used to provide molecular communication with the cells to modulate their function.

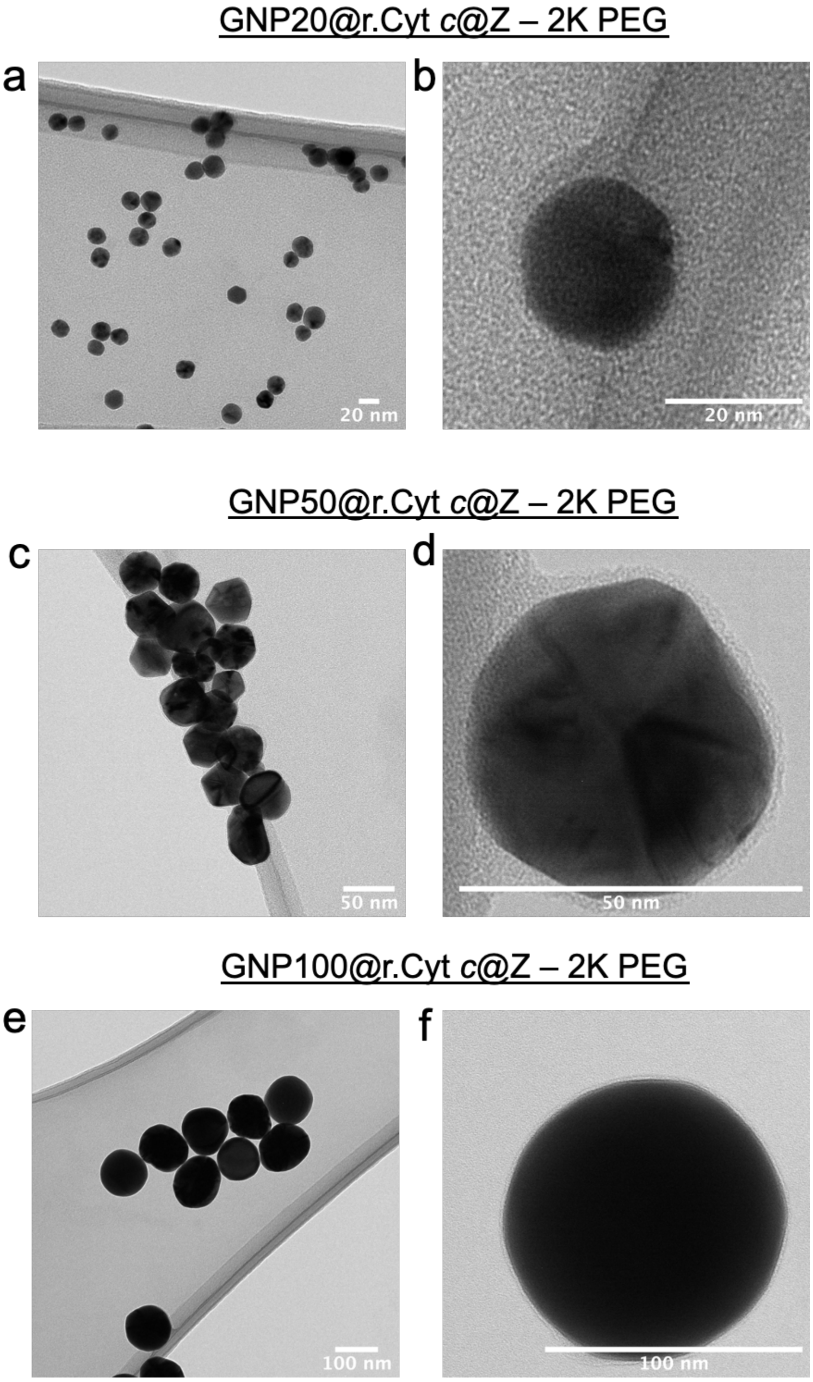


**Figure 30. Transmission electron microscope images of different size bio-nanoantennae functionalised using 2000 Da thiol-PEG-carboxylic linker. (a, c, and e)** TEM images of 20, 50, and 100 nm bifunctionalised bio-nanoantennae. **(b, d, and f)** High-resolution TEM images of 20, 50, and 100 nm bio-nanoantennae showing the presence of organic thiol-PEG linker. The TEM analysis was done on 3 different samples of bio-nanoantennae that were synthesised over the course of three individual experiments.

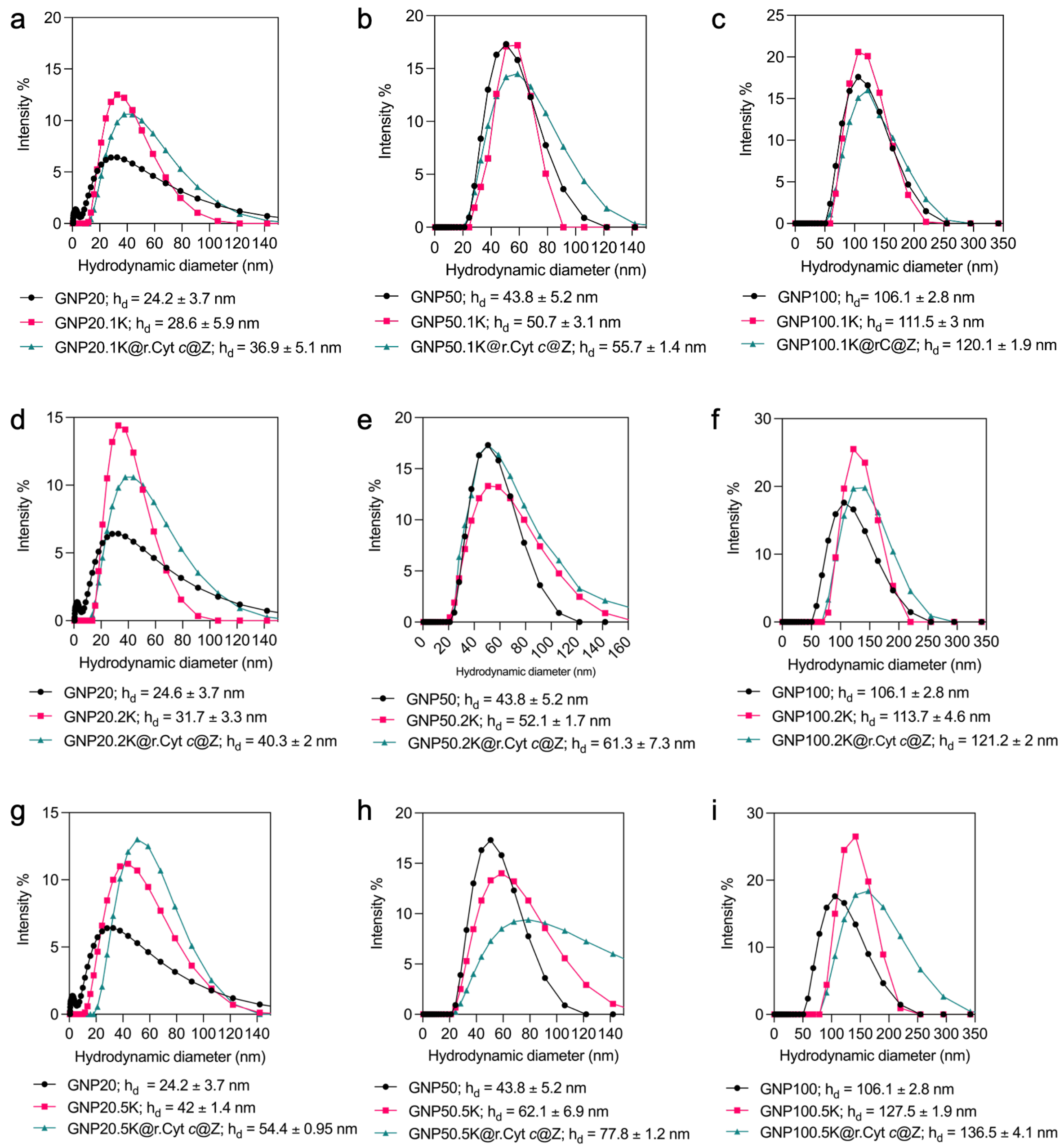


**Figure 31. Size distribution of different size bio-nanoantennae (GNP20@r.Cyt *c*@Z, GNP50@r.Cyt *c*@Z, and GNP100@r.Cyt *c*@Z) using linkers of various lengths analysed using dynamic light scattering (DLS). (a-c)** Hydrodynamic diameter ($h_d$) of bionanoatennae functionalised using 1000 Da (1K) **(d-f)** 2000 Da (2K) **(g-i)** Hydrodynamic diameter of 5000 Da (5K) thiol-PEG-carboxylic linker. Error bars represent mean ± standard error of mean (S.E.M.) obtained from triplicate experiments repeated thrice.

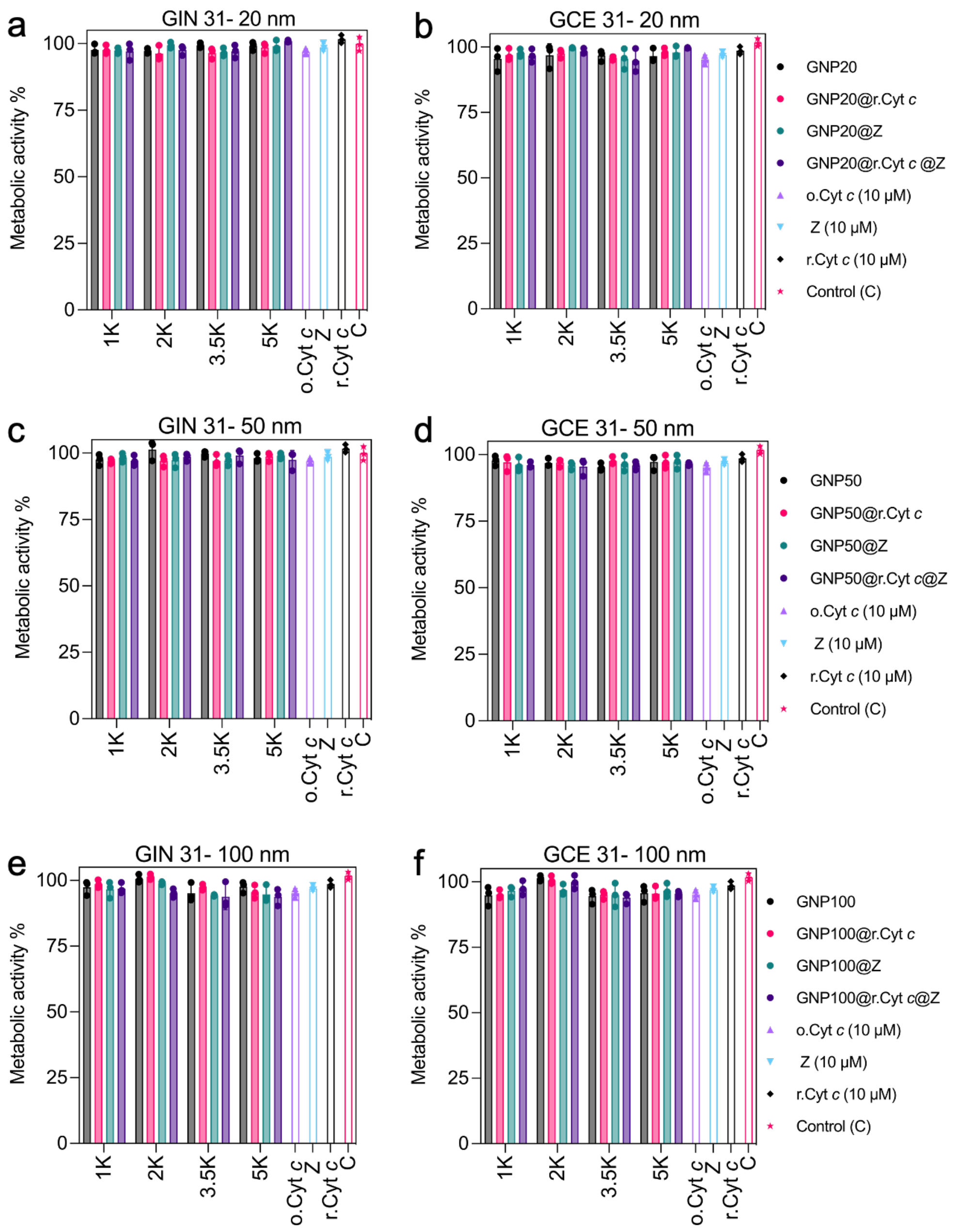


**Figure 32. *In vitro* toxicity of GNP20, GNP50, and GNP100 bio-nanoantennae functionalised using PEG linker of different lengths in the absence of electric fields. The cells were incubated with different sized nanocomposites at a concentration of 25 µg/mL and their toxicity were analyzed using PrestoBlue HS cell viability kit.** Metabolic activity of GIN 31 and GCE 31 upon treatment **(a-b)** 20 nm, **(c-d)** 50 nm, and **(e-f)** 100 nm with bio-nanoantennae functionalised using different thiol-PEG-carboxylic linker lengths. Error bars represent mean ± standard error of mean (S.E.M.) obtained from triplicate experiments repeated thrice. Statistical analysis was performed by applying 2-way ANOVA with a Tukey's post-test.

**Table 7.** *p* values obtained from statistical analysis of graph shown in Fig. 5d-f.

| **Bio-nanoantennae type** | **PEG linker length (in Da)** | ***p* value *vs* control (Treatment with GNP100@r.Cyt *c*@Z + EF 12 h)** |
|---|---|---|
| GNP20 | 1K | * = 0.0156 |
| | 2K | * = 0.021 |
| | 3.5K | ns |
| | 5K | ns |
| GNP50 | 1K | **** = <0.0001 |
| | 2K | ** = 0.0072 |
| | 3.5K | ns |
| | 5K | ns |
| GNP100 | 1K | **** = <0.0001 |
| | 2K | **** = <0.0001 |
| | 3.5K | *** = 0.00032 |
| | 5K | * = 0.0119 |

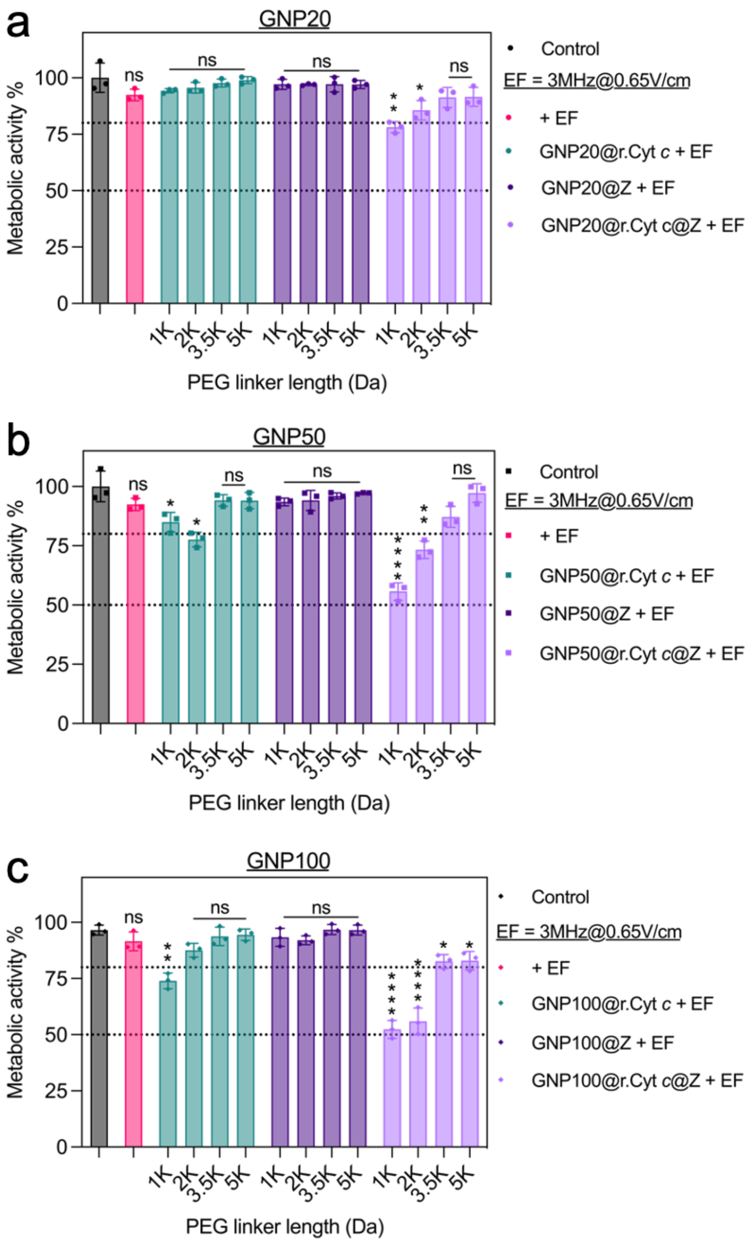


**Figure 33. Wireless electrical-molecular quantum signalling: *in vitro* electron tunnelling via bio-nanoantennae for inducing cell death in GBM cells.** Metabolic activity of GCE 31 cells as function of different sizebio-nanoantennae. **(a)** GNP20@r.Cyt *c*@Z, **(b)** GNP50@r.Cyt *c*@Z, and **(c)** GNP100@r.Cyt *c*@Z synthesised using various linker lengths (1K, 2K, 3.5K, and 5K Da). GIN 31 cells were treated with bifunctionalised bio-nanoantennae for 8 h followed by AC-EFs stimulation (3 MHz, 0.65V/cm) for 12 h. Error bars represent mean ± standard error of mean (S.E.M.) obtained from triplicate experiments repeated thrice. Statistical analysis was performed by applying 2-way ANOVA with a Tukey's post-test. The data was considered significant if *$p \leq 0.05$, **$p \leq 0.01$, ***$p \leq 0.001$, and ***$p \leq 0.0001$.

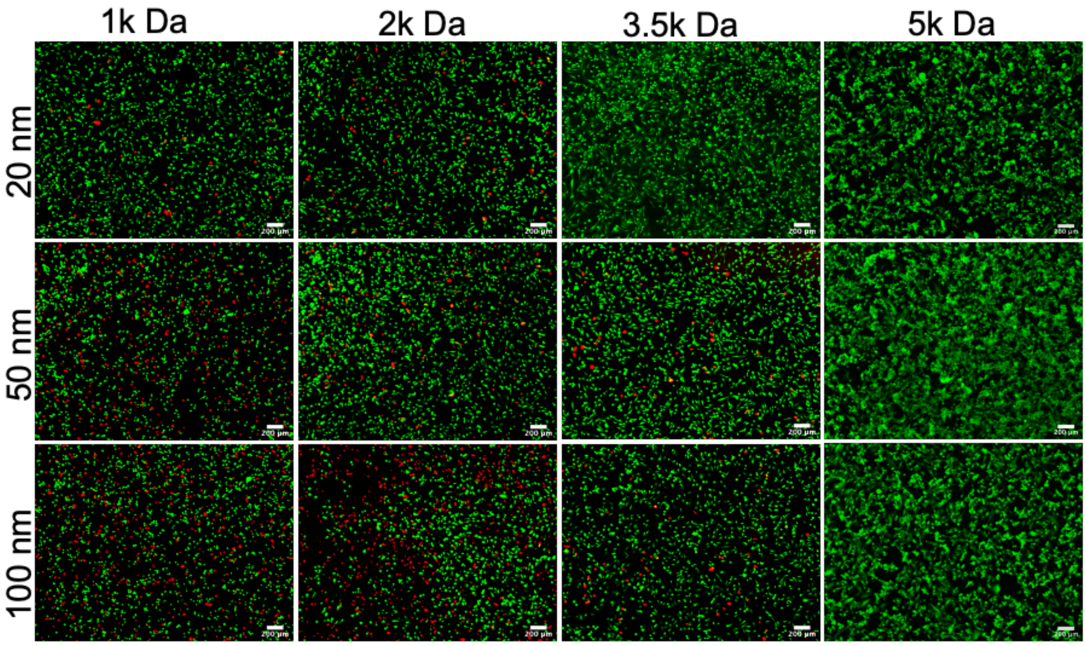


**Figure 34. Live/dead staining to demonstrate the effect of linker length and bio-nanoantennae size on GIN 31 killing effect.** GIN 31 cells were treated with bio-nanoantennae for 8 h followed by AC-EFs stimulation (3 MHz, 0.65V/cm) for 12 h. Post AC-EF treatment the cells were stained with calcein AM (green, live cells) and propidium iodide (red, dead cells). Live and dead cells were imaged using GFP and mTomato channel using a Nikon fluorescent microscope. Scale bar = 200 µm.

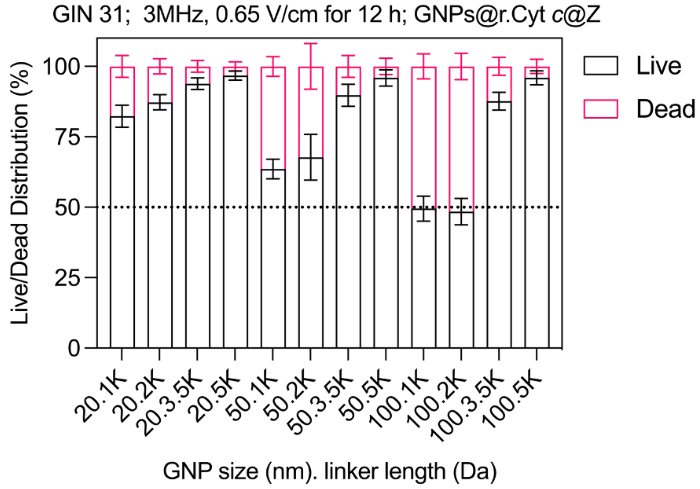


**Figure 35.** Quantification of live and dead cell population (shown in supplementary figure 33) to demonstrate the effect of linker length and bio-nanoantennae size on GIN 31 killing effect, calculated using ImageJ. Error bars represent mean ± S.E.M. obtained from the number of dead or live cells from 3 different images (1 image from each repeat) each containing 100 cells.

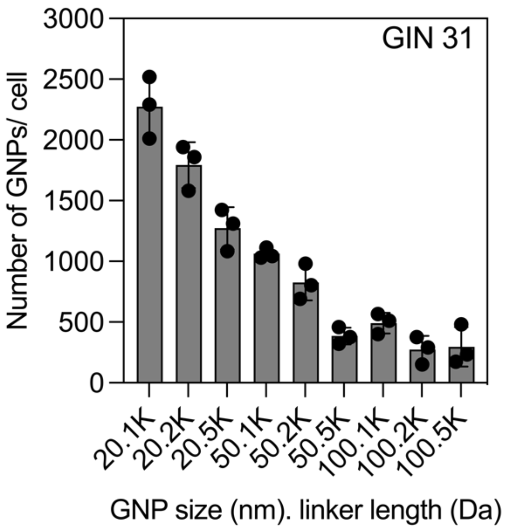


**Figure 36.** Number of bio-nanoantennae (different core and linker size) per GIN 31 cell calculated using ICP-MS to elucidate their cellular association. Error bars represent mean ± standard error of mean (S.E.M.) obtained from triplicate experiments repeated twice.

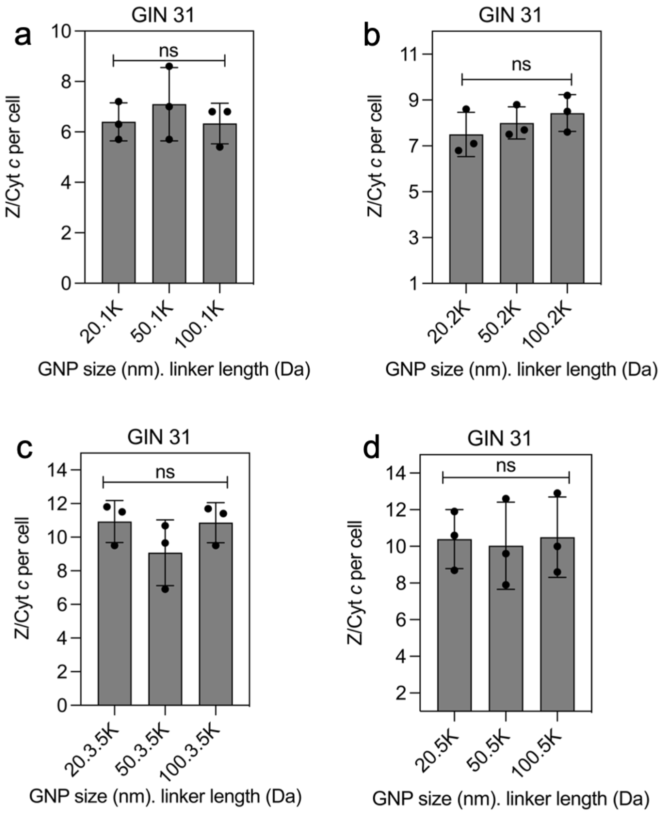


**Figure 37** (**a-d**) Ratio of zinc porphyrin (Z) to Cyt *c* per GIN 31 cells determined from number of bio-nanoantennae per GIN 31 cells calculated using ICP-MS analysis. Error bars represent mean ± standard error of mean (S.E.M.) obtained from triplicate experiments repeated twice. Statistical analysis was performed by applying 1-way ANOVA.

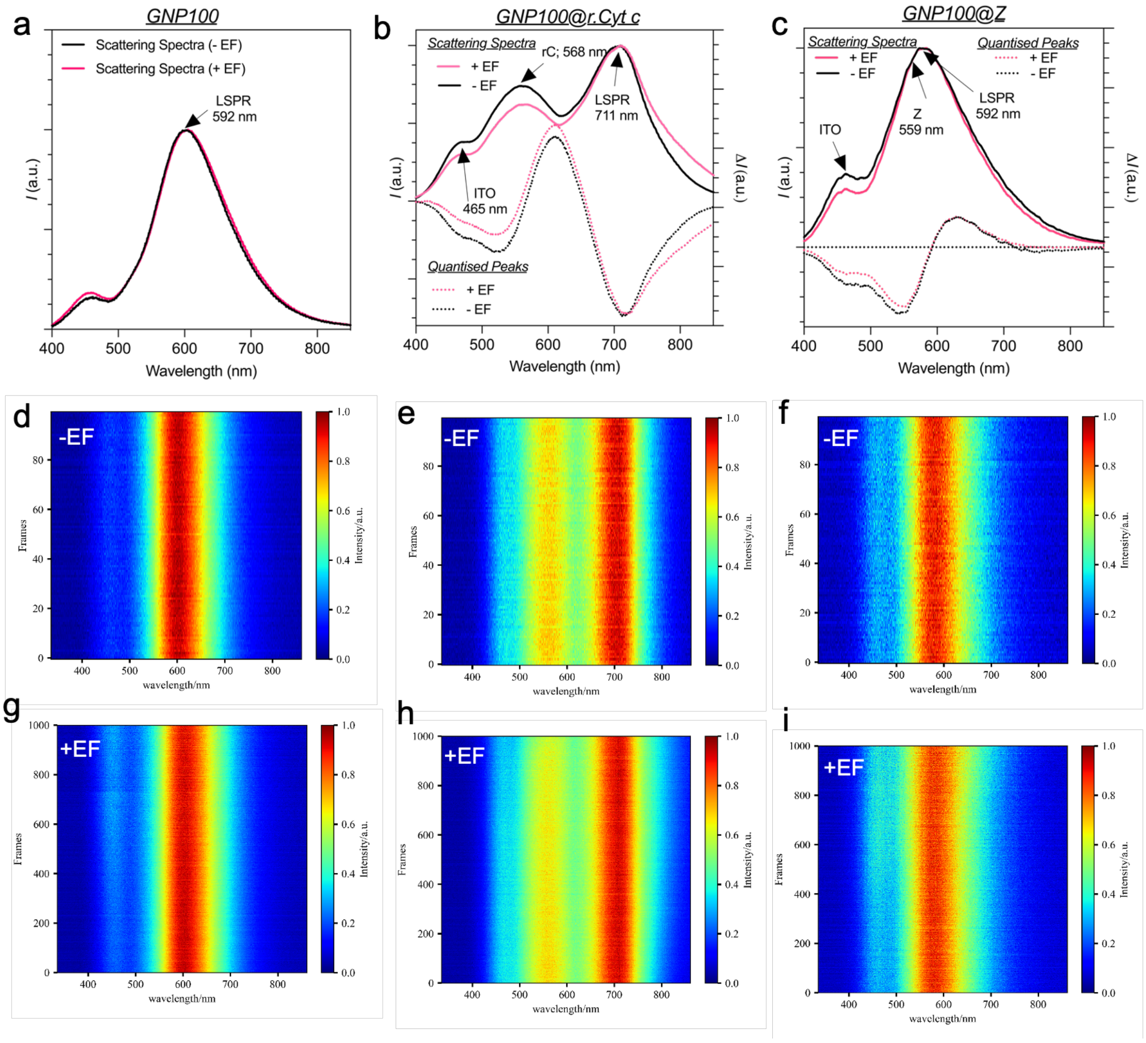


**Figure 38. PRS scattering spectra and quantised peaks to demonstrated QBET**. **(a-c)** Scattering spectra of GNP100 and corresponding 2D heat maps representing PRERT. **(d-f)** Scattering spectra and spectra difference for QBET obtained for GNP100@r.Cyt *c* and **(g-i)** GNP100@Z. The quantised peaks were obtained from the difference of scattering spectra between the samples functionalised with r.Cyt *c*, and Z using 2000 Da linker and GNP 100 nm. Solid curves are captured scattering spectra (linked to left axis), and dashed curves are quantised peaks i.e., the corresponding spectra difference (linked to right axis).

**Supplementary note 5:** Discussion on the role of that linker length, applied frequency and voltage for QBET mediated electrical-molecular communication and inducing cancer cell apoptosis.

The role of linker length can be explained from the findings shown in **fig.** 5 d-f and supplementary **figs.** 32-34. This data indicates that bio-nanoantennae synthesised using 1KDa and 2KDa linkers with an average length of 2.1 nm and 3.5 nm, respectively, showed the highest reduction in metabolic activity/viability of GBM cells, while bio-nanoantennae synthesised using 3.5 kDa and 5kDa with an average length of 6.7 nm and 8.7 nm, respectively did not show any significant change. This result suggests that the quantum tunnelling mediated effect can only be usefully operated up to approximately 3.5 nm. This is associated with the energy potential barrier associated with the ligand. The probability of an electron traversing the linker length becomes infinitely small as distance between electron donor and acceptor is increased, thus explaining negligible response to bio-nanoantennae with larger linker lengths. Furthermore, resonant frequency is important as this matches the unique energetic barrier required to cause evanescence of the electron coupled with voltage resulting in tunnelling.[16] This effect is also consistent with literature based on theoretical modelling, suggesting that alternating current frequency only above >MHz crosses the cell membrane.[17] The applied potential was critical in inducing redox reaction on nanoantennae surfaces in overcoming the potential barrier. In this work, an overpotential 7.5 times higher than the formal electrode potential (E°) of bio-nanoantennae was shown to induce redox reactions which results from their polarisation under EF, thus causing a voltage gradient along them with a maximum potential difference at their poles. This effect is consistent with the observation that membrane inserted CNT porins can allow on demand electron transfer to reduce gold salts at the cell membrane using direct current (DC). We observed gold (Au) reduction at ≥2V, which was twice that of the standard potential of Au (0.9V).[18] This further explains the obtained data (supplementary **fig. 8**) where cell killing due to the bipolar effect was only observed at high frequency (1MHz-3MHz) and potential higher than E°. Furthermore, from the therapy perspective, it is known that the redox properties of Cyt *c* are influenced by the reducing environment of the cytosol,[19] which results in its inactivation and eventually inhibition of Cyt *c* mediated apoptosis.[20] Therefore, it is envisaged that this approach of remote-control activation and endo/lysosomal escape of Cyt *c* on bio-nanoantennae would provide enhanced therapeutics at targeted site compared to previously reported studies.[21-23]

## 2. References


1 Potts, J., Jain, A., Amabilino, D. B., Rawson, F. & Pérez-García, L. Molecular Surface Quantification of Multi-Functionalized Gold Nanoparticles Using UV-Vis Spectroscopy Deconvolution. (2022).

2 Chen, X., Ferrigno, R., Yang, J. & Whitesides, G. M. Redox properties of cytochrome c adsorbed on self-assembled monolayers: a probe for protein conformation and orientation. *Langmuir* **18**, 7009-7015 (2002).

3 Neumann-Spallart, M. & Kalyanasundaram, K. On the one and two-electron oxidations of water-soluble zinc porphyrins in aqueous media. *Zeitschrift für Naturforschung B* **36**, 596-600 (1981).

4 Lavagnini, I., Antiochia, R. & Magno, F. An extended method for the practical evaluation of the standard rate constant from cyclic voltammetric data. *Electroanalysis: An International Journal Devoted to Fundamental and Practical Aspects of Electroanalysis* **16**, 505-506 (2004).

5 Brat, D. J., Bellail, A. C. & Van Meir, E. G. The role of interleukin-8 and its receptors in gliomagenesis and tumoral angiogenesis. *Neuro-oncology* **7**, 122-133 (2005).

6 Kore, R. A. *et al.* Hypoxia-derived exosomes induce putative altered pathways in biosynthesis and ion regulatory channels in glioblastoma cells. *Biochemistry and biophysics reports* **14**, 104-113 (2018).

7 Wang, Y. *et al.* MT1G serves as a tumor suppressor in hepatocellular carcinoma by interacting with p53. *Oncogenesis* **8**, 1-11 (2019).

8 Luu Hoang, K. N., Anstee, J. E. & Arnold, J. N. The diverse roles of heme oxygenase-1 in tumor progression. *Frontiers in immunology* **12**, 658315 (2021).

9 Xiong, Y. & Wang, Q. STC1 regulates glioblastoma migration and invasion via the TGF-β/SMAD4 signaling pathway. *Molecular medicine reports* **20**, 3055-3064 (2019).

10 Dong, C., Zhang, J., Fang, S. & Liu, F. IGFBP5 increases cell invasion and inhibits cell proliferation by EMT and Akt signaling pathway in Glioblastoma multiforme cells. *Cell division* **15**, 1-9 (2020).

11 Sahu, S. K. *et al.* FBXO32 promotes microenvironment underlying epithelial-mesenchymal transition via CtBP1 during tumour metastasis and brain development. *Nature communications* **8**, 1-18 (2017).

12 Hor, H. *et al.* Missense mutations in TENM4, a regulator of axon guidance and central myelination, cause essential tremor. *Human molecular genetics* **24**, 5677-5686 (2015).

13 Bujalka, H. *et al.* MYRF is a membrane-associated transcription factor that autoproteolytically cleaves to directly activate myelin genes. *PLoS biology* **11**, e1001625 (2013).

14 Vardjan, N., Kreft, M. & Zorec, R. Dynamics of β-adrenergic/cAMP signaling and morphological changes in cultured astrocytes. *Glia* **62**, 566-579 (2014).

15 Occhiuto, I. G. *et al.* Controlling J-Aggregates formation and chirality induction through demetallation of a zinc (II) water soluble porphyrin. *International Journal of Molecular Sciences* **21**, 4001 (2020).

16 Henkel, C. *et al.* Resonant tunneling induced enhancement of electron field emission by ultra-thin coatings. *Scientific Reports* **9**, 6840 (2019).

17 Taghian, T., Narmoneva, D. & Kogan, A. Modulation of cell function by electric field: a high-resolution analysis. *Journal of the Royal Society Interface* **12**, 20150153 (2015).

18 Hicks, J. M. *et al.* Electric Field Induced Biomimetic Transmembrane Electron Transport Using Carbon Nanotube Porins. *Small* **17**, 2102517 (2021).

19 Ripple, M. O., Abajian, M. & Springett, R. Cytochrome c is rapidly reduced in the cytosol after mitochondrial outer membrane permeabilization. *Apoptosis* **15**, 563-573 (2010).

20 Vaughn, A. E. & Deshmukh, M. Glucose metabolism inhibits apoptosis in neurons and cancer cells by redox inactivation of cytochrome c. *Nature cell biology* **10**, 1477-1483 (2008).

21 Zhang, B. *et al.* Cytochrome c end-capped mesoporous silica nanoparticles as redox-responsive drug delivery vehicles for liver tumor-targeted triplex therapy in vitro and in vivo. *Journal of Controlled Release* **192**, 192-201 (2014).

22 Kim, S. K., Foote, M. B. & Huang, L. The targeted intracellular delivery of cytochrome C protein to tumors using lipid-apolipoprotein nanoparticles. *Biomaterials* **33**, 3959-3966 (2012).

23 Guo, C. *et al.* Gold nanoparticle-guarded large-pore mesoporous silica nanocomposites for delivery and controlled release of cytochrome c. *Journal of colloid and interface science* **589**, 34-44 (2021).
