# Supplementary material for: Wireless electrical–molecular quantum signalling for cancer cell apoptosis
Source: Nat Nanotechnol. 2023 Sep 14;19(1):106–14. doi: 10.1038/s41565-023-01496-y (PMC10796273; doi:10.1038/s41565-023-01496-y)
Supplement: Supplementary file 2 — Reporting Summary [file 41565_2023_1496_MOESM2_ESM.pdf]

nature portfolio

Corresponding author(s): Frankie James Rawson NNANO-23030589A

Last updated by author(s): May 13, 2023

Reporting Summary

Nature Portfolio wishes to improve the reproducibility of the work that we publish. This form provides structure for consistency and transparency in reporting. For further information on Nature Portfolio policies, see our Editorial Policies and the Editorial Policy Checklist.

Statistics

For all statistical analyses, confirm that the following items are present in the figure legend, table legend, main text, or Methods section.

n/a	Confirmed	
☐	☒	The exact sample size (*n*) for each experimental group/condition, given as a discrete number and unit of measurement
☐	☒	A statement on whether measurements were taken from distinct samples or whether the same sample was measured repeatedly
☐	☒	The statistical test(s) used AND whether they are one- or two-sided<br>*Only common tests should be described solely by name; describe more complex techniques in the Methods section.*
☐	☒	A description of all covariates tested
☐	☒	A description of any assumptions or corrections, such as tests of normality and adjustment for multiple comparisons
☐	☒	A full description of the statistical parameters including central tendency (e.g. means) or other basic estimates (e.g. regression coefficient) AND variation (e.g. standard deviation) or associated estimates of uncertainty (e.g. confidence intervals)
☐	☒	For null hypothesis testing, the test statistic (e.g. *F*, *t*, *r*) with confidence intervals, effect sizes, degrees of freedom and *P* value noted<br>*Give P values as exact values whenever suitable.*
☒	☐	For Bayesian analysis, information on the choice of priors and Markov chain Monte Carlo settings
☒	☐	For hierarchical and complex designs, identification of the appropriate level for tests and full reporting of outcomes
☒	☐	Estimates of effect sizes (e.g. Cohen's *d*, Pearson's *r*), indicating how they were calculated

Our web collection on statistics for biologists contains articles on many of the points above.

Software and code

Policy information about availability of computer code

Data collection	Device: infinite 200Pro-Tecan i-control , 2.0.10.0<br>Flow cytometry- ID7000 Spectral Flow Cytometer using Kaluza software (v2.1)<br>electrochemistry-Nova<br>Image J (Fiji) for fluorescence imaging and TEM<br>Fluorescent microscopy Leica Application Suite X (LAS X)
Data analysis	Stats and other graphs - GraphPad Prism v9.4.1 software (GraphPad Software, Inc)<br>Image J (Fiji) for fluorescence imaging<br>Microsoft excel for calculation and other analysis<br>Casa XPS 2.3.17 for deconvolution<br>R software - version 4.2.1: All the analyses were performed by using R programing language<br>Limma (Linear Models for Microarray Data) package – version 3.54.2: Used for differential gene expression<br>clusterProfiler package – version 4.6.2: For enrichment of pathways<br>complexheatmap package – version 2.12.0: For creating heatmaps<br>ggplot2 – version 3.4.2: Used for volcano and other plots

For manuscripts utilizing custom algorithms or software that are central to the research but not yet described in published literature, software must be made available to editors and reviewers. We strongly encourage code deposition in a community repository (e.g. GitHub). See the Nature Portfolio guidelines for submitting code & software for further information.

Data

Policy information about availability of data

All manuscripts must include a data availability statement. This statement should provide the following information, where applicable:

- Accession codes, unique identifiers, or web links for publicly available datasets
- A description of any restrictions on data availability
- For clinical datasets or third party data, please ensure that the statement adheres to our policy

Source data (main text figures 2-6 and extended data figure 1 is provided with this paper). All other data associated with this manuscript (including the supplementary information) can be found at http://doi.org/10.17639/nott.7303. Complete data set of transcriptomics analysis can be found at Gene Expression Omnibus (GEO) accession number - GSE233380.

Research involving human participants, their data, or biological material

Policy information about studies with human participants or human data. See also policy information about sex, gender (identity/presentation), and sexual orientation and race, ethnicity and racism.

Reporting on sex and gender	N/A
Reporting on race, ethnicity, or other socially relevant groupings	N/A
Population characteristics	N/A
Recruitment	N/A
Ethics oversight	N/A

Note that full information on the approval of the study protocol must also be provided in the manuscript.

Field-specific reporting

Please select the one below that is the best fit for your research. If you are not sure, read the appropriate sections before making your selection.

☒ Life sciences ☐ Behavioural & social sciences ☐ Ecological, evolutionary & environmental sciences

For a reference copy of the document with all sections, see nature.com/documents/nr-reporting-summary-flat.pdf

Life sciences study design

All studies must disclose on these points even when the disclosure is negative.

Sample size	no sample size was determined through calculation but base on the following published papers https://www.nature.com/articles/s41565-020-00812-0, https://www.nature.com/articles/s41467-019-09226-6 https://www.nature.com/articles/s41565-023-01378-3, https://www.nature.com/articles/s41467-021-26694-x
Data exclusions	No data was excluded form the analysis
Replication	We have repeated the experiment n=3 N=3 all experimental attempts were successful
Randomization	All measurements were random
Blinding	For PRET measurements samples were sent number coded with number object identifier. This was to protect IP and to enhance the data integrity. No other blinding was performed as the was no allocation of subgroups.

Reporting for specific materials, systems and methods

We require information from authors about some types of materials, experimental systems and methods used in many studies. Here, indicate whether each material, system or method listed is relevant to your study. If you are not sure if a list item applies to your research, read the appropriate section before selecting a response.

Materials & experimental systems

n/a	Involved in the study
☒	☐ Antibodies
☐	☒ Eukaryotic cell lines
☒	☐ Palaeontology and archaeology
☒	☐ Animals and other organisms
☒	☐ Clinical data
☒	☐ Dual use research of concern
☒	☐ Plants

Methods

n/a	Involved in the study
☒	☐ ChIP-seq
☐	☒ Flow cytometry
☒	☐ MRI-based neuroimaging

Eukaryotic cell lines

Policy information about cell lines and Sex and Gender in Research

Cell line source(s)	Glioma INvasive Marginal (GIN) cells were isolated from the 5-aminolevulinic acid (5-ALA) fluorescing infiltrative tumour margin and Glioma Core Enhanced (GCE) cells were isolated from the core central region of the tumor from the glioblastoma (GBM) patients, who underwent surgery at the Queen's Medical Centre, University of Nottingham (Nottingham, UK) using previously described method.3 Low-passage U251 cell lines (purchased from ATCC, USA) and patient-derived. Human derived cortical astrocytes (HCOA. Cat. No. 1800, Batch No. 24490, ScienCell) and cerebellar astrocytes (HCEA. Cat. No. 1810, ScienCell) . The Human Intrahepatic Biliary Epithelial Cells (HIBEpiC) isolated from human healthy liver tissue were acquired from Innoprot (Derio, Spain. Ref. no. P10654)
Authentication	None of the cells were authenticated.
Mycoplasma contamination	Cells were routinely tested for mycoplasma (once a month) where they were grown in an antibiotic-free medium for one week before mycoplasma testing. All cells used were mycoplasma-free.
Commonly misidentified lines (See ICLAC register)	No commonly misdentified cells were used

Flow Cytometry

Plots

Confirm that:

☒ The axis labels state the marker and fluorochrome used (e.g. CD4-FITC).

☒ The axis scales are clearly visible. Include numbers along axes only for bottom left plot of group (a 'group' is an analysis of identical markers).

☒ All plots are contour plots with outliers or pseudocolor plots.

☒ A numerical value for number of cells or percentage (with statistics) is provided.

Methodology

Sample preparation	Caspase 3/7 flow cytometry analysis of cell death: Immediately after the ES, cells were trypsinised and centrifuged (300g for 5 min) to obtain cell pellet. After washing with PBS, cells were incubated with a dye master mix containing CellEventTM Caspase 3/7 Green Detection Reagent (ThermoFisher Scientific, 1:1000) and Zombie NIR fixable viability stain (BioLegend, 1: 2500) for 30 min. Then the cells were centrifuged at 300 g for 5 min, washed with PBS and fixed with 4% paraformaldehyde. The fluorescence signal of Caspase 3/7 (Excitation / Emission = 511 nm / 523 nm) and Zombie NIR dye (Excitation / Emission = 719 nm / 746 nm), characteristic for apoptotic and necrotic cell population, respectively.
Instrument	ID7000 Spectral Flow Cytometer
Software	Kaluza software (v2.1)
Cell population abundance	Only non aggregated cells were measured
Gating strategy	gating strategy is given in SI figure 14

☒ Tick this box to confirm that a figure exemplifying the gating strategy is provided in the Supplementary Information.
